# Efficacy of Olanzapine in Addition to Standard Triplet Antiemetic Therapy for Cisplatin-Based Chemotherapy: A Secondary Analysis of the J-FORCE Randomized Clinical Trial

**DOI:** 10.1001/jamanetworkopen.2023.10894

**Published:** 2023-05-02

**Authors:** Masakazu Abe, Takuhiro Yamaguchi, Yukiyoshi Fujita, Tomoyasu Nishimura, Koichi Kitagawa, Naoki Inui, Katsuya Hirano, Yukio Sakata, Hirotoshi Iihara, Yuichi Shibuya, Kenichi Suzuki, Kazuhiko Shibata, Kensuke Hori, Haruko Daga, Toshiaki Nakayama, Yasuhiko Sakata, Takako Yanai Takahashi, Sadamoto Zenda, Hironobu Hashimoto

**Affiliations:** 1 Department of Obstetrics and Gynecology, Hamamatsu University School of Medicine, Hamamatsu, Shizuoka, Japan; 2 Division of Biostatistics, Tohoku University Graduate School of Medicine, Sendai, Miyagi, Japan; 3 Division of Pharmacy, Gunma Prefectural Cancer Center, Ōta, Gunma, Japan; 4 Department of Pharmacy, Wakayama Medical University Hospital, Wakayama, Wakayama, Japan; 5 Division of Medical Oncology, Kobe Minimally Invasive Cancer Center, Kobe, Hyōgo, Japan; 6 Department of Clinical Pharmacology and Therapeutics, Hamamatsu University School of Medicine, Hamamatsu, Shizuoka, Japan; 7 Department of Respiratory Medicine, Hyōgo Prefectural Amagasaki General Medical Center, Amagasaki, Hyōgo, Japan; 8 Department of Pharmacy, Hakodate Municipal Hospital, Hakodate, Hokkaido, Japan; 9 Department of Pharmacy, Gifu University Hospital, Gifu, Gifu, Japan; 10 Department of Transplant Surgery, Kōchi Health Science Center, Kōchi, Kōchi, Japan; 11 Department of Clinical Pharmacology, School of Pharmacy, Tokyo University of Pharmacy and Life Sciences, Hachiōji, Tokyo, Japan; 12 Department of Medical Oncology, Kouseiren Takaoka Hospital, Takaoka, Toyama, Japan; 13 Department of Obstetrics and Gynecology, Kansai Rousai Hospital, Amagasaki, Hyōgo, Japan; 14 Department of Medical Oncology, Osaka City General Hospital, Osaka, Japan; 15 Department of Pharmacy, Saitama Cancer Center, Kitaadachi-gun, Saitama, Japan; 16 Department of Pharmacy, Hiroshima City North Medical Center Asa Citizens Hospital, Hiroshima, Hiroshima, Japan; 17 Department of Pharmacy, National Cancer Center Hospital, Tokyo, Japan; 18 Japan Supportive, Palliative and Psychosocial Oncology Group, Tokyo, Japan

## Abstract

**Importance:**

It is unknown whether olanzapine combined with triplet antemetic therapy is effective for all patients undergoing highly emetogenic chemotherapy. A secondary analysis of randomized clinical trials using olanzapine may provide insight into the effectiveness of olanzapine for chemotherapy-induced nausea and vomiting (CINV), including cisplatin.

**Objective:**

To examine the add-on effect of olanzapine according to risk factors for CINV.

**Design, Setting, and Participants:**

This preplanned secondary analysis evaluated results of the J-FORCE trial, a large double-blind, placebo-controlled phase 3 randomized clinical trial conducted in Japan from February 9, 2017, to July 18, 2018. Participants were enrolled from 26 participating hospitals across Japan and included patients aged 20 to 75 years who had a malignant tumor and were cisplatin-naive. The efficacy analysis population of the J-FORCE trial was analyzed according to allocation adjustment factors (sex [male or female], age [≥55 years or <55 years], and cisplatin dose [≥70 mg/m^2^ or <70 mg/m^2^]) and patient-related risk factors (history of motion sickness, drinking habit [defined as alcoholic drinks consumption in excess of occasional drinking], and history of morning sickness during pregnancy). Statistical analysis was performed from February 18 to April 18, 2020.

**Interventions:**

Patients were randomized 1:1 to receive 5 mg of olanzapine or placebo combined with standard triplet antiemetic therapy.

**Main Outcomes and Measures:**

The primary end point was complete response (CR, defined as no vomiting and no use of rescue medication) in the delayed phase (24-120 hours after cisplatin-based chemotherapy administration). Secondary end points were CR, complete control, and total control in the acute, delayed, and overall phases for 6 CINV risk factors as well as time to treatment failure. The CR point estimates and 95% CIs of the differences between groups were calculated, and a Mantel-Haenszel test was performed.

**Results:**

Of the 705 patients (mean [SD] age, 63.0 [9.2] years; 471 males [66.8%]) included in the efficacy analysis population; 581 patients (82.4%) were 55 years or older, and 526 (74.6%) were treated with a cisplatin dose of 70 mg/m^2^ or more. Risk difference (RD) for a CR in the delayed phase was significantly greater in the olanzapine group than the placebo group in males (RD, 12.6% [95% CI, 5.0%-20.1%]; *P* = .001); in females (RD, 14.5% [95% CI, 2.2%-26.3%]; *P* = .02); in those 55 years or older (RD, 11.1% [95% CI, 3.9%-18.2%]; *P* = .003) or younger than 55 years (RD, 23.6% [95% CI, 7.3%-38.3%]; *P* = .005); for a cisplatin dose of 70 mg/m^2^ or more (RD, 13.5% [95% CI, 5.9%-21.0%]; *P* < .001); for those without a history of motion sickness (RD, 13.9% [95% CI, 6.9%-20.6%]; *P* < .001); for those with a drinking habit (RD, 14.9% [95% CI, 6.1%-23.4%]; *P* = .001) or without a drinking habit (RD, 12.0% [95% CI, 2.5%-21.3%]; *P* = .01); and for those with a history of morning sickness during pregnancy (RD, 27.2% [9.7%-42.6%]; *P* = .002). In other subgroups, a delayed CR was higher in the olanzapine group than the placebo group, although not significantly higher.

**Conclusions and Relevance:**

Results of this study suggest a benefit of using 5 mg of olanzapine plus triplet antiemetic therapy to counter CINV regardless of the presence or absence of risk factors.

**Trial Registration:**

University Hospital Medical Information Network Clinical Trials Registry Identifier: UMIN000024676

## Key Points

**Question:**

Does the addition of 5 mg of olanzapine to standard triplet antiemetic therapy benefit patients undergoing cisplatin–based highly emetogenic chemotherapy?

**Findings:**

In this secondary analysis involving 705 patients in a randomized clinical trial, complete response (defined as no vomiting and no use of rescue medication) in the delayed phase (24-120 hours after cisplatin-based chemotherapy initiation) was significantly greater in the olanzapine group than the placebo group, regardless of the presence or absence of risk factors.

**Meaning:**

Findings of this study suggest that adding 5 mg of olanzapine to the standard triplet antiemetic therapy is beneficial for countering nausea and vomiting induced by cisplatin-based chemotherapy.

## Teaser

This secondary analysis of the J-FORCE randomized clinical trial examines the effect of olanzapine plus triplet antiemetic therapy on chemotherapy-induced nausea and vomiting in patients with a malignant tumor, stratified by 6 risk factors.

## Introduction

Olanzapine is an atypical antipsychotic drug that has been shown to be effective against chemotherapy-induced nausea and vomiting (CINV).^1^ The standard antiemetic therapy for cisplatin–based chemotherapy and anthracycline plus cyclophosphamide therapy, which are categorized as highly emetogenic chemotherapy (HEC), is a triplet combination of 5-hydroxytryptamine type 3 (5-HT3) receptor antagonist, neurokinin-1 (NK-1) receptor antagonist, and dexamethasone. However, its antiemetic effect is not completely satisfactory. Complete response (CR; defined as no vomiting and no use of rescue medication) was reported to be 80% to 90% in the acute phase (0-24 hours after the start of chemotherapy) and 60% to 70% in the delayed phase (24-120 hours after the start of chemotherapy).^2,3,4^ Therefore, it has been a challenge to improve the outcome, especially in the delayed phase.

Two double-blind, placebo-controlled phase 3 randomized clinical trials showed that olanzapine combined with triplet antiemetic therapy significantly improved nausea and vomiting compared with triplet therapy alone.^5,6^ In the trial by Navari et al,^5^ 10 mg of olanzapine for anthracycline plus cyclophosphamide therapy and cisplatin-based chemotherapy was used, whereas 5 mg of olanzapine for cisplatin-based chemotherapy was used in the J-FORCE trial (a randomized, double-blind, placebo-controlled phase 3 trial evaluating olanzapine, 5 mg, combined with standard antiemetic therapy for the prevention of CINV in patients receiving cisplatin-based HEC).^6^ In the J-FORCE trial, 710 patients were enrolled and the primary end point of a CR in the delayed phase (24-120 hours after cisplatin-based chemotherapy initiation) was found to be significantly higher in the olanzapine group than the placebo group (79.1% vs 65.8%; *P* < .001). This difference in CR of more than 10% is in line with what the Multinational Association of Supportive Care in Cancer recommends as a significant CR difference.^7^

The combination of 5 to 10 mg of olanzapine with triplet therapy is listed as a standard antiemetic therapy for HEC in the updated antiemetic guideline of the American Society of Clinical Oncology.^8^ Alternatively, Antiemesis version 1.2021 of the National Comprehensive Cancer Network adopts a specific treatment option whereby an antiemetic regimen is selected according to an individual patient’s risk.^9^ Similarly, 5 to 10 mg of olanzapine combined with triplet therapy and 2 types of a 3-drug combination therapy (5-10 mg of olanzapine plus 5-HT3 receptor antagonist plus dexamethasone, or 5-HT3 receptor antagonist plus NK-1 receptor antagonist plus dexamethasone) is a standard antiemetic therapy for HEC, with 5 to 10 mg of olanzapine plus triplet therapy being the most commonly recommended.^10^ In the 2019 update of the Multinational Association of Supportive Care in Cancer and European Society of Medical Oncology antiemetic guideline, olanzapine combined with triplet antiemetic therapy is listed as an option for patients who are refractory to triplet therapy.^11^

Studies have reported that CINV has treatment-related (eg, type and dose of anticancer drugs) and patient-related (eg, young age, female sex, motion sickness experience, morning sickness experience during pregnancy, anxiety, no drinking habit, and history of CINV) risk factors.^12,13,14,15,16^ It has also been proposed that antiemetic therapies be selected according to the CINV risk factors of an individual patient.^17^ Currently, olanzapine combined with triplet therapy is the most effective antiemetic therapy for CINV in patients receiving HEC, but no secondary analysis of randomized clinical trials using olanzapine has been reported to date. Therefore, it is unknown whether olanzapine combined with triplet therapy is effective for all patients undergoing HEC or for which specific patients.

Since, to our knowledge, the J-FORCE was the largest randomized clinical trial of the efficacy of olanzapine, we hypothesized that a secondary analysis of this study could provide important information on the effectiveness of olanzapine for CINV with HEC, including cisplatin. In this preplanned secondary analysis of the J-FORCE trial results, we aimed to examine the add-on effect of olanzapine according to risk factors for CINV.

## Methods

### Overview of the J-FORCE Trial

Details on the design and results of the J-FORCE trial have been published.^6^ Briefly, the J-FORCE trial evaluated the effect of adding 5 mg of olanzapine to a standard triplet antiemetic therapy (palonosetron, aprepitant, and dexamethasone) in patients with malignant solid tumors receiving HEC, including cisplatin (≥50 mg/m^2^). The trial was registered with the University Hospital Medical Information Network Clinical Trials Registry (UMIN000024676) and conducted in accordance with the Declaration of Helsinki^18^ and the Ethical Guidelines for Medical and Health Research Involving Human Subjects of the Japanese government. The trial protocol (Supplement 1) was approved by the ethics committees of all participating hospitals. All participants provided written informed consent. We followed the Consolidated Standards of Reporting Trials (CONSORT) reporting guideline.

The J-FORCE trial, which was conducted in Japan from February 9, 2017, to July 13, 2018, had a superiority study design, which assumed a delayed CR of 65% in the placebo group^4^ and detected whether a delayed CR in the olanzapine group was greater by 10% or more than that in the placebo group. The significance level was set at 2.5% in a 1-sided test, the detection power was set at 80%, and the required number of participants for analysis was calculated to be 329 in each treatment group. Therefore, the planned number of patient registrations was set at 690, with the expectation of 5% ineligible and untreated cases. Patients were randomized 1:1 to receive either 5 mg of olanzapine or placebo using 3 important risk factors for CINV as allocation adjustment factors: sex (male or female), age (≥55 years or <55 years), and cisplatin dose (≥70 mg/m^2^ or <70 mg/m^2^).

Participants were enrolled from 26 hospitals across Japan. All participants were Japanese. Eligible patients were cisplatin-naive, were aged 20 to 75 years, had malignant solid tumors, had Eastern Cooperative Oncology Group Performance Status (ECOG PS) score of 0 to 2 (with 0 indicating being fully active without restriction, and 2 indicating being ambulatory and capable of all self-care but unable to carry out any work activities), maintained organ function, and provided written informed consent to participate in the study. Patients with nausea and vomiting requiring antiemetic drugs, ascites requiring therapeutic puncture, gastrointestinal obstruction, diabetes requiring use of insulin or oral hypoglycemic agents, a hemoglobin A_1c_ level of 6.5% or higher (to convert to the proportion of total hemoglobin, multiply by 0.01), and a smoking status were excluded.

In addition to standard antiemetic therapy for HEC (palonosetron: 0.75 mg on day 1; aprepitant: 125 mg on day 1 and 80 mg on days 2 to 3; dexamethasone: 12 mg on day 1 and 8 mg on days 2 to 4), the study drugs (5 mg of olanzapine or placebo) were administered after dinner on days 1 to 4. Patients recorded the degree of nausea (using a 4-point categorical scale: none, mild, moderate, or severe), the presence and frequency of vomiting, and rescue medication in their symptom diary every 24 hours from the start of cisplatin-based chemotherapy administration to 120 hours later. Additionally, patients described 3 risk factors that are typical for nausea and vomiting, such as history of motion sickness, drinking habit (defined as alcoholic drinks consumption in excess of occasional drinking), and history of morning sickness during pregnancy (only for females), in their symptom diary.

The primary end point was CR in the delayed phase (24-120 hours after cisplatin-based chemotherapy initiation). Secondary end points were CR in the acute (from cisplatin-based chemotherapy initiation to 24 hours) and overall (from cisplatin-based chemotherapy initiation to 120 hours) phases; complete control (CC, defined as no more than mild nausea under CR); total control (TC, defined as no nausea under CR) in the acute, delayed, and overall phases; and time to treatment failure (TTF, defined as time from cisplatin-based chemotherapy initiation to the first emesis or use of rescue medication), among other outcomes.

### Statistical Analysis

We analyzed CR, CC, TC, and TTF in the acute, delayed, and overall phases for all of the following 6 items of CINV risk factors: sex, age, and cisplatin dose (which were defined as allocation adjustment factors) and history of motion sickness, drinking habit, and history of morning sickness during pregnancy (as collected from the symptom diary). For CR, CC, and TC, the point estimates and 95% CIs of the differences between groups were calculated, and a Mantel-Haenszel test was performed. The Kaplan-Meier method was used to calculate median estimates and 95% CIs of TTF in each group, and a log-rank test was used for intergroup comparisons. A forest plot of delayed CR in subgroups was used to analyze the risk difference (RD) for the 6 risk factors, ECOG PS score (0 vs 1 to 2), and each cancer type (head and neck, esophageal, lung, gastric, and gynecological).

Two-sided *P* < .05 indicated significance. Statistical analyses were performed from February 18 to April 18, 2020, using SAS, version 9.4 (SAS Institute Inc).

## Results

The secondary analysis included 705 patients (mean [SD] age, 63.0 [9.2] years; 234 females [33.2%] and 471 males [66.8%]) in the efficacy analysis set, of the total 710 patients enrolled in the J-FORCE trial (eFigure 3 in Supplement 2). Patient characteristics are shown in the Table. In both the olanzapine and placebo groups, 33% of patients were females, 581 patients (82.4%) were 55 years or older, and 526 patients (74.6%) were treated with a cisplatin dose of 70 mg/m^2^ or more. Concerning the prevalence of risk factors in both groups, 18.5% of patients had a history of motion sickness, 47.5% had a drinking habit, and 54.5% of female patients had a history of morning sickness during pregnancy.

**Table.** Patient Characteristics

Characteristic	Patients, No. (%) (N = 705)	
	Olanzapine (n = 354)	Placebo (n = 351)
Sex		
Male	237 (67)	234 (67)
Female	117 (33)	117 (33)
Age		
≥55 y	291 (82)	290 (83)
<55 y	63 (18)	61 (17)
Cisplatin dose		
<70 mg/m^2^	90 (25)	89 (25)
≥70 mg/m^2^	264 (75)	262 (75)
ECOG PS score		
0	227 (64)	214 (61)
1	121 (34)	136 (39)
2	6 (2)	1 (0.3)
Cancer type		
Head and neck	33 (9)	25 (7)
Esophageal	74 (21)	79 (23)
Lung	179 (50)	183 (52)
Gastric	20 (6)	19 (5)
Gynecological	34 (10)	34 (10)
Other^a^	14 (4)	11 (3)
Risk factor		
History of motion sickness	67 (19)	64 (18)
Drinking habit	160 (45)	174 (50)
History of morning sickness during pregnancy^b^	71 (61)	55 (48)

Abbreviation: ECOG PS, Eastern Cooperative Oncology Group Performance Status.

^a^ Other types included urological, skin, and pleural mesothelioma.

^b^ Female only (percentage of female participants).

### Secondary Analysis by Allocation Adjustment Factors

**Figure 1.**
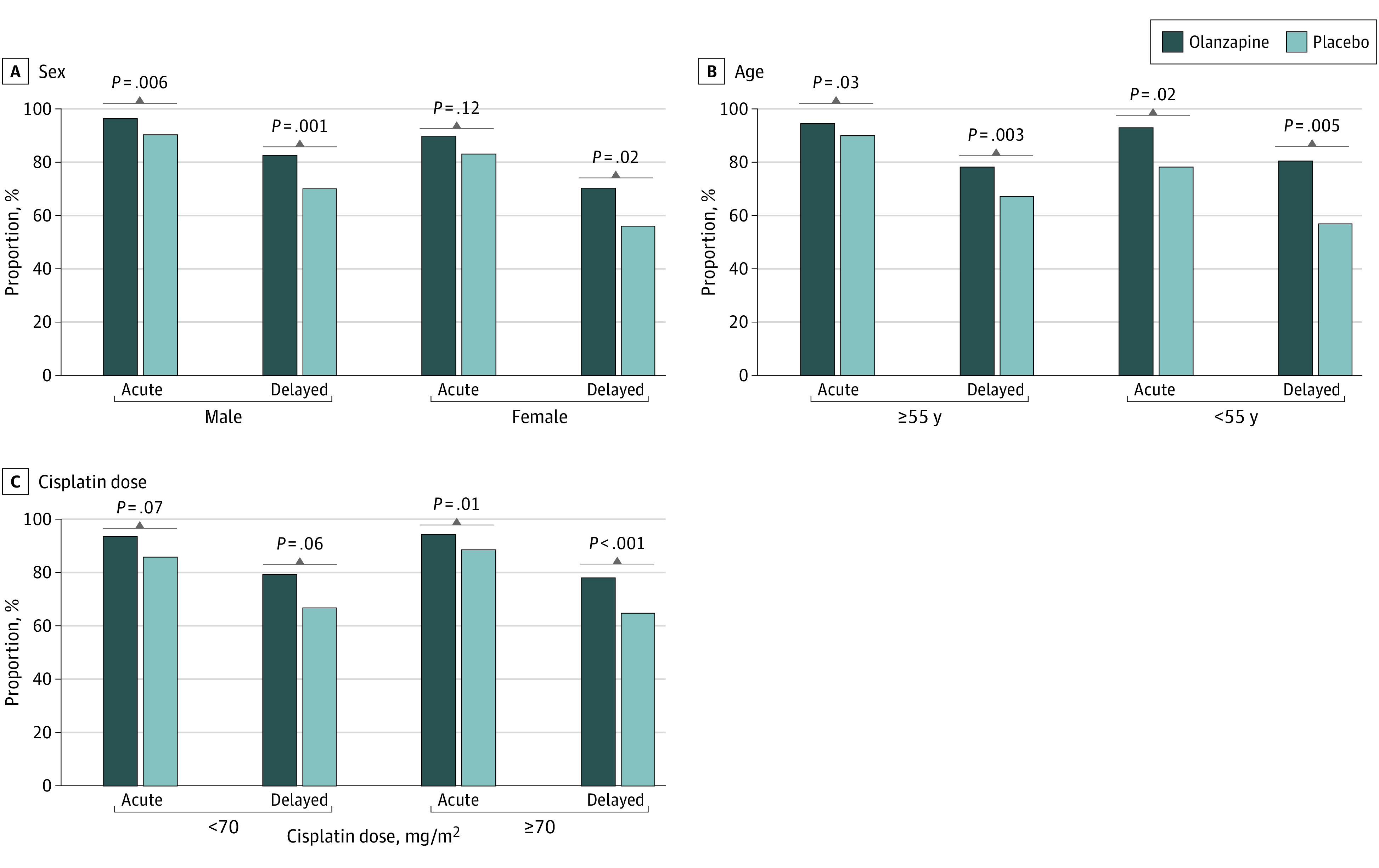
The Proportion of Patients Achieving a Complete Response by Allocation Adjustment Factors

Acute and delayed CRs according to the allocation adjustment factors are shown in Figure 1. All data for CR, CC, and TC by allocation adjustment factors are shown in eTable 1 in Supplement 2. In both males and females, delayed CR was significantly higher for the olanzapine group than for the placebo group (RD, 12.6% [95% CI, 5.0%-20.1%]; *P* = .001 for males and 14.5% [95% CI, 2.2%-26.3%] for females; *P* = .02). Additionally, the proportion of patients showing a delayed CR was lower for females than males in both olanzapine and placebo groups (Figure 1A). The RDs for delayed CR between males and females were similar in both the olanzapine and placebo groups (RD, 12.2% [95% CI, 2.7%-21.7%] for the olanzapine group vs 14.1% [95% CI, 3.4%-24.8%] for the placebo group; *P* = .79). For the age secondary analysis, delayed CR was significantly higher in the olanzapine group than the placebo group for both the groups aged 55 years or older (RD, 11.1% [95% CI, 3.9%-18.2%]; *P* = .003) and younger than 55 years (23.6% [95% CI, 7.3%-38.3%]; *P* = .005). The RDs for delayed CR between patients 55 years or older and those younger than 55 years were smaller for the olanzapine group than the placebo group, although not significantly (RD, −2.3% [95% CI, −13.0% to 8.5%] for the olanzapine group vs 10.2% [95% CI, −3.3% to 23.7%] for the placebo group; *P* = .16). The add-on effect of olanzapine was greater in patients younger than 55 years, indicating that the addition of olanzapine reduced the difference in risk by age (Figure 1B). In the cisplatin dose secondary analysis, a delayed CR was significantly higher in the olanzapine group than the placebo group for a cisplatin dose of 70 mg/m^2^ or more (RD, 13.5% [95% CI, 5.9%-21.0%]; *P* < .001). No significant difference was observed in delayed CR between the olanzapine and placebo groups for a cisplatin dose less than 70 mg/m^2^ (RD, 12.6% [95% CI, −0.3% to 25.0%]; *P* = .06), but the RD was similar to that for a cisplatin dose of 70 mg/m^2^ or more (Figure 1C). The RDs for delayed CR and CC between olanzapine and placebo groups were greater than 10% (11.1%-28.5%) in all subgroups (eTable 1 in Supplement 2).

The TTF was significantly longer in the olanzapine group than the placebo group in all subgroups: males (hazard ratio [HR], 0.49; 95% CI, 0.34-0.73; *P* < .001), females (HR, 0.61; 95% CI, 0.40-0.93; *P* = .02), 55 years or older (HR, 0.59; 95% CI, 0.43-0.80; *P* < .001), younger than 55 years (HR, 0.40; 95% CI, 0.21-0.76; *P* = .004), cisplatin dose less than 70 mg/m^2^ (HR, 0.54; 95% CI, 0.31-0.95; *P* = .03), and cisplatin dose 70 mg/m^2^ or more (HR, 0.55; 95% CI, 0.39-0.76; *P* < .001) (eFigure 1 in Supplement 2).

### Secondary Analysis by Patient-Related Risk Factors

**Figure 2.**
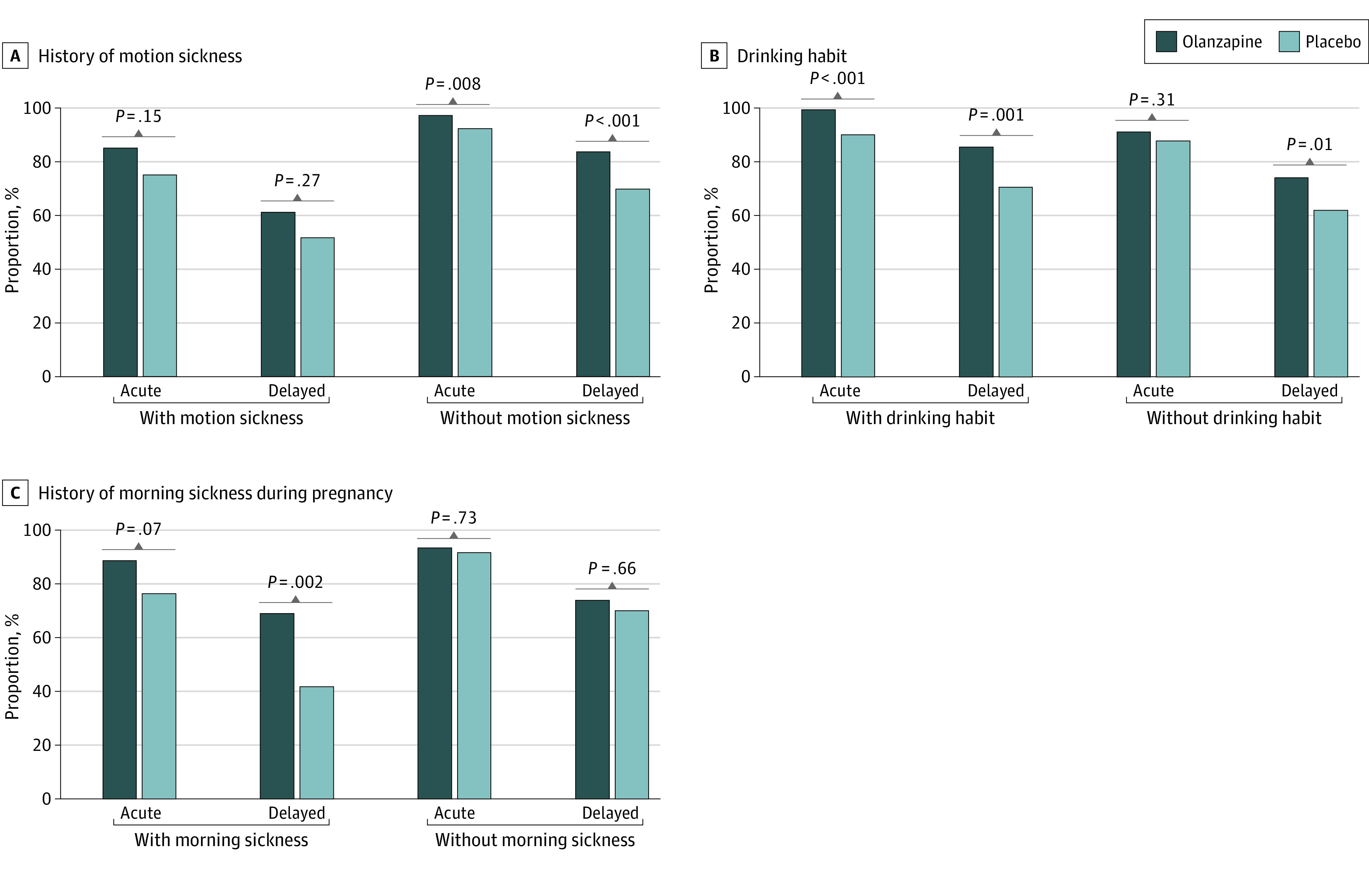
The Proportion of Patients Achieving a Complete Response by Patients’ Risk Factors

Acute and delayed CRs with or without patient-related risk factors are shown in Figure 2. All data for CR, CC, and TC according to patient-related risk factors are shown in eTable 2 in Supplement 2. For patients without a history of motion sickness, the delayed CR was significantly higher in the olanzapine group than the placebo group (RD, 13.9% [95% CI 6.9%-20.6%]; *P* < .001) (Figure 2A). The RDs for delayed CR between patients with vs without a history of motion sickness were similar in both the olanzapine and placebo groups (RD, 22.4% vs 18.2%; *P* = .65). In patients with vs without a drinking habit, the delayed CR was significantly higher in the olanzapine group than the placebo group (RD, 14.9% [95% CI, 6.1%-23.4%] with a drinking habit; *P* = .001 and 12.0% [95% CI, 2.5%-21.3%] without drinking habit; *P* = .01) (Figure 2B). The RDs for delayed CR between patients with vs without a drinking habit were similar in both the olanzapine and placebo groups (RD, 11.5% [95% CI, 3.3%-19.8%] for the olanzapine group vs 8.6% [95% CI, −1.3% to 18.5%] for the placebo group; *P* = .66). In patients with a history of morning sickness during pregnancy, delayed CR was significantly higher in the olanzapine group than the placebo group (RD, 27.2% [95% CI, 9.7%-42.6%]; *P* = .002) (Figure 2C). The RDs for delayed CR between patients with vs without a history of morning sickness during pregnancy tended to be smaller for the olanzapine group than the placebo group (RD, 4.9% [−11.7% to 21.5%] for the olanzapine group vs 28.2% [95% CI, 10.7%-45.6%] for the placebo group; *P* = .06), indicating that the addition of olanzapine reduced the difference in risk due to morning sickness during pregnancy. The proportions of patients with CC in both acute and delayed phases in the olanzapine group (87.3% and 69.0%, respectively) were lower in those with history of morning sickness during pregnancy. However, the RDs for CC were higher in the olanzapine group vs the placebo group in patients with a history of morning sickness during pregnancy (RD, 12.3% [95% CI, −0.9% to 26.9%] in the acute phase; 27.2% [95% CI, 9.7%-42.6%] in the delayed phase), the RD between both those with and those without morning sickness during pregnancy were smaller (RD, 4.0% [95% CI, −7.3% to 15.2%) in the acute phase and 4.9% [95% CI, −11.7% to 21.5%] in the delayed phase) (eTable 2 in Supplement 2).

The TTF was significantly longer in the olanzapine group than the placebo group in patients without a history of motion sickness (HR, 0.48; 95% CI, 0.34-0.68; *P* < .001), with a drinking habit (HR, 0.44; 95% CI, 0.27-0.71; *P* < .001), without a drinking habit (HR, 0.61; 95% CI, 0.43-0.88; *P* = .007), and with a history of morning sickness during pregnancy (HR, 0.43; 95% CI, 0.26-0.72; *P* = .001) (eFigure 2 in Supplement 2).

### Subgroups With Delayed CR

**Figure 3.**
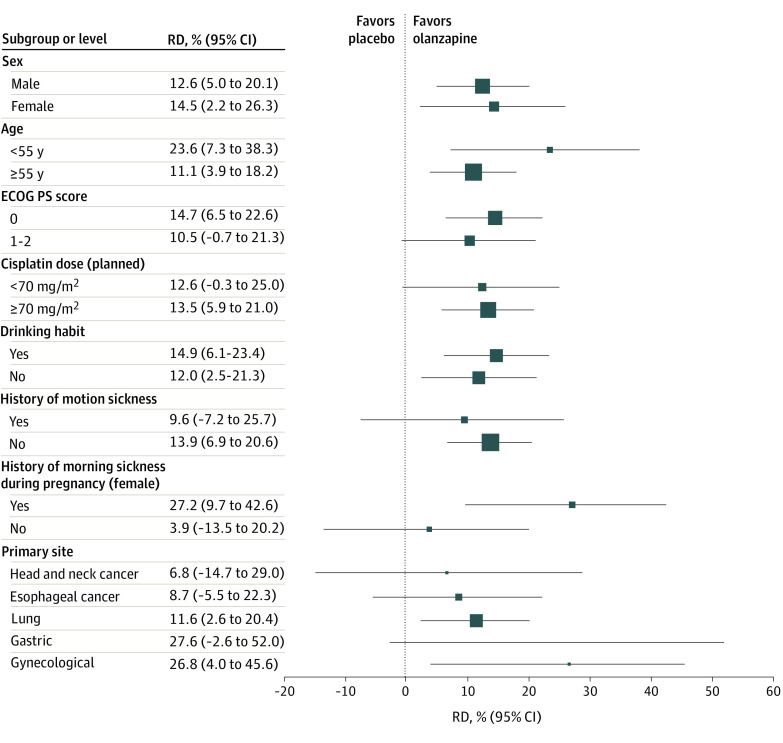
Forest Plot of the Risk Difference (RD) in a Delayed Complete Response by Subgroup ECOG PS indicates Eastern Cooperative Oncology Group Performance Status. The different sizes of the boxes represent the number of patients.

The RD of a delayed CR, which is the primary end point, was significantly greater in the olanzapine group than in the placebo group for these subgroups: males, females, age 55 years or older, age younger than 55 years, cisplatin dose of 70 mg/m^2^ or more, with a drinking habit, without a drinking habit, without a history of motion sickness, with a history of morning sickness during pregnancy, ECOG PS score of 0, lung cancer, and gynecological cancer. In the other subsets, RD was not significantly different between the groups but tended to be greater in the olanzapine group than the placebo group (Figure 3).

## Discussion

To our knowledge, this secondary analysis was the first to examine the add-on effect of olanzapine according to risk factors for CINV in the J-FORCE trial. Six major risk factors for CINV were analyzed.

As shown in Figure 3, an add-on effect of olanzapine on a delayed CR in all subgroups occurred regardless of risk factors. In particular, a delayed CR was significantly higher in the olanzapine group than the placebo group for the following subgroups: male, female, age 55 years or older, age younger than 55 years, cisplatin dose of 70 mg/m^2^ or more, with and without a drinking habit, without a history of motion sickness, and with a history of morning sickness during pregnancy. In the analysis by allocation adjustment factors, the outcomes of the olanzapine group were significantly better than those of the placebo group for most factors. Additionally, the RDs for delayed CR and CC between olanzapine and placebo groups ranged from 11.1% to 28.5% in all subgroups. Experiencing CINV during initial chemotherapy has been associated with increased risk of anticipatory nausea and vomiting during subsequent chemotherapy, which in turn results in poor control of nausea and vomiting, depending on its severity, and further aggravates this control with each successive treatment cycle.^13,14,19^ Therefore, for better control of nausea and vomiting during the entire treatment cycle, findings of this study suggest that adding olanzapine to triplet antiemetic therapy at the initiation of chemotherapy is beneficial regardless of risk factors, unless olanzapine is difficult to administer.

The addition of olanzapine normalized the RDs across age groups and between patients with vs without a history of morning sickness during pregnancy. In the placebo group, the proportion of patients showing a CR and CC in both acute and delayed phases was lower for those younger than 55 years who were at high risk of CINV. However, the proportions of patients showing a CR and CC were the same between those 55 years or older or younger than 55 years in the olanzapine group. The RD between olanzapine and placebo groups for delayed CR and CC was approximately 2-fold higher in patients younger than 55 years than those 55 years or older, suggesting that the addition of olanzapine reduced the difference in risk by age. Regarding morning sickness during pregnancy, the proportion of patients showing a CR and CC in both acute and delayed phases (RD, 15.3% and 28.2%) was lower in those with a history of morning sickness during pregnancy, who were at higher risk of CINV. However, since the proportion of patients showing a CR and CC was higher in the olanzapine vs placebo group in patients with a history of morning sickness during pregnancy in the acute (RD, 12.3%-12.4%) and delayed (RD, 27.2%) phases, the RD became smaller (4.0%-4.9%).

Concerning a report that the RD was reduced by adding a new antiemetic, RD by sex decreased with the addition of aprepitant.^16,19^ However, according to the results of the J-FORCE and TRIPLE (a randomized, double-blind, phase 3 trial of palonosetron vs granisetron in the triplet regimen for preventing CINV after HEC) trials,^4,6^ the outcomes of triplet antiemetic therapy including aprepitant were poorer in females than in males, and it is doubtful that the use of aprepitant reduces the RD between sexes. In the secondary analysis of the J-FORCE trial, the addition of olanzapine increased the proportion of males and females showing a CR and CC, but the RD for TC between males and females did not decrease by adding olanzapine. We believe these results were due to the poor outcome in females who had a history of morning sickness during pregnancy. In the J-FORCE trial, 54.5% of females had a history of morning sickness during pregnancy. As shown in eTable 2 in Supplement 2, a 27.2% RD was apparent in the proportion of females showing delayed CR and CC in the olanzapine and placebo groups who had a history of morning sickness during pregnancy. Additionally, the outcomes in females who had experienced morning sickness during pregnancy were comparable with the outcomes in males in the placebo group even with the addition of olanzapine, although the effect of adding olanzapine was substantial. This finding might be the reason that the RD between males and females did not become smaller, even with the addition of olanzapine. Although using olanzapine did not reduce the RD between males and females, it might have decreased the RD between age groups and between females with vs without a history of morning sickness during pregnancy.

### Limitations

A limitation of this analysis was that risk factors other than the allocation adjustment factors were not used to randomize participants to olanzapine and placebo groups. Additionally, since the J-FORCE trial did not include patients who were treated with an anthracycline plus cyclophosphamide regimen, it is unclear whether the findings would apply to this regimen.

## Conclusions

Results of this secondary analysis suggest that treating patients with 5 mg of olanzapine plus triplet antiemetic therapy from the time of initial chemotherapy is beneficial for countering CINV regardless of the presence or absence of risk factors. Moreover, the addition of olanzapine might reduce the RD by age and history of pregnancy-related illness, which are typical risk factors for CINV.

## Notes

Supplement 1.
Trial Protocol and Statistical Analysis Plan

Supplement 2. **eFigure 1.** Kaplan-Meier Plot Showing the Time to Treatment Failure According to Allocation Adjustment Factors **eFigure 2.** Kaplan-Meier Curve Showing the Time to Treatment Failure According to Patients’ Risk Factors **eFigure 3.** CONSORT Diagram **eTable 1.** The Proportion of Patients Achieving a Complete Response, Complete Control, and Total Control According to Allocation Adjustment Factors **eTable 2.** The Proportion of Patients Achieving a Complete Response, Complete Control, and Total Control According to Patients’ Risk Factors

Supplement 3.
Data Sharing Statement
