# Supplementary material for: Efficacy of Olanzapine in Addition to Standard Triplet Antiemetic Therapy for Cisplatin-Based Chemotherapy: A Secondary Analysis of the J-FORCE Randomized Clinical Trial
Source: JAMA Netw Open. 2023 May 2;6(5):e2310894. doi: 10.1001/jamanetworkopen.2023.10894 (PMC10155068; doi:10.1001/jamanetworkopen.2023.10894)
Supplement: Supplement 1. — Trial Protocol and Statistical Analysis Plan [file jamanetwopen-e2310894-s001.pdf]

Japan Agency for Medical Research and Development (AMED)--granted R&D Expense
For Research Project on Practical application of Innovative Cancer Medicine
"Development of a new antiemetic therapy for preventing chemotherapy-induced nausea and vomiting"

A randomized, double-blind, placebo-controlled phase III trial evaluating olanzapine 5 mg combined with standard antiemetic therapy for the prevention of chemotherapy-induced nausea and vomiting in patients receiving cisplatin-based highly emetogenic chemotherapy

J-SUPPORT1604（NCCH1604）

STUDY PROTOCOL

J-FORCE STUDY

J-SUPPORT and the Fourth agent “Olanzapine” Resist Cisplatin Emetogenecity.

Japan Supportive, Palliative and Psychosocial Oncology Group (J-SUPPORT) Study.
(UMIN ID: UMIN000024676)

Principal Investigator: Masakazu Abe, M.D.
Department of Gynecologic Oncology, Shizuoka Cancer Center
1007 Shimonagakubo, Nagaizumi-cho, Sunto-gun, Shizuoka Prefecture 411-8777, Japan
Phone: +81-55-989-5222 Ext. 6202
Fax: +81-55-989-5551
E-mail: ma.abe@scchr.jp

Co-principal Investigator: Hironobu Hashimoto, Assistant Director
Department of Pharmacy, National Cancer Center Hospital
Tsukiji 5-1-1, Chuo-ku, Tokyo, 104-0045, Japan
Phone: +81-3-3542-2511 Ext. 3531
Fax: +81-3-3248-0730
E-mail: hhashimo@ncc.go.jp

Approved by the Concept study meeting of the National Cancer Center Hospital on March 22, 2016
Approved by the Protocol Concept Committee of the J-SUPPORT on April 9, 2016
Approved by the Protocol Review Committee of the J-SUPPORT on August 26, 2016

Version 1.0: November 1, 2016

0 Synopsis

0.1. Schema

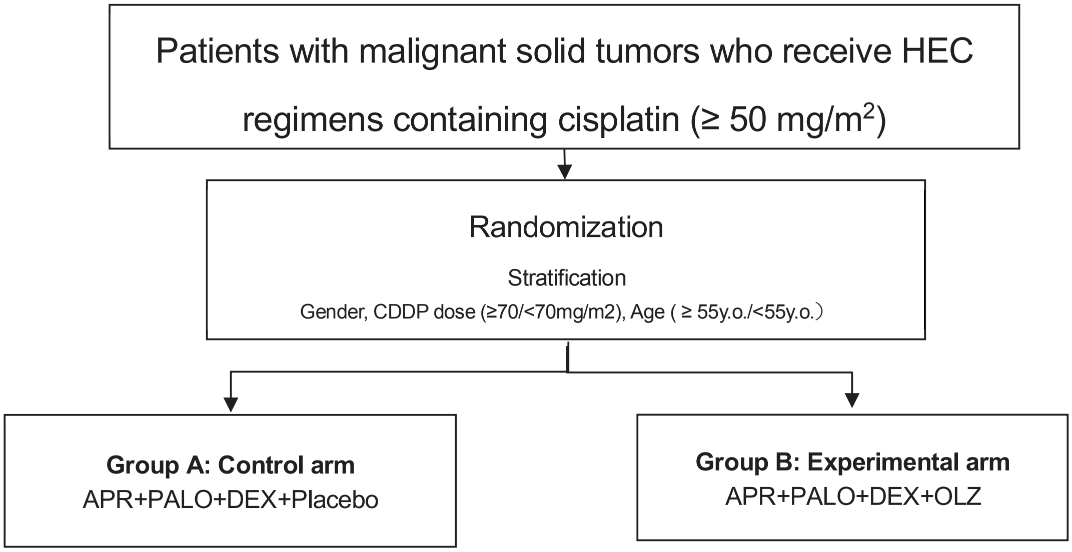

APR: Aprepitant, PALO: Palonosetron, DEX: Dexamethasone, OLZ: Olanzapine

0.2. Objectives

This study aims to confirm that the addition of olanzapine (OLZ) to a combination of aprepitant (APR), palonosetron (PALO) and dexamethasone (DEX), which is the standard antiemetic therapy for highly emetogenic chemotherapy (HEC) containing cisplatin (CDDP) (≥ 50 mg/m^2), significantly improves the complete response rate (CR rate) in the delayed phase.

Primary endpoint

- The CR rate in the delayed phase (24–120 hours after the initiation of CDDP administration).

※ Complete response is defined as a condition in which a patient does not show vomiting or retching and does not require additional treatment with antiemetics. Retching is defined as the physical reaction that accompanies vomiting in the absence of gastric content ejection.

Secondary endpoints

- CR rate during the acute phase (0–24 hours from the initiation of CDDP administration) and the overall phase (0–120 hours from the initiation of CDDP administration)
- Complete control rate (CC rate) during the overall phase and every 24 hours
- Total control rate (TC rate) during the overall phase and every 24 hours
- Time to treatment failure (TTF)
- Severity of nausea (patient self-evaluation according to a 4-grade categorical scale)
- Severity of anorexia (patient self-evaluation according to a 4-grade categorical scale)
- Severity of drowsiness and effects on daily life (patient self-evaluation according to a 4-grade categorical scale)
- Incidence of adverse events

※ Complete control is defined as a condition where a patient shows no nausea (“0” on a 4-grade categorical scale) or mild nausea (“1” on a 4-grade categorical scale) under CR.

※ Total control is defined as a condition where a patient neither shows nausea (“0” on a 4-grade categorical scale) nor vomiting under CR.

0.3. Subjects

Subjects are patients with solid malignancies who were indicated for initial treatment with a HEC regimen containing CDDP (50 mg/m^2 or more) and the standard antiemetic therapy (APR + PALO + DEX).

0.4. Treatment

Control arm (Group A): APR+PALO+DEX+Placebo

< In cases where APR will be orally administered (When Emend® capsules are used) >

Group	Therapeutic regimen				
	Drug	Day 1	Day 2	Day 3	Day 4
Group A (Placebo group)	Placebo (p.o.)	Placebo	Placebo	Placebo	Placebo
	APR (p.o.)	125 mg	80 mg	80 mg	-
	PALO (i.v.)	0.75 mg	-	-	-
	DEX (i.v. or p.o.)	12 mg	8 mg	8 mg	8 mg

<In cases where APR will be intravenously administered (When Proemend® for intravenous infusion is used) >

Group	Therapeutic regimen				
	Drug	Day 1	Day 2	Day 3	Day 4
Group A (Placebo group)	Placebo (p.o.)	Placebo	Placebo	Placebo	Placebo
	APR (i.v.)	150 mg	-	-	-
	PALO (i.v.)	0.75 mg	-	-	-
	DEX (i.v. or p.o.)	12 mg	8 mg	16 mg	16 mg

Experimental arm (Group B): APR+PALO+DEX+OLZ

< In cases where APR will be orally administered (When Emend® capsules are used) >

Group	Therapeutic regimen				
	Drug	Day 1	Day 2	Day 3	Day 4
Group B (OLZ group)	OLZ (p.o.)	5 mg	5 mg	5 mg	5 mg
	APR (p.o.)	125 mg	80 mg	80 mg	-
	PALO (i.v.)	0.75 mg	-	-	-
	DEX (i.v. or p.o.)	12 mg	8 mg	8 mg	8 mg

<In cases where APR will be intravenously administered (When Proemend® for intravenous infusion is used) >

Group	Therapeutic regimen				
	Drug	Day 1	Day 2	Day 3	Day 4
Group B (OLZ group)	OLZ (p.o.)	5 mg	5 mg	5 mg	5 mg
	APR (i.v.)	150 mg	-	-	-
	PALO (i.v.)	0.75 mg	-	-	-
	DEX (i.v. or p.o.)	12 mg	8 mg	16 mg	16 mg

- OLZ/Placebo: OLZ at 5 mg or placebo will be administered once daily **after dinner** from Day 1 to Day 4 during the CDDP administration period.

.

- APR: < Oral administration > On Day 1, one APR capsule (125 mg) will be administered at least 60 minutes before CDDP administration. On Days 2 and 3, one APR capsule (80 mg) will be administered after breakfast or until noon.
  < Intravenous administration > APR for intravenous infusion will be administered at least 60 minutes prior to CDDP administration.

• PALO: PALO will be administered at least 30 minutes before CDDP administration.

• DEX: Prescribed doses of DEX will be administered at least 30 minutes prior to CDDP administration. Oral administration will be acceptable instead of intravenous infusion.

※ For DEX usage, the drug will be taken orally after breakfast or after lunch once daily and after breakfast and after lunch twice daily.

※ Patients who continue to be treated with low risk anticancer agents until Day 5 (esophageal cancer: FP therapy, etc.), will be allowed to continue with DEX administration up to Day 5 as prescribed in the regimen in advance. (When APR is orally administered, the dose of intravenous DEX will be 8 mg; and when APR is administered intravenously, the dose of infused DEX will be 16 mg.

0.5. Planned number enrollments and research period

Planned number of enrollments	Group A (Placebo group), 345 patients;<br>Group B (OLZ 5 mg group), 345 patients
Registration period	From January 2017 through January 2019 (for 2 years)
Research period	From January 2017 through June 2019 (for 2.5 years)

0.6. Contact address

Study Secretariat : Hironobu Hashimoto
Department of Pharmacy, National Cancer Center Hospital
Adress: Tsukiji 5-1-1, Chuo-ku, Tokyo, 104-0045, Japan
Phone: +81-3-3542-2511 Ext. 3531
Fax: +81-3-3248-0730
E-mail: hhashimo@ncc.go.jp

Table of contents

0 Synopsis 1
0.1. Schema 1
0.2. Objectives 1
0.3. Subjects 2
0.4. Treatment 2
0.5. Planned number enrollments and research period 3
0.6. Contact address 3
1. Objectives 8
2. Background and rationale for the study design 8
2.1. Subjects 8
2.2. Standard treatment for subjects 10
2.3. Rationale for setting the treatment plan 11
2.4. Study design 14
2.5. Summary of the expected benefits and disadvantages associated with participating in the trial 16
2.6. Significance of this study 16
2.7. Additional study 16
3. Criteria and definitions used in this study 16
3.1. Definition of "High emetic risk" 17
4. Patient selection criteria 17
4.1. Eligibility criteria (Inclusion criteria) 17
4.2. Exclusion criteria 18
5. Registration and allocation 18
5.1. Registration procedures 18
5.2. Notes on registration 19

5.3. Random allocation and stratification factors 19

5.4. Blinding 19

5.5. Preparation of study drugs 20

6. Treatment plan and treatment change criteria 21

6.1. Protocol treatment 21

6.2. Unacceptable combination therapy 22

6.3. Discontinuation and completion criteria of the protocol treatment 23

6.4. After-treatment 24

7. Expected adverse effects 24

7.1. Adverse effects by treatment group 24

7.2. Evaluation of adverse events / adverse effects 25

8. Evaluation items, laboratory examinations, and evaluation schedule 25

8.1. Before the treatment initiation 25

8.2. Protocol treatment period 26

8.3. Study calendar 27

9. Data collection 28

9.1. Recording forms (Electronic Case Report Form: eCRF) 28

10. Report of adverse events 28

10.1. Adverse events requiring with reporting 29

10.2. Reporting obligation and reporting procedures of institutional research director 30

10.3. Responsibility of the Study Representative/ Study Secretariat 31

10.4. Response of principal investigators of participating institutions (including relevant institution) 32

10.5. Response of the director of the institution to the occurrence of an adverse event 32

10.6. Review by the Independent Data Monitoring Committee................................ 32
11. Determination of the efficacy and definition of endpoints 33
11.1. Determination of the efficacy 33
11.2. Definition of endpoints.. 34
12. Statistical matters 35
12.1. Analysis sets.. 35
12.2. Analyses of the primary endpoint.. 36
12.3. Analyses of the secondary endpoints for the efficacy.................................... 36
12.4. Safety analysis.. 36
12.5. Rationale for setting planned number of enrollments..................................... 36
12.6. Interim analysis.. 36
13. Ethical matters.. 36
13.1. Protection of patients... 36
13.2. Informed consent 37
13.3. Protection of personal information and patient identification........................ 38
13.4. Compliance with the protocol 38
13.5. Approval of the Ethics Review Committee of medical institution................... 38
13.6. Contents change of the protocol 39
13.7. Management of conflict of interest (COI)... 40
13.8. Compensation.. 40
13.9. Intellectual property 40
13.10. Information disclosure on this study... 41
14. Monitoring and auditing 41
14.1. Periodic monitoring... 41
14.2. Facility visit audit.. 43
15. Additional notes 43

15.1. Off-label use of drugs 43
16. Research organization / funding source 43
16.1. Main funding source for this study 43
16.2. J-SUPPORT 43
16.3. Representative of J-SUPPORT 43
16.4. J-SUPPORT Administrative Office 44
16.5. Study Representative 44
16.6. Study Secretariat 44
16.7. Statistical analysis personnel 44
16.8. Participating Institutions 45
16.9. Independent Data Monitoring Committee 46
16.10. Auditing committee 46
16.11. Data Center 46
16.12. Protocol preparation 46
17. Publication of research results 46
17.1. Main paper 46
17.2. Supplementary paper 47
17.3. First presenter of academic presentation etc. 47
18. References 49

1. Objectives

This study aims to confirm that the addition of olanzapine (OLZ) to a combination of aprepitant (APR), palonosetron (PALO) and dexamethasone (DEX), which is the standard antiemetic therapy for highly emetogenic chemotherapy (HEC) containing cisplatin (CDDP) (≥ 50 mg/m2), significantly improves the complete response rate (CR rate) in the delayed phase.

Primary endpoint

- The CR rate in the delayed phase (24–120 hours after the initiation of CDDP administration)
  ※ Complete response is defined as a condition in which a patient does not show vomiting or retching and does not require additional treatment with antiemetics. Retching is defined as the physical reaction that accompanies vomiting in the absence of gastric content ejection.

Secondary endpoints

- CR rate during the acute phase (0–24 hours from the initiation of CDDP administration) and the overall phase (0–120 hours from the initiation of CDDP administration)
- Complete control rate (CC rate) during the overall phase and every 24 hours
- Total control rate (TC rate) during the overall phase and every 24 hours
- Time to treatment failure (TTF)
- Severity of nausea (patient self-evaluation according to a 4-grade categorical scale)
- Severity of anorexia (patient self-evaluation according to a 4-grade categorical scale)
- Severity of drowsiness and its life impact (patient self-evaluation according to a 4-grade categorical scale)
- Incidence of adverse events
  ※ Complete control is defined as a condition where a patient shows no nausea (“0” on a 4-grade categorical scale) or mild nausea (“1” on a 4-grade categorical scale) under CR.
  ※ Total control is defined as a condition where a patient neither shows nausea (“0” on a 4-grade categorical scale) nor vomiting under CR.

2. Background and rationale for the study design

2.1. Subjects

Epidemiology

"Nausea" is defined as "the discomfort experienced when a patient is likely to vomit", and "vomiting" is defined as "physical movements that are initiated in order to forcibly eject stomach contents". Physiologically, vomiting is induced by gastric contents being excreted due to closure of gastric pylorus, relaxation of the fundus of the stomach and the lower esophageal sphincter, and contraction of the diaphragm and abdominal muscles.

Chemotherapy-induced nausea and vomiting (CINV) is one of the most unpleasant adverse events for patients. The incidence of CINV can exceed 90% when antiemetic therapy is not applied to patients treated with anticancer agents classified as HECs. This statistic is based on pooled analysis of four clinical trials carried out in the 1980s and 1990s[1], and is similarly described in guidelines from the NCCN, MASCC, ASCO, and the Japan Society of Clinical Oncology. These data indicate that failure to include measures against nausea and vomiting for patients treated with HEC will significantly reduce quality of life and can compromise future treatment regimens. Hence, management of nausea and vomiting is critical for providing safe and effective cancer treatment[2].

Pathogenic mechanism of CINV

Vomiting is caused by the stimulation of multilevel reflex pathways controlled in the brain. The stimulus conduction pathway to the vomiting center consists of the cerebral cortex (e.g., increased intracranial pressure, tumor, vascular lesions, emotion), chemoreceptors (e.g., metabolites, hormones, drugs, toxins), vestibular apparatus (e.g., posture, gyration, vestibular lesion), and periphery (e.g., mechanical receptors in the digestive tract including the pharynx, the heart and abdominal organs; and chemoreceptors in the gastrointestinal tract). The process of vomiting occurs in the following stages. First, nausea is induced by the excitation of the vomiting center in the media oblongata due to complex

stimulation of $5HT_3$ receptors, predominantly located in the upper gastrointestinal tract, and neurokinin-1 (NK1) receptors in the chemotherapy trigger zone (CTZ) in the fourth ventricle. Vomiting then occurs by efferent stimulation transmitted from the vomiting center to the salivary secretory center, abdominal muscles, respiratory center, and cranial nerve [3,4].

Brief overview of standard treatment by emetic risk and efficacy

The incidence of nausea/vomiting induced by anticancer pharmacotherapy is greatly affected by the emetogenicity of the anticancer drug used. Drugs fall into one of four categories: highly emetogenic, moderately emetogenic, mildly emetogenic and minimally emetogenic. Classification is determined according to the incidence of nausea / vomiting that occurs within 24 hours of administration of an anti-cancer drugs in the absence of antiemetic drug prophylaxis.

Classification of standard antiemetic therapies based on their emetogenic risk is shown in the table below.

Emetogenic risk	Standard antiemetic treatment	CR rate of Standard antiemetic treatment
High emetogenicity<br>(CDDP, dacarbazine etc.)	APR+$5HT_3$-RA+DEX	Acute phase 92%<br>Delayed phase 59–67%<br>Overall phase 59–66%
Moderate emetogenicity<br>(Irinotecan, oxaliplatin etc.)	5-HT_3-RA+DEX	Acute phase 75.3%<br>Delayed phase 56.8%<br>Overall phase 51.5%
Mild emetogenicity<br>(Mitomycin, mitoxantrone etc.)	DEX	No relevant study
Minimal emetogenicity<br>(Cetuximab, trastuzumab etc.)	Not required	No relevant study

* $5HT_3$-RA: $5HT_3$ receptor antagonist

The CR rate of standard antiemetic therapy (APR + 5-HT_3-RA + DEX) in the context of HEC is 92% in the acute phase, 59–67% in the delayed phase, and 59–66% in the overall phase; therefore, the control of nausea and vomiting in the delayed phase is not enough.

Predictive factors

Instances of nausea and vomiting are influenced by both treatment-related and patient-related factors; the former category is roughly subdivided into events that occur due to anticancer medication and those that accompany radiotherapy. Patient-related factors include age, sex, and drinking habits. Whereas female and/or young patients have an increased propensity to nausea and/or vomiting, the incidence of nausea and/or vomiting associated with CDDP is low in patients with a drinking habit[3,5-7].

Rationale for selection of the subject population

CDDP was developed in the late 1970's and is used to treat various cancer types. It has potent emetic effects, and is classified as an HEC–regardless of the dose–by both the ASCO and ESMO/MASCC 2011 guidelines. According to the NCCN guidelines, a high dose (50 mg/m^2 or higher) of CDDP was classified as an HEC until 2012 and a low dose (less than 50 mg/m^2) was classified as moderate risk until 2012; however, the guidelines were modified in 2013 and CDDP is now classified as an HEC regardless of the dose used. As the standard antiemetic therapy for HECs, APR or fosaprepitant are used in combination, but this is only the case for patients being treated with CDDP at doses of 70 mg/m^2 or higher [8,9].

Since the subjects of a randomized phase II trial conducted at the multicenter centering on the National Cancer Center Hospital [35] (See rP-II Study, 2.3. 2) c) were patients with solid cancer receiving HEC regimens with CDDP of 50 mg/m^2 or more, we selected a similar group of subjects for this study.

1) Reasons for excluding AC and EC therapies as HECs

Anthracyclines and cyclophosphamide are used for patients with breast cancer and are classified as moderately emetogenic chemotherapies (MECs) when used as monotherapy. When both drugs are used in combination

(AC/EC therapy), however, they are classified as HEC. The results from a recent clinical trial[10] of antiemetic therapy during the delayed phase of AC therapy are different from those of other HEC clinical trials. Specifically, it has been reported that DEX can be omitted from the results of the Phase III trial comparing the presence or absence of concomitant use of DEX after day 2 in a trial not using APR. In contrast, use of DEX on the 2nd and 3rd days is reportedly effective [11]; these different results highlight that the benefits of DEX as an antiemetic remain unclear.

For this reason, patients treated with AC and EC therapies were excluded from the present study because DEX treatment on Days 2 and 4 is an essential component of standard antiemetic therapy.

2) Reasons for excluding diabetic patients receiving treatment with either insulin or oral hypoglycemic agents and patients whose HbA1C (NGSP) is 6.5% or higher and whose HbA1c (JDS) is 6.1% or higher at registration.

Severe adverse events (including deaths) such as hyperglycemia, diabetic ketoacidosis, diabetic coma can potentially be triggered by OLZ, as the drug has well-described hyperglycemic effects. In this study, therefore, patients with diabetes complications and patients with HbA1C (NGSP) > 6.5% or HbA1c (JDS) > 6.1% will also be excluded. This because DEX, which itself induces hyperglycemia, is also included in the antiemetic therapy of both groups, although the OLZ oral administration period is only 4 days long.

2.2. Standard treatment for subjects

History of treatment development leading to the current standard treatment

In the 1960's, phenothiazines, which are dopamine receptor antagonists, were used as antiemetic agents. However, they had low efficacy and their use was associated with adverse effects such as sedation and ataxia [2]. Accordingly, metoclopramide (Primperan®), which is capable of selectively blocking dopamine D_2 receptors at a lower dose, was developed and is now used an antiemetic in patients with CINV. Metoclopramide that blocks dopamine D_2 receptors also blocks $5HT_3$ receptor at high doses [13]. High-dose metoclopramide therapy was developed in the 1980's and became the first effective treatment for CDDP-induced vomiting. However, efficacy of this drug is poor due to dose-limiting effects that induce extrapyramidal symptoms.

In the 1980's, the antiemetic effect of corticosteroids began to draw attention. Although the mechanism by which they exert antiemetic activity has not been fully elucidated, their efficacy has been demonstrated in many trials[14], with dexamethasone (DEX) being used the most frequently. Indeed, the combination of high dose metoclopramide and DEX has an improved antiemetic effect compared to either agent alone [15-17].

In the 1990's, 5-HT_3 receptor antagonists ($5HT_3$-RAs) were developed and adopted into clinical practice. In contrast to metoclopramide, $5HT_3$-RAs are not associated with dose-limiting extrapyramidal symptoms. In combination with high-dose metoclopramide, the antiemetic effect of $5HT_3$-RAs is significantly enhanced [18-19]. Thus, it became clear that $5HT_3$-RAs play a central role in CINV, and that their effects are further enhanced by combination with corticosteroids. Indeed, combined use of $5HT_3$-RAs and DEX has emerged as a standard antiemetic therapy. In Japan, granisetron (GRA, Kaitril®), ondansetron (Zofran®), azasetron (Serotone®), ramosetron (Nazea®), tropisetron (Naboban®) and indisetron (Synceron®) were developed as first-generation $5HT_3$-RAs, whereas PALO is a second-generation $5HT_3$-RA. To date, the Pharmaceutical Affairs Division has approved seven $5HT_3$-RAs.

Recently, it has been revealed that administration of antineoplastic agents enhances the secretion of substance P in the solitary nucleus in the lateral reticular of the medulla oblongata; the subsequent binding to NK1 receptors in the vomiting center then induces vomiting. In response to this, the NK1 receptor antagonist APR was developed and was approved in Japan in 2009. To date, there have been three phase III trials comparing a two-drug ($5HT_3$-RA and DEX)-combination therapy and a three-drug combination-therapy including APR in patients treated with HEC [20-22]. The CR rate was significantly higher in the three-drug combination-treated group (63–73%) than in the two-drug combination therapy-treated group (43–52%). From these results, standard antiemetic treatment for patients receiving the current HEC is a combination therapy using three agents: NK1 receptor antagonist, $5HT_3$-RA, and DEX.

Standard treatment in this study

Three phase III trials targeting HEC, demonstrated that a three-drug antiemetic therapy that included APR significantly improved the CR rate compared with the two-drug combination of ondansetron (a first generation $5HT_3$-RA) and DEX[20-22].

First author	Antiemetic therapy	CR rate (Overall phase)
Hesketh	Ondansetron + DEX + APR/placebo	72.7% vs. 52.3%, $p < 0.001$
Poli-Bigelli	Ondansetron + DEX + APR/placebo	62.7% vs. 43.3%, $p < 0.001$
Chawla	Ondansetron + DEX + APR/placebo	71.0% vs. 43.7%, $p < 0.05$

PALO, a second-generation $5HT_3$-RA, has a long half-life of about 40 hours, and has an inhibitory effect on nausea and/or vomiting. This is particularly the case during the delayed phase, and the drug is in many cases used as a clinical alternative to the existing first-generation $5HT_3$-RA agents, granisetron (GRA), ondansetron, and azasetron. Before APR was approved in Japan, a phase III controlled study comparing the antiemetic effects of either GRA or PALO in combination with DEX was conducted in patients treated with HEC. CR rates in the group treated with PALO and DEX were 75.3% in the acute phase, 56.8% in the delayed phase and 51.5% in the overall phase, while those in the group treated with GRA and DEX were 73.3% in the acute phase, 44.5% in the delayed phase and 40.4% in the overall phase. The CR rates during the delayed phase and the overall phase were significantly higher in the PALO+DEX group than in the GRA+DEX group[23].

In Japan, the Japan Society of Clinical Oncology published guidelines for proper use of antiemetic drugs. According to the guidelines, it is recommended that three drugs (APR + $5HT_3$-RA + DEX) be used as the standard antiemetic therapy for HEC. The APR dose is 125 mg on Day 1 (80 mg on Days 2 and 3) for oral administration and 150 mg in cases where intravenous infusion is recommended, in combination with intravenous infusion of $5HT_3$-RA and intravenous infusion of DEX at 9.9 mg (12 mg for oral administration) on Day 1. The oral DEX dose is 8 mg on Days 2–4. A phase III trial (TRIPLE test) comparing the 1st generation (GRA) and the 2nd generation (PALO) of $5HT_3$-RA in APR + $5HT_3$-RA + DEX has been carried out. The CR rate in the overall phase, which was the primary endpoint, was 59% in the GRA-containing group and 66% in the PALO-containing group; superiority was not shown in the PALO-containing group (odds ratio 1.35, 95% CI 0.99–1.82, $p = 0.0539$). However, the CR rate in the delayed phase, which was the secondary endpoint, was 59% in the GRA-containing group and 67% in the PALO-containing group; the rate in the PALO-containing group was significantly higher (odds ratio 1.45, 95% CI 1.07–1.96, $p = 0.0142$) [9].

CR rate in TRIPLE Study

	APR+PALO+DEX N = 414	APR+GRA+DEX N = 413	Odds ratio (95%CI)	*P* value
Overall phase	66%	59%	1.35 (0.99, 1.82)	0.0539
Acute phase	92%	92%	1.00 (0.58, 1.71)	1.0000
Delayed phase	67%	59%	1.45 (1.07, 1.96)	0.0142

Following this result, it was stated in the Guidelines for proper use of antiemetic agents [2nd edition] (Published in October 2015) of the Japan Society of Clinical Oncology that a 2nd generation $5\text{-}HT_3$-RA is preferred in the context of a 3-drug combination of APR + $5\text{-}HT_3$-RA + DEX.

Based on the above, we selected the APR + PALO + DEX triple combination as the standard antiemetic therapy for this current study.

2.3. Rationale for setting the treatment plan

Drugs

OLZ is a thienobenzodiazepine-type atypical neuroleptic that is widely used world-wide as an acute-phase and maintenance treatment for schizophrenia. It is also used for acute-phase treatment (monotherapy or combination therapy with lithium or valproic acid) and maintenance treatment of the manic symptoms of bipolar disorder. In Japan, oral administration of OLZ is initiated at 5 to 10 mg once a day and followed by oral administration of 10 mg once a day as a maintenance dose for schizophrenia. To treat the manic symptoms of bipolar disorder the drug is administered orally at

10 mg once a day and at 5 mg once a day. The dose is increased to 10 mg once a day for improvement of depression associated with bipolar disorder. The major adverse effects induced by OLZ are somnolence and weight gain [24-25].

The antiemetic effect of OLZ was reported relatively recently. The drug is classified as a MARTA (multi-acting receptor-targeted antipsychotic); it has high affinity for dopamine D_2 type (D_2, D_3, D_4), serotonin 5-HT2a, 2b, 2c, 5-HT_6, α_1 adrenaline and histamine H_1 receptors at the approximately same concentration range, and it also binds to dopamine D_1 type (D_1, D_5), serotonin 5-HT_3 receptor, muscarinic (M_1, M_2, M_3, M_4, M_5) receptor with slightly lower affinity [26-28]. OLZ acts as an antagonist to these receptors, and it is particularly its antagonism of D_2, H_1, and 5-HT_3 receptors that is believed to underlie its antiemetic effect. Based on this pharmacology, OLZ has been used in several clinical trials to date.

In Japan, OLZ has not been approved for treatment of nausea and vomiting. However, the Japanese Society for Palliative Medicine and the Japanese Society of Gastroenterology submitted a letter of request for the development of OLZ as a medicine for gastrointestinal symptoms (nausea/vomiting) associated with administration of antineoplastic agent. This has been reviewed by the Evaluation Committee on Unapproved or Off-labeled Drugs with High Medical Needs. The Committee has approached Eli Lilly (Japan) to develop this potential clinical application. The Specialized Working Group of this Committee, which was held on May 18, 2016, revealed that Eli Lilly desire public knowledge-based applications as their corporate outlook.

Test treatment of this study

The test treatment of this study will be a combination therapy with the standard antiemetic therapy (APR + PALO + DEX) and OLZ at 5 mg.

1) Controlled studies on antiemetic treatment with OLZ in foreign countries

a) Phase III trial to examine the significance of OLZ as part of an antiemetic therapy for HEC

Navari *et al*. conducted a double-blinded, randomized phase III study comparing the antiemetic effect of OLZ + PALO + DEX to that of APR + PALO + DEX, which is a standard antiemetic therapy for HEC. This study enrolled 401 patients, and the CR rates during the acute phase, delayed phase and overall phase (the three primary endpoints) were 97%, 77%, and 77%, respectively, in the group treated with OLZ+PALO+DEX, and 87%, 73%, and 73% respectively, in the group treated with APR + PALO + DEX. No significant differences were observed. However, the proportions of patients not showing nausea in the acute phase, delayed phase and overall phase were 87%, 69%, and 69%, respectively, in the group treated with OLZ+PALO+DEX and 87%, 38%, and 38%, respectively, in the group treated with APR+PALO+DEX. The control of nausea in the delayed phase and the overall phase was better in the group treated with the OLZ-containing regimen [29].

b) Phase III trial to examine the significance of OLZ as a rescue therapeutic agent for breakthrough nausea and vomiting

A Phase III trial comparing OLZ (10 mg once) and metoclopramide was conducted as a rescue therapeutic agent for breakthrough nausea and vomiting with the standard antiemetic therapy APR + PALO + DEX for HEC. Primary endpoints were the incidence of vomiting events within 72 hours after administration of OLZ or metoclopramide. The incidence was 31% in the OLZ-treated group and 70% in the metoclopramide-treated group, and there were significantly fewer vomiting events in the OLZ-treated group [30].

Based on the results of the above two trials, both combined use of PALO+DEX and OLZ a standard antiemetic treatment in cases where APR is not used for HEC (OLZ 10 mg on Days 1–4, PALO 0.25 mg on Day 1, DEX 20 mg on Day 1) and administration of OLZ 10 mg for 3 days for breakthrough vomiting are listed as Category 2A in the NCCN Guidelines i[31].

c) Current status of treatment development of antiemetic therapy by OLZ

A Phase III trial (Alliance A 221301) was conducted to verify the significance of addition of OLZ 10 mg to the standard antiemetic therapy with APR + 5HT3-RA+DEX for HEC. This study was conducted with APR+5HT3-RA+DEX plus either placebo or OLZ to determine whether OLZ reduced the number of patients with nausea symptoms [32]. Indeed, OLZ significantly reduced the incidence of nausea (which was the primary endpoint), and improved the CR rate in all phases (which was the secondary endpoint). These data are shown in the Table below.

Proportion of patients not showing nausea and CR rate in each period in Alliance A221301

		Placebo group N = 188	OLZ group N = 192	*P* value
Patients without nausea (%)	Acute phase	45	74	< 0.001
	Delayed phase	25	42	0.001
	Overall phase	22	37	0.002
CR rate (%)	Acute phase	65	86	< 0.001
	Delayed phase	52	67	< 0.007
	Overall phase	41	64	< 0.001

2) Treatment development of OLZ for HEC in Japan

Overseas, the dose of OLZ for an antiemetic treatment is 10 mg; however, 5 mg is widely used in daily clinical practice in Japan [33,34].

A placebo controlled double blind comparative study of OLZ (starting dose 10 mg) for Japanese patients with manic or mixed episodes due to bipolar I type disorder has been performed. adverse events of which incidence was higher In the OLZ-treated groups, the incidence of adverse events including somnolence (20.0%), dizziness, dry mouth (14.3%), and weight gain were higher than in the placebo groups. Somnolence and dizziness are not life threatening, but can increase the risk of falling. Therefore, OLZ 5 mg is widely used for antiemetic therapy in Japan.

a) Phase II study of APR + PALO + DEX + OLZ at 5 mg for HEC

Kansai clinical oncology group (KCOG) conducted a Phase II trial evaluating the efficacy and safety of APR + PALO + DEX + OLZ (5 mg) in gynecologic cancer patients undergoing HEC. The CR rate in the overall phase (which was the primary endpoint) was 92.5% (95% CI 79.6–98.4%), indicating its efficacy (threshold CR rate 55%, expected CR rate 75%, one-sided significance level 5%, detection power 80%)[33].

In addition, the Division of Thoracic Oncology of the Shizuoka Cancer Center conducted a Phase II study to evaluate the efficacy and safety of APR + PALO + DEX + OLZ (5 mg) for lung cancer patients undergoing HEC. The CR rate in the overall phase (which was the primary endpoint) was 83% (95% CI 70–92%), again indicating its efficacy (threshold CR rate 65%, expected CR rate 85%, one-sided significance level 5%, detection power 80%).

b) Controlled study to verify the significance of OLZ at 5 mg for HEC or MEC

A double-blinded, randomized controlled trial was conducted in two facilities, Sapporo Medical University and Higashi Sapporo Hospital, to verify the significance of the addition of 5 mg of OLZ to APR + $5HT_3$-RA + DEX against HEC or MEC [34].

The TC rate (which was the primary endpoint) was significantly superior in the OLZ-treated group than in the placebo group. However, the reliability of the results has since been questioned, because the method of administration of $5HT_3$-RA used was not typical, the TC rate of the placebo group was only 23% in this report compared to 40-50% in other reports, and the study sample size was small.

TC rate and CR rate for HEC and MEC in patients treated with APR + $5HT_3$-RA + DEX + OLZ/placebo

		Placebo group N = 22	OLZ group N = 22	Odds ratio (95% CI)	*P* value
TC rate (%)	Acute phase	55	86	5.28 (1.20 - 23.17)	0.0045
	Delayed phase	23	64	5.95 (1.59 - 22.33)	0.014
	Overall phase	23	59	4.91 (1.32 - 18.21)	0.031
CR rate (%)	Acute phase	86	100	8.08 (0.39 - 166.4)	0.233
	Delayed phase	73	100	17.73 (0.93 - 337.5)	0.021
	Overall phase	68	100	21.77 (1.16 - 410.1)	0.009

c) Phase II study on addition of OLZ at 10 mg and at 5 mg to standard antiemetic treatment (APR + PALO + DEX) for HEC

A randomized Phase II trial was conducted to compare the efficacy and safety of OLZ at 10 mg described in the NCCN guidelines and OLZ at 5 mg, the dose commonly used in Japan. Six facilities with a total of 153 patients were enrolled in this study, and the patients were randomly allocated by the Secretariat in the National Cancer Center Hospital. For the test drugs, OLZ tablets were crushed and mixed with lactose as the excipient so that the total amount was equal for both the 10 mg and 5 mg OLZ groups. For blinding, an unblinded pharmacist preparing study drugs was independently assigned in each facility to ensure that patients, physicians in charge, and pharmacists (clinical trial pharmacists) who played the role of clinical research coordinator did not know the allocation information. The primary endpoint was the CR rate in the delayed phase. The threshold of CR rate in the delayed phase of APR + PALO + DEX, which is the standard antiemetic therapy, was assumed to be 65%; an additional effect of 10% was considered to be the clinically minimum difference using OLZ concomitantly; and then the expected value was set to 75% (one-sided α = 0.5%, detection power = 75%). First, hypothesis tests were performed on the threshold of CR rate in the delayed phase of the 10 mg OLZ group. When the threshold was rejected, a closed testing procedure to perform the hypothesis test on the threshold of 5 mg OLZ was applied. In addition, when a significant difference was observed in both groups, it was decided which of 10 mg and 5 mg was more promising taking into account the incidence of adverse events. As a result, the CR rate during the delayed phase in the 10 mg OLZ group was 77.6% (80% CI: 70.3–83.8%) and the CR rate during the delayed phase in the 5 mg OLZ group was 85.7% (80% CI 79.2–90.7%); the threshold of 65% was rejected. On the other hand, drowsiness, which was an adverse event, was higher (53.3%) in the 10 mg OLZ group than the incidence (45.5%) in the 5 mg OLZ group.

Based on the above data, the 5 mg dose OLZ was selected for use with the standard antiemetic therapy APR + PALO + DEX in this current study.

Summary of risk-benefit balances of the standard treatment and study treatment

This is a study to examine whether 5 mg OLZ would improve the standard antiemetic therapy APR + PALO + DEX; the study will be designed as a superiority trial. Risks associated with the OLZ administration include weight gain, somnolence, malaise, and increased appetite; however, the frequencies of serious adverse events are low, and the administration period in this study is as short as 4 days. As for the medical cost, the price of OLZ is very low, at 258.50 yen/5 mg tablet and 1,034 yen/per 4 days. With regard to the benefits of OLZ administration, the drug has pharmacological properties different from any of the other three drugs used in the standard antiemetic therapy. Furthermore, OLZ is an antagonist of a variety of receptors such as serotonin, dopamine, muscarine, histamine that are thought to be involved in CINV; therefore, an additional antiemetic effect can be expected. This added benefit is considered to outweigh the risk of adverse events if an additional effect of 10% or more is obtained compared to placebo in the complete vomit control rate (CR rate) within 120 hours (delayed phase) from 24 hours after the initiation of CDDP administration.

After-treatment

Treatment after the "completion of protocol treatment" will not be stipulated, since the duration of the study is a maximum of 120 hours after CDDP administration, and therefore terminates at or before the end of protocol treatment.

2.4. Study design

Rationale for setting endpoints

The primary endpoint of this study is the CR rate in the delayed phase. Nausea and vomiting associated with chemotherapy are evaluated by dividing into two temporally distinct phases following chemotherapy: within 24 hours (acute phase) and from 24 hours onwards (delayed phase). The CR rate in the standard antiemetic therapy (APR + PALO + DEX) is 92% during the acute phase, 67% during the delayed phase, and 66% in the overall phase [9]. The low CR rate in the delayed phase requires further improvement, and it has therefore been selected as the primary endpoint in a number of phase III trials[36,37]. Thus, the CR rate in the delayed phase will be taken as the primary endpoint in this study.

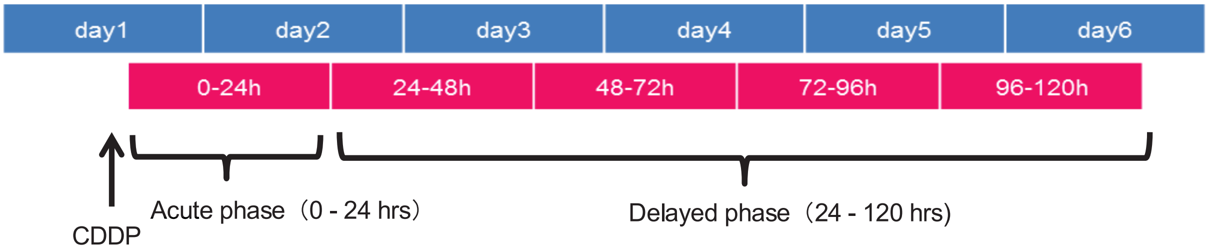

Clinical hypothesis and the rationale for setting the number of enrollments

The main hypothesis of this study is that OLZ will improve the CR rate of standard antiemetic care in the delayed phase. If this hypothesis is proven to be correct, we will judge that a combination therapy containing OLZ is a more useful antiemetic therapy.

In this study, we will assume that the CR rate during the delayed phase of the standard treatment group is 65%. We will conclude that OLZ is beneficial if we observe an improvement of 10% or more over this value. When setting the one-sided significance level at 5%, and the detection power is 80% under the above assumption, we calculate that the necessary sample size is 329 cases per group and 658 cases in total. Accordingly, we plan to enroll 690 patients in the 2-year registration period, considering that up to 5% of these individuals may be ineligible or unevaluable.

Estimated enrollments

We expect the enrollment of approximately 350 patients per year and completion of the enrollment in two years. In the rPII study that was conducted prior to this study and planned to complete patient accumulation in 2 years, 153 patients were enrolled in 9 months among 6 facilities (actually 9 facilities participated). Furthermore, in the TRIPLE study, which was the historical control of the rPII trial, 842 patients were enrolled within 11 months through 20 institutions (actually 25 facilities participated). Based on these actual achievements, we consider it feasible to enroll the target number of patients within 2 years.

Rationale for setting of allocation adjustment factors

1) Age (55 years old and over vs. less than 55 years old)

In a previous study of metoclopramide in patients receiving CDDP at 50 mg/m^2 or higher[38], the percentage of patients not showing vomiting was 43.4% in patients younger than 50 years old and 73.9% in patients over 65 years old; this indicates that suppression of vomiting was less effective in younger patients. In Japan, a multivariate analysis of phase II and phase III trials examining the effect of PALO in patients naive to CDDP and AC/EC therapy was run [39]. The analysis revealed that the odds ratio of nausea during the acute phase for patients under 50 years compared to those over 65 years was 2.55 (95 % CI 1.98–3.29) .

2) Gender

Three overseas studies on antiemetic treatment centered on metoclopramide in patients treated with CDDP reported that the incidence of vomiting was higher in females than in males[6,38,40]. In Japan, a multivariate analysis of phase II and phase III trials examining the effect of PALO in patients naïve to CDDP and AC/EC therapy has reported[39]. The odds ratios of nausea during acute and delayed phases in females were 2.80 (95% CI 2.01–3.90) and 1.59 (95% CI 1.10–2.31), respectively, compared to those in males.

3) Dose of CDDP (70 mg/m^2 or higher vs. less than 70 mg/m^2)

Currently, CDDP is regarded as HEC regardless of dose in various guidelines. In the past, however, CDDP at 50 mg/m^2 or higher was considered as HEC. In addition, in clinical trials examining the effect of APR on HEC, a criterion of patients receiving CDDP at 70 mg/m^2 or higher doses was used to define eligibility [20-22]. CDDP at 70 mg/m^2 was also set as an allocation adjustment factor in both the TRIPLE study and the Olanzapine rPII study.

2.5. Summary of the expected benefits and disadvantages associated with participating in the trial

Expected Benefits

When assigned to Group B in this study, there is a benefit that a drug (OLZ) which is not approved under the Pharmaceutical Affairs Law and not covered by health insurance are provided free of charge. However, this study will clarify whether Group B is provided with superior treatment compared to group A, and so the potential benefit of free OLZ cannot be determined at the present time. In addition, the study will not provide expenses for medical care other than medication cost of OLZ, hospitalization expenses and treatment for adverse events due to chemotherapy. Rather the patient's health insurance is applied, and the patient is responsible for self-payment. Therefore, there is no special economic benefit from participation in this study.

Expected risks and disadvantages

The standard antiemetic therapy given to patients in Group A is covered in the regular manner by health insurance, and it does not pose a special danger or disadvantage compared to daily medical treatment. The dose of OLZ used in Group B is 5 mg (orally administered once a day), which is within the range used for improving the manic symptoms and depressive symptoms in schizophrenia and bipolar disorder. Previously reported adverse effects in clinical trials that evaluated OLZ include weight gain, somnolence, malaise, and appetite stimulation. In this study there is a possibility of somnolence and accompanying falls due to the administration of OLZ. However, OLZ will only be administered for 4 days, which is in contrast to the much longer chronic dosing of the drug in the psychiatric field. In addition, the Tmax (about 5 hours) of OLZ will be achieved while the patient is sleeping if the OLZ is taken after dinner. Therefore, we believe that the risk of somnolence and falling during the day will be reduced.

2.6. Significance of this study

While OLZ is widely used as an antiemetic agent, it is only listed in the NCCN guidelines. Although MASCC describes OLZ as up to date, the text has not been revised, as the OLZ dose is reported as 10 mg. Currently, the use of OLZ for treatment of CINV is off-label in Japan. The "Evaluation Committee on Unapproved or Off-labeled Drugs with High Medical Needs" of the Ministry of Health, Labour and Welfare recommends "to administer OLZ at 5 mg or at 10 mg against gastrointestinal symptoms (nausea/vomiting) associated with antineoplastic agent administration" as Request No.: III-①-22.2. In this letter of request, there is a statement that "It is desirable that double-blinded II/III phase studies to examine additional effects of OLZ on existing triple therapy will be conducted." In the previous rPII study, the efficacy of OLZ at both 5 mg and 10 mg was deemed equivalent, but there was concern that 10 mg OLZ would induce drowsiness.

We feel that our study is required in order to provide new guidelines for the use of OLZ as an antiemetic in Japan. In particular, this would allow us to determine whether the 5 mg dose is sufficient for maximal clinical benefit, and confirm whether this dose is also associated with the best quality of life indices, as previously reported. This is important since the adverse effects of drowsiness and subsequent falls could then be mitigated. On the other hand, if we find 10 mg is required for maximal effectiveness we would be able to inform Japanese clinicians of the risks associated with this dose.

2.7. Additional study

No additional studies have been planned at the time of protocol preparation.

3. Criteria and definitions used in this study

Emetic risk will be classified according to the Guidelines for proper use of antiemetic agents edited by the Japan Society of Clinical Oncology in this study.

3.1. Definition of "High emetic risk"

Classification	Drug regimen
High emetic risk (Incidence of emesis> 90%)	AC therapy: Doxorubicin + cyclophosphamide<br>EC therapy: Epirubicin + cyclophosphamide<br>Cyclophosphamide (≥ 1,500 mg/m^2)<br>Cisplatin<br>Streptozocin<br>Dacarbazine<br>Carmustine (> 250 mg/m^2) (Not approved in Japan)<br>Mechlorethamine (Not approved in Japan)

4. Patient selection criteria

Patients fulfilling all of the following eligibility criteria and not meeting any of the exclusion criteria shall be considered eligible for the registration.

4.1. Eligibility criteria (Inclusion criteria)

(1) Patients with malignant tumors excluding hematopoietic malignant tumors
(2) Patients scheduled to receive CDDP (CDDP ≥ 50 mg/m^2) for the first time. However, divided doses are ineligible.
(3) Patients who are over 20 years old or younger than 75 years old.
(4) Patients whose ECOG performance status (PS) is 0 to 2.
(5) Patients with neither symptomatic brain metastasis nor carcinomatous meningitis.
(6) Patients using none of the following concomitant medications within 48 hours before registration.

NK_1 receptor antagonists	Aprepitant
$5HT_3$ receptor antagonists	Granisetron, ondansetron, azasetron, tropisetron, ramosetron, indisetron, palonosetron
Corticosteroids	All (Excluding external preparations)
Anti-dopaminergic drugs	Metoclopramide, domperidone
Phenothiazine tranquilizers	Prochlorperazine, perphenazine
Antihistaminics	Chlorpheniramine maleate, diphenhydramine, dimenhydrinate, hydroxyzine, promethazine
Benzodiazepines (Including thienodiazepines)	All*
Barbiturates	Primidone, phenobal, phenobarbital
Others	Haloperidol, droperidol, scopolamine, pimozide, clarithromycin, ketoconazole, itraconazole, rifampicin, phenytoin, carbamazepine, fluvoxamine, ciprofloxacin, Suvorexant, ramelteon

* Concomitant use of benzodiazepines (including etizolam, clotiazepam etc.) is prohibited. However, administration up to once per day of the following hypnotics aimed at improving insomnia is permitted.

- Ultrashort-acting hypnotics (triazolam, zopiclone, eszopiclone, zolpidem)
- Short-acting hypnotics (brotizolam, lormetazepam, rilmazafone)

However, since there is a possibility of drowsiness due to oral administration of the test drug, the above hypnotics should not be used concomitantly on the first day of administration of the test drug in principle. When using the above hypnotics in combination, the severity of drowsiness due to oral administration of the test drug should be evaluated in advance and the drug should be used carefully if light headedness at nocturnal awaking is experienced. (It is advisable to consult with clinical trial pharmacists about concomitant use of the above drugs).

(7) The latest laboratory data supplied no more than 7 days before the registration (same day as one week before the registration date is acceptable) satisfy all of the followings:
① T-Bil ≤ 2.0 mg/dL

② AST ≤ 100 U/L
③ ALT ≤ 100 U/L

(8) The consent about participation has been obtained from the patient.

4.2. Exclusion criteria

(1) Patients who have a history of allergic reactions to drugs used in this study and their analogues.
(2) Patients with nausea and vomiting requiring treatment with antiemetic agents at registration.
(3) Patients who started narcotic formulations (potent opioid analgesics) within 48 hours prior to the enrolment.
(4) Patients with history of any one or more of unstable angina, myocardial infarction, cerebral hemorrhage, cerebral infarction, or active gastroduodenal ulcer within 6 months before the registration.
(5) Patients with convulsive disorder requiring anticonvulsant treatment.
(6) Patients with ascites retention requiring therapeutic puncture.
(7) Patients with gastrointestinal obstruction, such as having gastro-pyloric constriction or intestinal obstruction.
(8) Females who are pregnant, nursing or may become pregnant, or patients who do not plan to use contraception.
(9) Patients who have mental illness or psychiatric symptoms that interfere with daily living and would therefore be considered difficult to manage during the study.
(10) Patients who received radiation therapy of the abdomen (below the diaphragm) or pelvis within 6 days before the registration, or patients who are scheduled to receive it within 6 days of CDDP administration. On the other hand, patients who have undergone radiation therapy (lumbar vertebra, etc.) limited to bone parts for bone metastasis, or chemoradiotherapy in regions above the diaphragm, chest region and cervical region, are not excluded.
(11) Patients with diabetes who are receiving treatment with either insulin and/or oral hypoglycemic agents and patients with HbA1c (NGSP) of 6.5% or more or HbA1c (JDS) of 6.1% or more at the time of registration.
Patients who have the possibility of dose reduction / termination of dexamethasone depending on the result of blood glucose determination performed in advance are also excluded.
(12) Patients who cannot be hospitalized up to 120 hours (Day 6) after the initiation of CDDP administration.
(13) Patients with smoking habits at the time of registration.
(14) Other patients who are deemed inappropriate for participation in this study.

5. Registration and allocation

5.1. Registration procedures

Participants will be registered from a Web Entry System after confirming that the target patient meets all eligible criteria and does not fall under any of the exclusion criteria. For Web registration, a personal account and password for the Web Entry System will be required. If these are unknown, contact the Data Center.

Patient registration Web Entry System
URL: Under construction
(Web entry is available for registration 24 hours a day.)

Inquiries about patient registration and the Web Entry System:
Data Center
Data Management Office, Clinical Research Support Division, National Cancer Center Hospital
Phone: 03-3547-6600
Weekday: 9:00 to17:00 (The Data Center will be closed on Holidays, Saturdays and Sundays, New Year's holidays)
E-mail: NCCH_CDM@ml.res.ncc.go.jp

Inquiries about the patient selection criteria:
Principal Investigator: Masakazu Abe, M.D.
Department of Gynecologic Oncology, Shizuoka Cancer Center
1007 Shimonagakubo, Nagaizumi-cho, Sunto-gun, Shizuoka Prefecture 411-8777, Japan
Phone: +81-55-989-5222 Ext. 6202

Fax: +81-55-989-5551
E-mail: ma.abe@scchr.jp

Co-principal Investigator: Hironobu Hashimoto, Assistant Director
Department of Pharmacy, National Cancer Center Hospital
Tsukiji 5-1-1, Chuo-ku, Tokyo, 104-0045, Japan
Phone: +81-3-3542-2511 Ext. 3531
Fax: +81-3-3248-0730
E-mail: hhashimo@ncc.go.jp

5.2. Notes on registration

1) Registration after the initiation of the protocol treatment is unacceptable without exception.
2) Registration is done by accessing the URL for "Patient registration" described in section 5.1.
3) Since eligibility will be confirmed on the registration screen, there is no need to send the registration eligibility questionnaire to the Data Center by mail or fax.
4) When the input data is insufficient, the registration is not accepted until all conditions are satisfied.
5) After the eligibility is confirmed on the registration screen, the registration is completed and a registration number will be issued.
6) The allocation result can be confirmed only by the person in charge of allocation information in each institution and the Study Secretariat on the WEB Entry System.
7) The registration of patients is not canceled (deleted from the database) unless there is a withdrawal of agreement including refusal of research use of data. In cases of duplicated registration, the first registration information (registration number) will be adopted.
8) When an incorrect registration or duplicated registration is found, contact the Data Center immediately.

5.3. Random allocation and stratification factors

Subjects will be randomly allocated into treatment groups at the Data Center upon registration. Upon the random allocation, a minimization method using factors including [1] age (55 years old and over vs. below 55 years old), [2] gender, and [3] CDDP dose (70 mg/m^2 or more vs. less than 70 mg/m^2) will be used as adjustment factors to avoid serious deviation from these categories within the treatment groups. Detailed procedures of the random allocation will not be provided to investigators at participating institutions.

5.4. Blinding

Method of blinding

This study will be blinded so that patients, physicians in charge, and medical staff (persons in charge of clinical trials) who play the role of clinical research coordinators (CRCs) do not know the allocation information. The designations of the staff and their roles necessary for that purpose will be defined as follows.

(1) The person in charge of allocation information
Unblinded staff that are allowed to receive the allocation information.
The person in charge of allocation information will be selected in advance and will receive the information from the Data Center (Web Entry System). Pharmacists are the first choice as persons eligible to receive the information. However, other qualified individuals include staff members of the clinical trial management office, or assistants.

(2) Dispensing pharmacist
Unblinded staff that are allowed to receive the allocation information.
Dispensing pharmacists will be selected in advance, and will prepare the study drugs in accordance with the allocation information. The dispensing pharmacists can also serve concurrently as the person in charge of allocation information, although there must be at least two individuals that cover the person in charge of allocation information and dispensing pharmacist roles

(3) Persons in charge of the clinical trial
Blinded staff who do not know the allocation information.
Persons in charge of the clinical trial will play the role of CRCs and work on informed consent (IC) assistance, confirmation of registration eligibility, patient registration and others in this study. They strive to facilitate this study, including establishing an implementation system in the hospital and exchanging information with the Study Secretariat. The role is recommended primarily for pharmacists, but other professionals will be considered.
Persons in charge of the clinical trial may also be engaged in dispensing tasks other than those within the remit of this study. In this case, the person in charge of dispensing shall facilitate the visual discrimination of the prescription for the subject of the study, and strive to ensure the blinding of the clinical trial representative.

(4) Physicians in charge
Blinded staff who do not know the allocation information.
Physicians in charge will be responsible for the prescription of the study drugs, and shall work closely with the person in charge of the clinical trial to ensure the study proceeds smoothly.

Scheme of blinding

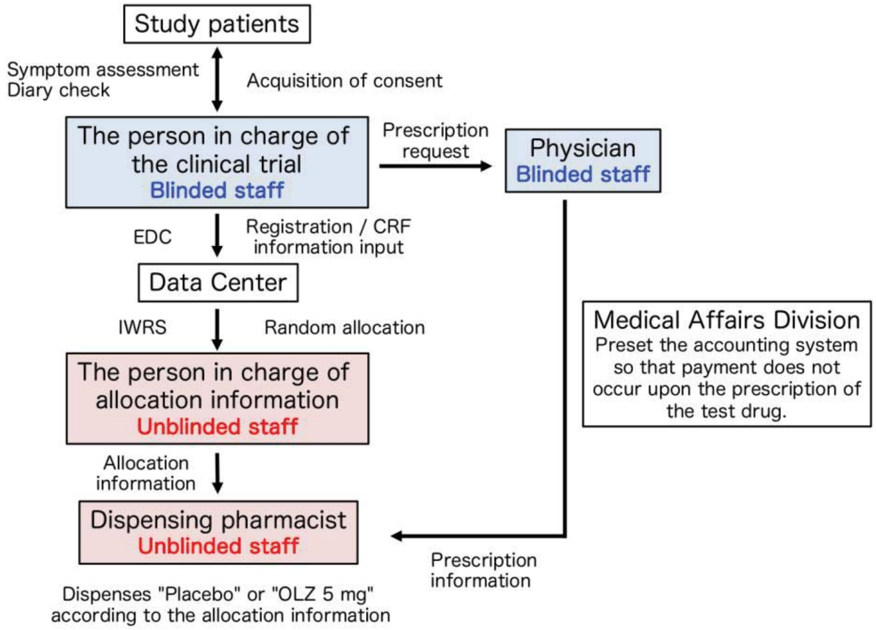

Unblinding in case of emergency (key open)

As a general rule, the information on the study will not be unblinded until all data is fixed. However, there may be instances when it is considered necessary that the contents of the study drug are identified in order to ensure the safety of the patient for reasons such as occurrence of serious adverse events. In such cases, the Study Representative and the Research Secretariat will discuss whether unblinding should occur. If it is determined that unblinding is necessary, the principal investigator/coordinators of participating institutions will be notified, and the Research Secretariat will disclose the contents of the study medicine.

5.5. Preparation of study drugs

Study drugs will be formulated at the Research Secretariat as follows, and will be distributed to each institution:

Group A: Placebo group

Placebo capsules will be made by filling D-size capsules (Capsugel, DBcaps) with 0.25 g of lactose.
Two capsules will be used for one dose. The capsules are administered once daily after dinner from Day 1 to Day 4 of CDDP administration.

Group B: OLZ 5 mg group

Capsules containing OLZ will be prepared by filling D-size capsules with 0.25 g of Zyprexa® fine grain 1%.

Two capsules are used for one dose. The capsules are administered once daily after dinner from Day 1 to Day 4 of CDDP administration.

6. Treatment plan and treatment change criteria

6.1. Protocol treatment

This study will be a randomized, double-blinded study. Registration and allocation will be performed using the Web Entry System. This study will maintain blindness by placing pharmacists who independently prepare the study drugs based on the blinding information (dispensing pharmacists) so that patients, physicians in charge, and staff who play the role of clinical research coordinators (CRCs) (persons in charge of clinical trial) cannot know the allocation information.

Protocol treatment will be started within 8 days after the registration. If the treatment is started after the 9th day for any reason, this reason should be entered for the treatment course record. If the treatment cannot be started, the underlying reasons should be described in detail in the "Treatment Termination Report" as "Cancellation of the protocol treatment".

It will be at the discretion of the attending physician to decide whether to start or stop the protocol treatment when eligibility criteria are at risk of being unfulfilled due to a worsening of laboratory test values or the like after registration.

Antiemetic therapy

The following antiemetic therapy is performed for 4 days from the start date of CDDP administration (Day 1).

Control arm (Group A): APR+PALO+DEX+Placebo

< In cases of oral administration of APR (When Emend® capsules are used) >

Group	Therapeutic regimen				
	Drug	Day 1	Day 2	Day 3	Day 4
Group A (Placebo group)	Placebo (p.o.)	Placebo	Placebo	Placebo	Placebo
	APR (p.o.)	125 mg	80 mg	80 mg	-
	PALO (i.v.)	0.75 mg	-	-	-
	DEX (i.v. or p.o.)	12 mg	8 mg	8 mg	8 mg

< In cases of intravenous administration of APR (When Proemend® for intravenous infusion is used) >

Group	Therapeutic regimen				
	Drug	Day 1	Day 2	Day 3	Day 4
Group A (Placebo group)	Placebo (p.o.)	Placebo	Placebo	Placebo	Placebo
	APR (i.v.)	150 mg	-	-	-
	PALO (i.v.)	0.75 mg	-	-	-
	DEX (i.v. or p.o.)	12 mg	8 mg	16 mg	16 mg

Experimental arm (Group B): APR+PALO+DEX+OLZ

< In cases of oral administration of APR (When Emend® capsule is used) >

Group	Therapeutic regimen				
	Drug	Day 1	Day 2	Day 3	Day 4
Group B (OLZ group)	OLZ (p.o.)	5 mg	5 mg	5 mg	5 mg
	APR (p.o.)	125 mg	80 mg	80 mg	-
	PALO (i.v.)	0.75 mg	-	-	-
	DEX (i.v. or p.o.)	12 mg	8 mg	8 mg	8 mg

< In cases of intravenous administration of APR (When Proemend® for intravenous infusion is used) >

Group	Therapeutic regimen				
	Drug	Day 1	Day 2	Day 3	Day 4
Group B (OLZ group)	OLZ (p.o.)	5 mg	5 mg	5 mg	5 mg
	APR (i.v.)	150 mg	-	-	-
	PALO (i.v.)	0.75 mg	-	-	-
	DEX (i.v. or p.o.)	12 mg	8 mg	16 mg	16 mg

•OLZ/Placebo: 5 mg of OLZ or placebo will be administered on Day 1 to Day 4 of CDDP administration once **daily after dinner**.

• APR: < In cases of oral administration > "APR capsule 125 mg" will be administered on Day 1 at least 60 minutes before CDDP administration. "APR capsule 80 mg" will be administered on Days 2 and 3 after breakfast or until noon.

< In cases of intravenous administration > "APR for intravenous infusion" is administered at least 60 minutes prior to CDDP administration.

• PALO: PALO will be administered at least 30 minutes before CDDP administration.

•DEX: Prescribed dose of DEX will be administered at least 30 minutes prior to CDDP administration. Oral intake will be acceptable instead of intravenous infusion.

Dosage regimen of DEX will be as follows:

< In cases where APR is orally administered > Day 1: 12 mg, Days 2–4: 8 mg

< In cases where APR is intravenously administered> Day 1: 12 mg, Day 2: 8 mg, Day 3–4: 16 mg

※ DEX will be taken once a day after breakfast or after lunch or taken twice a day after breakfast and after lunch.

※ Patients who continue to be treated with low risk anticancer agents until Day 5 (esophageal cancer: FP therapy, etc.) will be allowed to continue DEX administration up to Day 5 if so prescribed in the regimen in advance. (When APR is orally administered, the dose of intravenous DEX will be 6.6 mg (8 mg for oral DEX); when APR is administered intravenously, the dose of infused DEX will be 13.2 mg (16 mg in the case of oral DEX).

6.2. Unacceptable combination therapy

1) Any of the following agents other than APR and PALO, (two of the drugs used in the protocol treatment) shall not be administered:
   ① NK_1 receptor antagonists
   ② 5-HT_3 receptor antagonists
2) Adrenaline shall not be used within 48 hours of the start to 120 hours after CDDP administration.
3) The following drugs shall not be administered within 24 hours of the start to 120 hours after CDDP administration:
   ① Selective Serotonin Reuptake Inhibitors (SSRIs), and serotonin and norepinephrine reuptake inhibitors (SNRIs)
   ② Serotonin-dopamine antagonists (SDAs) and multi-acting receptor targeted antipsychotics (MARTAs), with the exception of OLZ in the protocol treatment.
   ③ Substrates, inhibitors, and inducers of CYP3A4 (The following drugs have possible interactions with APR):
   ・ Substrates and inhibitors of CYP3A4
   Pimozide, clarithromycin, ketoconazole, itraconazole
   ・ CYP3A4 inducers
   Barbiturates (primidone, phenobal, phenobarbital), rifampicin, phenytoin, carbamazepine
   ④ Inhibitors of CYP1A2 (The following drugs have possible interactions with OLZ):
   ・ Fluvoxamine, ciprofloxacin
   ・ When prophylactic administration of quinolone antibiotics is initiated for the purpose of preventing bacterial infection during the protocol treatment, consider the use of levofloxacin, as this drug does not inhibit CYP1A2.
   ⑤ New use of narcotic formulation (potent opioid analgesics) (<u>Dose increase and rescue treatment will be</u>

acceptable.)

⑥ The drugs listed in Table 6.2 are prohibited. However, this is not the case when administering drugs in accordance with 6.1.

Table 6.2 Unacceptable combination drugs during the protocol treatment

NK_1 receptor antagonist	Aprepitant
$5HT_3$ receptor antagonist	Granisetron, ondansetron, azasetron, tropisetron, ramosetron, indisetron, palonosetron
Corticosteroid	All (except topical preparations)
Anti-dopamine drug	Metoclopramide, domperidone
Phenothiazine tranquilizer	Prochlorperazine, perphenazine
Antihistamine	Chlorpheniramine maleate, diphenhydramine, dimenhydrinate, hydroxyzine, promethazine
Benzodiazepine (Including thienodiazepines)	All*
Barbiturate	Primidone, phenobal, phenobarbital
Others	Haloperidol, droperidol, scopolamine, pimozide, clarithromycin, ketoconazole, itraconazole, rifampicin, phenytoin, carbamazepine, fluvoxamine, ciprofloxacin, Suvorexant, ramelteon

* Concomitant use of benzodiazepines (including etizolam, clotiazepam, etc.) will be prohibited. However, administration up to once per day of the following hypnotics aimed at improving insomnia is permitted.

- Ultrashort-acting hypnotics (triazolam, zopiclone, eszopiclone, zolpidem)
- Short-acting hypnotics (brotizolam, lormetazepam, rilmazafone)

However, there is a possibility of drowsiness due to oral administration of the test drug, and in principle the above hypnotics shall not be used concomitantly on the first day of administration of the study drugs.
When using the above hypnotics in combination, evaluate the severity of drowsiness due to oral administration of the study drugs in advance and monitor for light-headedness upon nocturnal awaking, etc. (It is desirable to consult with clinical trial pharmacists prior to approving concomitant use)

4) Radiation therapy to the abdomen (to be below the diaphragm) or the pelvis for 12 days from 120 hours before CDDP administration to 120 hours after CDDP administration

6.3. Discontinuation and completion criteria of the protocol treatment

Definition of protocol treatment completion

The protocol treatment is deemed complete upon final observation, which is 120 hours after initiation of CDDP administration.

Discontinuation criteria of the protocol treatment

1) When it is determined that additional administration of OLZ is necessary *
※ The use of **OLZ** for rescue treatment in addition to the **study drug** (placebo or **OLZ**) will not be allowed during the protocol treatment; however, when it is judged that additional administration of OLZ is necessary for treatment, discontinuation of the protocol treatment will be permitted, and OLZ can be administered.
2) When the protocol treatment cannot be continued due to adverse events.
3) When the patient has requested to discontinue the protocol treatment due to the reason that the relation with adverse event cannot be denied.
4) When the patient has requested to discontinue the protocol treatment due to the reason that the relation with adverse event can be denied (Including cases of patient rejection after the registration before starting protocol treatment).
5) Exacerbation of the original disease after registration and/or death (Including cases where protocol treatment could

not be started due to exacerbation of nausea and/or vomiting after registration but before treatment starts)

6) When a protocol violation is found (for example, when any of drugs listed in Table 6.2 Unacceptable combination drugs during the protocol treatment" would be used.)

7) When a change in pathological diagnosis leads to an altered treatment regimen, thereby causing ineligibility.

The protocol treatment withdrawal date shall be the day on which the attending physician deemed the patient ineligible, if ineligibility is found after the registration. Otherwise, the date is determined by the day on which the attending physician decides to stop the protocol treatment.

6.4. After-treatment

Treatment after the "completion of protocol treatment" is not stipulated. This study is a trial to examine the additional effect of OLZ on antiemetic therapy for HEC between 24 and 120 hours after CDDP administration; therefore, treatment later than 120 hours after CDDP administration does not affect the results. If nausea and/or vomiting are observed after the completion/discontinuation of the protocol treatment, antiemetic therapy due to adverse events and concordant with the patient's wishes is performed according to the antiemetic guidelines.

7. Expected adverse effects

For expected adverse effects to individual drugs, refer to the latest edition of "Package Insert Information on Ethical Drugs: http://www.info.pmda.go.jp/psearch/html/menu_tenpu_kensaku.html" on the website of the Pharmaceuticals and Medical Devices Agency.

7.1. Adverse effects by treatment group

Expected adverse effects in the control arm (Group A)

Adverse events of Grade 3 observed in the study treatment group (APR + PALO + DEX) in the TRIPLE study shown in Section 2.2 were constipation (1.7%) and hiccup (0.7%). The occurrence rates of adverse events in this study are shown by grade below:

N = 421 (%)	Grade 1	Grade 2	Grade 3
Constipation	117 (28.2)	93 (22.4)	7 (1.7)
Increased ALT	41 (9.9)	2 (0.5)	0 (0)
Hiccup	29 (7.0)	25 (6.0)	3 (0.7)
Hyperlipidemia	12 (2.9)	7 (1.7)	0 (0)
Increased AST	12 (2.9)	0 (0)	0 (0)
Hyponatremia	9 (2.2)	0 (0)	0 (0)
Hypoalbuminemia	4 (1.0)	2 (0.5)	0 (0)

Expected adverse effects in the experimental arm (Group B)

No adverse events of Grade 3 or higher were observed in the KCOG trial (described in Section 2.3.2) during development of treatments for HEC in Japan. The main adverse events were somnolence, constipation, and dry mouth.

In addition, Grade 3 adverse events observed in patients treated with 5 mg or 10 mg of OLZ in randomized Phase II trials examining the additional effect of OLZ when added to standard antiemetic therapy (APR + PALO + DEX) for HEC were 1 case of constipation (1.3%) and 1 case of hyponatremia (1.3%).

The incidences of adverse events in the two studies are shown by grade below:

	KCOG Study (n=40), n (%)		OLZ rPII 5 mg group (n=77), n (%)		
	Grade 1	Grade 2	Grade 1	Grade 2	Grade 3
Somnolence	33 (82.5)	0 (0)	34 (44.2)	1 (1.3)	0 (0)
Constipation	6 (5)	23 (57.5)	10 (13.0)	4 (5.2)	1 (1.3)
Increased ALT	-	-	8 (10.4)	0 (0)	0 (0)
Hiccup	0 (0)	0 (0)	3 (3.9)	0 (0)	0 (0)
Hyponatremia	-	-	3 (3.9)	0 (0)	1 (1.3)
Dry mouth	7 (17.5)	0 (0)	1 (1.3)	0 (0)	0 (0)
Hyperglycemia	-	-	4 (5.2)	0 (0)	0 (0)
Dizziness	2 (5)	0 (0)	-	-	-

7.2. Evaluation of adverse events/adverse effects

The Japanese translation of "NCI-Common Terminology Criteria for Adverse Events v4.0 (CTCAE v4.0)" (hereinafter referred to as "CTCAE v4.0-JCOG") is used for evaluation of adverse events/adverse effects.

An adverse event will ascribed to the closest grade according to the definitions of Grades 0 to 4.

Furthermore, if a specific treatment is described in the Grade, grading will be done based on its clinical need. For example, a patient with increased pleural effusion may reject indicated treatments such as oxygen inhalation or chest cavity drainage, regardless of the situation requiring them. In such a case, the grading will be done based on the recommended medical procedure that should be followed, and will not be based on what was actually done.

In case of treatment-related death, the causal adverse event is defined as "Grade 5" in the original NCI-CTCAE; however, in the record of this study, it will be defined as "Grade 4". In order for a causal relation between adverse event and death to be considered as treatment related death, a description should be provided in the Treatment Termination Report or the "Status at the time of death" column of a treatment completion report or a follow-up survey, and an urgent report should be filed. (Whether such an occurrence should be re-labeled as “Grade 5” will be determined via after-the-fact consideration of all information, including the Emergency report.) For adverse event items specified in "8.2. Observation, inspection and evaluation items to be performed during the protocol treatment", the Grade of an adverse event and its first onset date will be recorded in a relevant record (the ‘Therapeutic Course Record’). For other adverse events, their description, grade and initial onset date will be recorded in the free entry column of a Therapeutic Course Record form only when an adverse event of Grade 3 or higher is observed.

Be sure to transcribe the grade in a medical record.

8. Evaluation items, laboratory examinations, and evaluation schedule

8.1. Before treatment initiation

During this period, the attending physician or the person in charge of the clinical trial will obtain the patient's free will consent from the patient him/herself. After acquiring the consent document, observations, examinations and the evaluation shown in section 8.1 are performed. Observations and examinations other than surveys on a patient’s background will be carried out no later than 7 days before the registration; the patient's eligibility for this study is confirmed based on the results of these observations and examinations, and the registration procedure is then completed. If examination results conducted within the scope of routine medical treatment are available, these results can be incorporated into this study even if the examination was performed before the acquisition of consent.

The results of the observations, examinations and evaluations will be used as reference values before drug administration. This will facilitate the verification of drug-induced changes.

If additional observations, examinations and/or evaluations are carried out between the time of the registration and the administration of the study drugs, the latest results will be used as reference values. Administration of the study drugs shall be carried out within 8 days (including the day of registration) from the registration date.

Matters to be entered in a pre-treatment form

1) Patient background

Subject identification code (code arbitrarily set in each institution at the time of registration), registration

number (number given at the time of registration), date of consent acquisition, date of birth, age (age at registration), gender, height, weight, and cancer type

2) ECOG PS
3) Laboratory examinations
Hematological examinations: White blood cell count (WBC), hemoglobin (Hb), platelet count (PLT)
Biochemical examinations: Total bilirubin (T-Bil) aspartate aminotransferase (AST), alanine aminotransferase (ALT), γ-glutamyl transpeptidase (γ-GTP), albumin (ALB), serum creatinine (Scr), sodium (Na), potassium (K), blood glucose level (fasting or casual levels can be used)), and HbA1c*
* HbA1c tested within 28 days before the registration can be used.
4) Scheduled chemotherapy regimen (dose of cisplatin and concomitant anticancer drug (mg/m^2))
5) Scheduled combined radiation therapy

8.2. Protocol treatment period

The period of the protocol treatment is from the time of cisplatin administration on the day of the initiation of the study drug administration (Day 1) to 120 hours after the time of cisplatin administration (Day 6).

Blood collection for laboratory examination will be carried out on Day 6 in principle. However, this is not possible, blood collection on Days 5–8 is acceptable.

Patients should be hospitalized until at least 120 hours after the CDDP administration (Day 6).

Observations, examinations and evaluations to be continuously performed during the protocol treatment

1) Observations and evaluation items by the attending physician or the person in charge of the clinical trial (evaluated according to CTCAE v4.0 - JCOG)
① Constipation, hiccups, somnolence, insomnia, dizziness, dry mouth: Grade, causal relationship with the study drug
② Other adverse events: Grade, causal relationship with the study drugs

2) Items evaluated by patients (items described in the "Symptom Diary")
① Vomiting (including retching): frequency, date and time of the first onset
② Nausea: Evaluation by a 4-grade categorical scale)
③ Treatment with additional antiemetic agent: Number of additional treatments, date and time of the first additional treatment
④ Presence or absence of anorexia and its severity
⑤ Presence or absence of drowsiness and its severity
⑥ Presence or absence of disturbance of daily life due to drowsiness and its severity
⑦ Presence or absence of hindrance to sleep and its severity

Laboratory tests: Day 6 (Days 5 to 8 are also acceptable.)

The same items as those in section 8.1.3, with the exception of HbA1c.

After the completion of the protocol treatment (from the completion of protocol treatment to the day of discharge)

1) Evaluation of the level of patient satisfaction with the antiemetic drug (evaluation according to a 7-grade categorical scale)
2) Presence or absence of patient-related factors for nausea and vomiting (Whether the patient is prone to motion sickness, has a drinking habit that exceeds occasional drinking [*1]], or has experiences of hyperemesis [*2])
*1: 'Occasional drinking' is defined as "occasionally taking alcohol with meals or during social occasions, travel, parties etc."
*2: Experience of hyperemesis: Patients who have not been pregnant shall be report as "none" for this category. This category includes men.

When the protocol treatment discontinued

The date of discontinuation of the protocol treatment, and the reason for discontinuation will be described.

8.3. Study calendar

Examination, observation, evaluation item	Study period (From the day of registration to Day 6 or the day of discontinuation)									
	Before treatment		During protocol treatment							
	Within 7 days before registration	Registration day	Day 1	Day 2	Day 3	Day 4	Day 5	Day 6	Day 8	At the time of discontinuation
Registration		○								
Study drugs administration			○	○	○	○				
APR administration *1 (p.o.)			○	○	○					
APR administration *2 (i.v.)			○							
PALO administration			○							
DEX administration *3			○	○	○	○				
CDDP administration			○							
Patient background	○									
PS	○									
Laboratory examinations<br>• Hematology<br>• Biochemistry<br>• Blood glucose level	○						(○*4)	○*4	(○*4)	○
HbA1c*5	○									
Efficacy evaluation (Carried out during hospitalization)			←	—	—	—	—	—	→	○
Adverse event evaluation			←	—	—	—	—	—	→	○
Patient diary entry			←	—	—	—	—	—	→	○
Patient diary collection								○		○

*1 When APR is administered orally

*2 When APR is administered intravenously

*3 With regard to DEX administration, if the low-grade antineoplastic drug is continued until day 5 of the subject regimen (Esophageal cancer: FP therapy etc.), the administration of DEX as prescribed in the regimen will be allowed up to day 5.

*4 Laboratory examinations during the protocol treatment will be conducted once at Day 6 (but acceptable on Days 5–8).
*5 HbA1c will be measured within 28 days before the registration (measurements taken on the same day of registration are acceptable)

9. Data collection

9.1. Recording forms (Electronic Case Report Form: eCRF)

Types of eCRF and deadlines

The eCRFs used in this test and the time limit of their submission (input) will be as follows:

1) Before treatment start: Within 2 weeks after the registration
   1)-1 Background factors
   1)-2 Examinations
2) Therapeutic course: Within 6 weeks after discontinuation/completion of the protocol treatment
   2)-1 Treatments
   2)-2 Adverse events
   2)-3 Examinations

< Notes on eCRF >

- Responses to data input and inquiries shall be conducted via the EDC system.
- For details of the input method, refer to the input manual separately.
- If you are unsure how to input any of the data, record this in a note section for institutions. The Study Secretariat will correct the data later in some cases. Confirmation that data has been corrected, will be via e-mail from the Data Center at the inquiry timing. Please confirm receipt of the email.
- To avoid the risk of patient personal information leakage, medical record numbers shall not be used in any contacts with the Data Center. Instead, the patient registration number shall be used.

Storage of eCRF data

Data input to eCRF will be transferred from the Data Center to the J-SUPPORT Administrative Office after the date of the submission of the main analysis report. The storage period in the J-SUPPORT Administrative Office shall be semi-permanent given the possibility of additional uses such as secondary research.

Correction of eCRF

When deficiencies such as lack of data items necessary for eCRF or inappropriate category classification are found after the initiation of the study, the eCRF will be revised under agreement of the Data Center and Study Secretariat. This is permitted as long as the correction is judged not to exceed the range of collected data prescribed in section 8, “Evaluation items, clinical examinations, and evaluation schedule" and only if it does not increase the medical and economic burden of registered patients. The report of the correction to the director of the institution and submission of the revision will be done according to the rules of each institution.

10. Report of adverse events

In accordance with the provisions of this chapter based on the "JCTN Adverse Event Reporting Guidelines", the Institution’s principal investigator will report the occurrence of "serious adverse event" or "unexpected adverse event" to the Study Secretariat when they are observed.

The latest version of the report form should be used; it can be obtained on the JCTN website (http://jctn.jp/guideline_03.html).

Reports on adverse effects should be directed to the Minister of Health, Labour and Welfare based on the "Law for Ensuring the Quality, Efficacy, and Safety of Drugs and Medical Devices" (Address: Safety Information Division, Office of Safety I, Pharmaceuticals and Medical Devices Agency, FAX: 0120-395-390, E-mail: anzensei-hokoku@pmda.go.jp) [1].

Reports on serious adverse events should be provided to the director of each medical institution based on the "Ethical Guidelines for Medical and Health Research Involving Human Subjects" (Notification No. 3 of the Ministry of Education, Culture, Sports, Science and Technology and the Ministry of Health, Labour and Welfare, 2014) [2)]. Reports on unexpected serious adverse events from the director of a medical institution to the Minister of Health, Labour and Welfare, and notifications on adverse effects from medical institutions to companies should be appropriately made under the responsibility of the principal investigator at each medical institution and according to the rules of each institution.

1) http://www.info.pmda.go.jp/info/houkoku.html
2) http://www.mhlw.go.jp/stf/seisakunitsuite/bunya/hokabunya/kenkyujigyou/i-kenkyu/index.html

10.1. Adverse events that require reporting

Adverse events requiring emergency reporting

Adverse events falling under any of the following categories are subject to emergency reporting.

Most of these adverse events are likely a result of the original disease or anticancer drug associated with the patients in this study; therefore, only those that are judged to have a (definite, probable, or possible) causal relationship with the protocol treatment (APR, PALO, DEX, OLZ) of this study will be subject to emergency reporting.

1) Death

※ Even if a participant dies after the enrollment without receiving the protocol treatment, the participant shall be subject to emergency reporting because considerations of the eligibility at the time of the registration may be necessary.

2) Grade 4 Adverse events

3) Unexpected Grade 3/2/1 adverse events and those requiring hospitalization for more than 24 hours or extension of hospitalization * for the treatment of the adverse events

※ "Unexpected" refers to those not listed in section 7, “Expected adverse effects”.

※ "Hospitalization or extension of hospitalization period" refers only to those that require hospitalization/extension of hospitalization period longer than 24 hours for the treatment of adverse events; reporting is not required in the following cases:

- Hospitalization/extension of hospitalization during the follow-up period, although the adverse events already disappeared or lightened
- Hospitalization/extension of hospitalization period for the purpose of reducing patient burden, such as patients visiting from a remote location
- Hospitalization/extension of hospitalization period due to failure to improve nausea/vomiting caused by anti-cancer drugs
- Other hospitalizations/extensions of the hospitalization period that are not medical necessities.

4) Other adverse events judged as medically important conditions

Adverse events that do not fall under any of 1) to 3), but that are deemed sufficiently important to share among participating institutions. Adverse events associated with permanent or remarkable disorders (excluding MDS, secondary cancer, etc.), and other hereditary issues such as congenital anomalies.

However, if any of 1) to 4) encompass any of the following items a) to c), they are excluded from the emergency reporting:

a) Adverse events (including death) that occurred at least 31 days from the day of the last protocol treatment, those that can deny (either unlikely, not related) the causality with treatment

In cases where the protocol treatment was discontinued, even if posttreatment has already been started, emergency reports are still required if they are within 30 days of the last protocol treatment date. ("30 days" refers to 30 days (until Day 30) counting from the next day of the day of the last protocol treatment deeming as Day 0.)

b) Myelodysplastic syndrome (MDS), secondary cancer incidence

c) Adverse events not subject to emergency reporting specified below:

In this study, events for which remedies have already been established and are deemed unlikely to result in life-threatening situations as characteristics of the relevant disease or treatment, are excluded from the emergency reporting. Specifically, patients that do not die of the following adverse events are excluded from the emergency reporting and these adverse events are evaluated in monitoring reports.

SOC* (CTCAE ver. 4.0)	AE term
Blood and lymphocyte disorders	Anemia, hypocellular marrow
Gastrointestinal disorders	Constipation
Systemic disorders and local symptoms	Fever
Laboratory tests	Increased alkaline phosphatase level, decreased CD4 lymphocyte count, increased cholesterol level, increased CPK level, increased GGT level, increased lipase level, decreased lymphocyte count, decreased neutrophil counts, thrombocytopenia, increased serum amylase level, leukopenia
Metabolism and malnutrition	Obesity, anorexia, hyperuricemia, hypoalbuminemia, hypertriglyceridemia, hypoglycemia, hypokalemia, hypomagnesemia, hyponatremia
Renal and urinary tract disorders	Chronic kidney disease
Respiratory, thoracic and mediastinal disorders	Sleep apnea
Skin and subcutaneous tissue disorders	Hypohidrosis

* SOC: System Organ Class

10.2. Reporting obligation and reporting procedures of institutional research directors

Emergency report

If an adverse event that is subject to emergency reporting occurs, the attending physician will promptly notify the principal investigator in the institution. If the principal investigator is not available, an institutional coordinator or the attending physician shall act on behalf of the principal investigator. The principal investigator will report according to the following procedures. Be careful not to include personal identification information such as patient name and medical record number when sending the report.

1) Death or Grade 4 adverse event

Primary report:

The attending physician who knew of the occurrence of an adverse event will promptly report it to the principal investigator in the institution. The principal investigator who received the report will fill in specified items as far as possible in the "Adverse event report" within 72 hours of being informed of the occurrence of the adverse event, and will contact the Research Secretariat by e-mail, fax or telephone.

Secondary report:

The principal investigator in the institution will add detailed information regarding the adverse event to the "Adverse event report" within seven days of being informed of the occurrence of the adverse event, and will send it to the Research Secretariat by either e-mail, fax, postal or personal delivery. If necessary, copies of examination data, images, autopsy result reports, etc. shall be attached.

2) Adverse events of Grade 3 or lower grades of 10.1.3) or other adverse events judged to be medically important conditions

The attending physician who knew of the occurrence of an adverse event will promptly report it to the principal investigator in the institution. The principal investigator who received the report will enter the detailed information of the adverse event in the "Adverse event report" within 10 days of being informed of the occurrence of the adverse event and will report it. Examination data, images, and the autopsy result report will be attached if

necessary.

3) Additional reports

When new information is obtained after sending the above reports, the principal investigator will add the information in a prescribed form and will report this at any time. The summary of adverse events subject to emergency reporting and their report deadlines is shown in the table below.

Adverse events subject to emergency reporting and reporting deadlines

Causal relationship	Grade 1/2/3				Grade 4		Death		Other medically important conditions
	Expected		Not expected		Expected	Not expected	Expected	Not expected	
	No hospitalization	Hospitalization	No hospitalization	Hospitalization					
Present	Report not required	Report not required	Report not required	First time: Within 10 days Additional report: As needed	Primary report: Within 72 hours Secondary report: Within 7 days Additional report: As needed				First time: Within 10 days Additional report: As needed
Absent	Report not required	Report not required	Report not required	< Only during the treatment or within 30 days from the final day of protocol treatment >					
				First time: Within 10 days Additional report: As needed	Primary report: Within 72 hours Secondary report: Within 7 days Additional report: As needed				First time: Within 10 days Additional report: As needed

Report to the director of the medical institution

If an adverse event that is subject to an emergency report occurs, the principal investigator will report it to the director of the relevant medical institution in accordance with the provisions of the relevant medical institution as a "serious adverse event" described in the "Ethical Guidelines for Medical and Health Research Involving Human Subjects".

Report to other report destinations

The following reports mandated by the regulations applicable to this study will be appropriately made and are the responsibility of each facility according to the provisions of each medical institution.

Report on product safety information of pharmaceuticals, medical equipment and regenerative medicine

The information that is deemed necessary to report to the Minister of Health, Labour and Welfare based on Article 68-10 Paragraph 2 of the Law for Ensuring the Quality, Efficacy, and Safety of Drugs and Medical Devices shall be reported.

10.3. Responsibility of the Study Representative/Study Secretariat

Determination of necessity of discontinuation of the registration and urgent notification to participating institutions

Upon receiving a report from a principal investigator, the Study Secretariat will report it to and consult with the Study Representative, determine the urgency, importance, severity of influence, etc. of the report contents, and take measures such as temporary suspension of the registration (contact the Data Center and all participating institutions) and immediately contact participating institutions to ensure appropriate measures are taken. For contacting the Data Center and institutions, notification in written document will be done promptly done (either by email, fax, postal or personal delivery), although phone call is available depending on the urgency.

The Study Secretariat will report the occurrence of the adverse event to the administrative office of J-SUPPORT.

Subsequent contact for the exchange of information will be done between the J-SUPPORT administrative office and individual investigators in some cases.

Report to the Independent Data Monitoring Committee

When the Office of Independent Data monitoring Committee judges that the adverse event urgently reported from an institution falls under section 10.1, “Adverse events with reporting obligation ", it will consult with the Study Representative and report to the Office of the Independent Data Monitoring Committee within 15 days after being informed of the occurrence of the adverse event in writing. At the same time, the J-SUPPORT administrative office will request the Independent Data Monitoring Committee to review the validity of the viewpoint of the Study Representative and the response to the adverse event.

At that time, the results of consideration and countermeasures (such as the judgment of continuing/discontinuing the examination) to the adverse event taken by the Study Representative /Study Secretariat will be added to the "Adverse Event Report" sent from the institution. Furthermore, for deaths and expected Grade 4 adverse events (described in section 10.2.1), considerations on whether the occurrence frequency is within the expected range or not will be included, in addition to clinical course of the individual patient. If the incidence exceeds the expected range, it shall be described in the "Other serious medical events" section.

Notice to investigators in institutions

When reporting to the Independent Data Monitoring Committee, the Study Representative/Study Secretariat will provide the contents of the review and the recommendation of the Independent Data Monitoring Committee to principal investigators in all participating institutions by writing (e-mail is acceptable).

Even if the adverse event was not reported to the Independent Data Monitoring Committee, the Study Secretariat/the Study Representative will provide their judgment in writing (e-mail is acceptable) to the principal investigator of the institution.

Examination of adverse events in periodic monitoring

Upon periodic monitoring, the Study Representative / Study Secretariat will carefully examine the adverse event report on the monitoring report prepared by the Data Center, and will confirm that there are no omissions on the report from institutions. Conversely, it should also be confirmed that all reported adverse events are listed in the periodical monitoring report. The presence of omissions on the report will be clearly stated in the periodic monitoring report.

10.4. Response of principal investigators of participating institutions (including relevant institution)

Principal investigators of participating institutions in this study will respond according to the instructions of the Study Secretariat/the Study Representative. When the adverse event is subject to emergency reporting, the principal investigator will report it to the director of the medical institution as a "serious adverse event" described in the "Ethical Guidelines for Medical and Health Research Involving Human Subjects" in accordance with the rules of the relevant medical institution.

10.5. Response of the director of the institution to the occurrence of an adverse event

Upon the occurrence of an unexpected serious adverse event that is deemed to have a (either definite, probable, or possible) causal relationship with any protocol treatment, the director of the medical institution where the adverse event occurred will report it to the Minister of Health, Labour and Welfare according to the "Ethical Guidelines for Medical and Health Research Involving Human Subjects". The results of the examination by the Ethics Review Committee will be made publicly available.

10.6. Review by the Independent Data Monitoring Committee

The Independent Data Monitoring Committee will review and examine the contents of the report and will make a recommendation to the in writing about the future response, including whether or not to continue the registration and whether or not the protocol should be revised or amended.

11. Determination of the efficacy and definition of endpoints

11.1. Determination of the efficacy

1) Vomiting (including retching)

Subjects will record the presence/absence and the frequency of vomiting (including retching) in the symptom diary every 24 hours during the efficacy evaluation period, that is, for 120 hours from the initiation of the CDDP administration. Regarding the initial vomiting (including retching), the date and time of the onset should be recorded.

Vomiting is defined as the ejection of gastric contents such as gastric juice, food and the like out of the mouth via the esophagus. If there is an action to induce vomiting, even if gastric contents are not discharged (retching), this is also included as a vomiting episode.

2) Nausea

Subjects will evaluate and record the severity of nausea (a feeling of that vomiting is imminent) every 24 hours for 120 hours from the initiation of the CDDP administration using the 4-grade categorical scale shown below. Basically, subjects themselves judge their condition based on their own subjective feeling and record this self evaluation in the symptom diary.

* Nausea over the overall phase is evaluated with a maximum score using a 4-grade categorical scale.

Nausea: 4-grade categorical scale
0: No nausea 1: Mild 2: Moderate 3: Severe

3) Antiemetic treatment

If nausea / vomiting develops due to CDDP administration and the attending physician considers it necessary, or when the subject desires antiemetic therapy, appropriate measures will be taken. Drugs and others described in Table 6.2, “Unacceptable combination drugs during the protocol treatment" will be used, and procedures that are routinely conducted in each facility will be provided. However, additional administration of OLZ will not be allowed. Furthermore, medicines other than the drugs that are incorporated in the regimen beforehand in a manner aimed preventing nausea and vomiting will not be allowed. For treatment taken during the protocol treatment (see section 6.3, “Definition of Protocol Treatment Completion"), the date and time of implementation will be entered in the eCRF. The type and dose of antiemetic treatment drug shall not be prescribed.

4) Anorexia

Subjects will evaluate and record the severity of anorexia (severity of loss of appetite compared with usual appetite) every 24 hours until 120 hours from the initiation of the CDDP administration using the 4-grade categorical scale below. Basically, subjects themselves will judge their condition based on their own subjective feeling and record this self evaluation in the symptom diary.

* Anorexia over the overall phase is evaluated at the maximum value using a 4-point categorical scale.

Anorexia: 4-point categorical scale
0: Not at all 1: Very little 2: Somewhat 3: To a great extent

5) Drowsiness

Subjects will evaluate and record drowsiness every 24 hours until 120 hours from the initiation of the CDDP administration using a 4-grade categorical scale below. The severity of drowsiness during the daytime, disturbance of daytime behavior due to drowsiness, and hindrance to sleep at night will be evaluated. Basically, subjects themselves will judge their condition based on their own subjective feeling and record this self evaluation in the symptom diary.

* Drowsiness over the overall phase will be evaluated at the maximum value using a 4-grade categorical scale.

Drowsiness: 4-point categorical scale<br>1. Drowsiness during the day (feeling of drowsiness)<br>0: Not at all 1: Very little 2: Somewhat 3: To a great extent<br>2. Disturbance of daytime behavior due to drowsiness<br>0: Not at all 1: Very little 2: Somewhat 3: To a great extent<br>3. Hindrance to sleep at night<br>0: Not at all 1: Very little 2: Somewhat 3: To a great extent

6) Complete vomiting control (Complete Response: CR)
No vomiting and retching, no additional administration of antiemetic

7) Complete nausea and vomiting control (Complete Control: CC)
No vomiting and retching, no additional administration of antiemetic, no nausea or mild nausea
* No nausea ("0" in a 4-grade categorical scale) or mild nausea ("1" in a 4-grade categorical scale)

8) Total nausea and vomiting control (Total Control: TC)
No vomiting and retching, no additional administration of antiemetic, no nausea
* No nausea ("0" in a 4-grade categorical scale)

9) Time to Treatment Failure (TTF)
The time to the first vomiting (including retching) or additional administration of an antiemetic agent, whichever comes first.

11.2. Definition of endpoints

Endpoint for the efficacy	Vomiting (Including retching)	Antiemetic treatment by additional administration of antiemetic agent	Severity of nausea
CR rate (Complete Response Rate)	None	None	Does not matter
CC rate (Complete Control Rate)	None	None	None or mild
TC Rate (Total Control Rate)	None	None	None
TTF: Time to Treatment Failure	Starting from the start time of CDDP administration, the time until the earlier of the following events:<br>· Occurrence of the initial vomiting (including retching)<br>· Antiemetic treatment by additional administration of an antiemetic agent		

1) CR rate from 24 hours after the initiation of CDDP administration to 120 hours later (the delayed phase)
2) CR rate within 24 hours (the acute phase) and within 120 hours (the overall phase) after the initiation of CDDP administration

Complete nausea and vomiting control rate (Complete Control Rate)

Complete nausea and vomiting control rate within 120 hours after the start time of CDDP administration (the overall phase) and every 24 hours

Total nausea and vomiting control rate (Total Control Rate)

Total nausea and vomiting control rate within 120 hours after the start time of CDDP administration (the overall phase) and every 24 hours

Time to Treatment Failure

The time to the first vomiting (including retching) or the administration of additional administration of an antiemetic agent, whichever comes first. If there is no vomiting and no additional antiemetic treatment is observed, observation will be discontinued at 120 hours.

Incidence of nausea

The incidence of nausea as defined in section 11.1.2. In a 4-grade evaluation from "0: No nausea" to "3: Severe", "1: Mild" or worse is defined as "having nausea".

Incidence of anorexia

The incidence of anorexia as defined in section 11.1.4. In a 4-grade evaluation from "0: Not at all" to" 3: To a great extent", "1: Very little" or worse is defined as "having anorexia".

Evaluation of drowsiness

Regarding the symptoms of drowsiness as defined in section 11.1.5, the incidence of 1. Drowsiness during the daytime, 2. Disturbance of daytime behavior due to drowsiness, and 3. Hindrance to sleep at night are confirmed, and "1: Very little" or worse is defined as "having drowsiness".

Incidence of adverse events

For the following adverse events (toxicity), the frequency of the worst grade during protocol treatment based on CTCAE v4.0-JCOG is obtained for each group, taking the all treatment cases as a denominator.

Laboratory test values: Increased blood bilirubin level, increased aspartate aminotransferase (AST or GOT) level, increased alanine aminotransferase (ALT or GPT) level

Gastrointestinal disorders: Constipation, hiccups, dry mouth

Nervous system disorders: Somnolence, insomnia, dizziness

Adverse events (toxicity) other than the above, are described on a treatment progress record form only when non-hematologic toxicity of grade 3 or worse is observed; therefore, as a general rule, the incidences are not counted unless specific adverse events are frequently observed.

* "Non-hematological toxicity" refers to adverse events other than the following in CTCAE v4.0-JCOG:

"Anemia", "bone marrow cell depletion", "decreased lymphocyte count", "decreased neutrophils count", "decreased lymphocyte count", "decreased platelet count", "decreased CD4 lymphocyte count"

Incidence of serious adverse events (adverse effects)

1) Early death rate

The ratio of the number of all deaths during the protocol treatment period or within 30 days after the day of the last protocol treatment as a numerator with all treatment cases as denominators. Regarding the cause of death, a causal relationship to the protocol treatment is not required.

2) Incidence of treatment-related death (TRD)

The ratio of the number of deaths thought to be caused by protocol treatment (definite, probable, or possible) among all of the deaths as a numerator, with all treatment cases as denominators

12. Statistical matters

Statistical analysis staff will separately prepare a statistical analysis plan that will be in place before data are recorded.

12.1. Analysis sets

The definition of the analysis sets in this study is as follows. When it is unclear how data from a patient should be handled based on the criteria described below, the statistical analysis director, the Study Representative, and the Study Secretariat will hold consultations before data fixing; all these people will remain blinded.

Definition of analysis sets in this study

Analysis set	Definition
Full Analysis Set	All registered cases (except mistaken registrations, such as double registration)
Efficacy analysis set	A population excluding patients satisfying the following criteria from the "all registered cases"<br>- Patients found to be clearly ineligible at the registration<br>- Patients who have not received HEC treatment prescribed in this protocol (CDDP < 50 mg/m^2)<br>- The patient who received no protocol treatment
Safety analysis set	All patients who received a part of or all of the protocol treatment

12.2. Analyses of the primary endpoint

The CR ratio, the point estimate of intergroup difference, and the 95% confidence interval in the delayed phase in each group will be calculated, and Mantel-Haenszel test adjusted with age (55 years old and older/under 55 years old), gender, and CDDP dose (≥ 70 mg/m^2 / < 70 mg/m^2), which are allocation adjustment factors, will be performed. The significance level of the one-sided test shall be 2.5%. However, when the number of patients in each layer is small, the final decision as to how the adjustment factors should be handled will be recorded in the analysis plan document.

12.3. Analyses of the secondary endpoints for efficacy

In the analysis of the secondary endpoints for efficacy, adjustment with the allocation adjustment factors will not be performed in principle. The point estimate of intergroup difference, and the 95% confidence interval will be calculated and a Mantel-Haenszel test will be performed for the CR rate in the acute phase and the overall phase, the complete nausea and vomiting control rate (CC rate) in the overall phase and every 24 hours during the period, and the total nausea and vomiting control rate (TC rate) in the overall phase and every 24 hours during the period. For the Time to Treatment Failure, the median estimate and 95% confidence interval for each group will be calculated using the Kaplan-Meier method, and group comparison will be performed using the log rank test. Regarding the severities of nausea, anorexia and drowsiness, and the impact on daily life, the distribution of Grade in each group shall be described and comparison between groups shall be performed using a Mantel test. The significance level of the two-sided test shall be 5%.

12.4. Safety analysis

For adverse events, the distribution of Grade in each group will described, and comparisons between groups using a Mantel test will be conducted. The significance level of the two-sided test shall be 5%.

12.5. Rationale for setting planned number of enrollments

Based on section 2.2, “Standard treatment for subjects", when a superiority test design that detects whether the CR ratio of group B exceeds 10% or not is used with assuming that the CR ratio in the delayed phase of Group A is 65% and considering the toxicity due to the addition of OLZ, and when the one-side significance level is 2.5% and the detection power is 80%, the number of required analysis targets will be 329 cased per group and 658 cases in total. In anticipation of a 5% of ineligible cases and cases without receiving treatment, the number of scheduled registrations will be set to 690 cases.

12.6. Interim analysis

Interim analysis is not carried out.

13. Ethical matters

13.1. Protection of patients

All investigators will conduct this study in accordance with the "Helsinki Declaration" (translated by the Japan Medical Association) [1)] and the "Ethical Guidelines for Medical and Health Research Involving Human Subjects" (Notification No.

3 of the Ministry of Education, Culture, Sports, Science and Technology and the Ministry of Health, Labour and Welfare, 2014) [2)].

"Medical institutions" in this protocol corresponds to "research institutions and collaborative research institutions" in the above guidelines.

1) http://dl.med.or.jp/dl-med/wma/helsinki2013j.pdf
2) http://www.mhlw.go.jp/stf/seisakunitsuite/bunya/hokabunya/kenkyujigyou/i-kenkyu/index.html

13.2. Informed consent

Explanation to the patients

Prior to patient registration, the attending physician hands them the explanatory document, which has been approved by the medical institution, and the following contents will be explained in detail verbally. "Approval of medical institution" in this protocol means that an approval document issued by the director of the medical institution is available to the applied researcher based on the result of the review by the Institutional Review Board (IRB) * consulted by the director of the medical institution.

* It does not matter whether an IRB has been established in the physician's own institution or elsewhere.

Explanation contents

1) Explanation on disease name and chemotherapy
2) Explanation that this study is a clinical trial and it is a study carried out by the Abe group
3) Study design and the rationale for this study
4) Contents of the protocol treatment
5) Expected efficacy of the protocol treatment
6) Expected adverse events
   With regard to complications, sequelae and how to deal with them, explanation of the extent and frequency of expected adverse events including complications, sequelae, treatment-related deaths, and how to deal with them when they occur.
7) The fact that follow up care after the completion of the protocol treatment will be properly performed
8) Cost burden and compensation
   Explanation that these elements are similar to those encountered in general practice. For example, the cost of treatment is covered by the health insurance system and partially by research expenses, and compensation in case of damage to health is based upon the usual response in general practice.
9) Alternative treatment options
   Explanation of treatments that can be taken if the patient does not participate in this study
10) Anticipated benefit and possible disadvantages
    Explanation of the benefits that may be enjoyed by participating in the study and the disadvantages that may be experienced
11) Direct access to medical history
    Explanation of the acceptance of audit such as "Healthcare professionals of other medical institutions may directly review medical history and others for quality control with the permission of the director of the medical institution."
12) Consent refusal and consent withdrawal
    Explanation that the patients are free to refuse consent before participating in the study, and to withdraw after consent has been given without unfair medical disadvantage
13) Explanation that the utmost efforts will be paid to protect human rights, and to maintain the privacy with regard to the patient's name and their personal information.
14) Conflicts of interest
15) Secondary usage of data
    Only upon approval by J-SUPPORT, the data may be subject to secondary use (for meta-analysis, etc.) in a

form not linked with personal identification information.

16) Method of Information disclosure regarding the study

17) Freedom of questioning
Explanation that the patients can freely ask questions about the study and treatment. Information in a printed document will provide not only the contact details of the physicians in charge, but also the contact address of the principal investigator of the medical institution, the Study Representative (or the Study Secretariat).

Consent

A patient can only be asked to give consent to participate in the study after he/she has been given an explanation of the study, has been given sufficient time to think, and has confirmed that they have throroughly understood the study scope and content. When the patient him/herself gives consent to participate in the study, his/her signature shall be obtained using the consent form of the study approved by the medical institution. The attending physician confirms the recording of the name of physician who explained the study, the date of explanation, the name of the patient who received the explanation and consented, and the date of consent in the consent form.

Two duplicates of the consent form will be prepared; one will be handed to the patient himself, and another copy will be kept by the institutional principal investigator or an institutional coordinator. The original copy will be kept in a storage location prescribed in the medical record or by the medical institution.

Response to inquiries, consultation etc. after the consent

Any consultation or enquiries related to the study that involve the patient or his/her family and that occur after registration, should (in principle) be responded to by investigators of the medical institution responsible for the patient (principal investigator, institutional coordinator, attending physician). If the method of correspondence is unknown, a response will be provided after consultation between the Study Secretariat, the Study Representative and the Data Center.

13.3. Protection of personal information and patient identification

Patient identification is possible only in the patient correspondence table in which both the registration number dedicated to a study at the time of case registration and the patient chart ID are listed. Information leading to patient identification is managed by the personal information manager and never goes to third parties. Exchange of patient data shall be conducted in a manner that places the greatest importance on protecting privacy, regardless of whether exchanges are via paper or electronic media.

Storage of data and others

The storage limitation of the data on this study at the participating institution shall be 5 years from the date of submission of the main analysis report, or 3 years from the publication date of all the papers related to this study, whichever is later, and it is recommended to keep data as long as possible even after this storage limitation. When discarding the samples and information gathered during this trial after the storage period, all data should be anonymized.

Secondary usage of data

The data obtained in this study may be used in secondary analyses (for meta-analysis, etc.) in a form not linked with personal identification information. Compliance with the terms of such usage is the responsibility of the Study Representative.

13.4. Compliance with the protocol

Investigators participating in this study shall comply with this protocol unless in doing so they compromise the safety and/or human rights of patients.

13.5. Approval of the Ethics Review Committee of medical institution

Approval at the start of study participation

Upon participation in this study, approval shall be obtained from the director of each medical institution concerning the

execution of the study using this protocol and the provision of explanatory documents for the patient. Modification of the explanatory documents for patients can be made at each medical institution within limits that ensure there is no deviation from the requirements of clinical trials. These modifications require the approval of the relevant medical institution. However, there can be no modifications of the actual treatment protocol at any of the participating medical institutions..A protocol common to all facilities shall be used. When content change is necessary and if there is a request from a medical institution to modify the text of the protocol, the institutional coordinator shall consult with the Study Secretariat. This is because revision or amendment of a protocol must then extend to all institutions

Annual renewal of the approval by each medical institution

Annual renewal of the study approval for this protocol and of the patient explanation document shall be carried out by each medical institution in accordance with their own provisions.

13.6. Changes to the contents of the protocol

Classification of changes to the contents of the protocol

Before changing the protocol content, a "protocol revision application" must be submitted to the Independent Data Monitoring Committee; their approval must be obtained prior to the substitution with the changed contents. However, extension of the registration period within 6 months is not subject to these protocol revision procedures.

After the approval of the protocol review committee, J-SUPPORT will treat the changes in the protocol contents by dividing them into three types: amendment, revision, and memorandum. As a general rule, the change application will be submitted to the J-SUPPORT area director, who together with the leader will decide the correct classification for the change. When the application is deemed as a revision, the memorandom or revision can be approved by J-SUPPORT area director's judgment. If the area director deems it necessary to revise, it will be necessary to submit a report to the Independent Data Monitoring Committee to obtain approval.

1) Amendment

Partial modification of protocols that may increase risk to the health of patients participating in the study, or that have a substantial effect on the primary endpoint of the study. Approval of the Independent Data Monitoring Committee and each medical institution will be required.

After approval of the changed contents by the J -SUPPORT area director, a report is submitted to the Independent Data Monitoring Committee.

When the Independent Data Monitoring Committee approves the change, the approval date and effective date of the Independent Data Monitoring Committee shall be indicated on the cover page of the protocol.

If patient registration was ongoing at the time when the Independent Data Monitoring Committee determined that the protocol modification type was equivalent to an "Amendment", further registration will be suspended and approval of the revision contents will be obtained from each medical institution. When approval is obtained, the facility coordinator of each medical institution will send a duplicate of the approval document of each medical institution to the administrative office of J-SUPPORT. The registration will be resumed sequentially from the institution where the approval document is confirmed.

2) Revision

Modification of protocols that are unlikely to increase risk to the health of patients participating in the study and that do not have a substantial effect on the primary endpoint of the study. Such modifications require approval of the J - SUPPORT area director and each medical institution. It is up to the judgment of each medical institution to decide whether the review type at each medical institution falls into the ‘regular review’ or ‘prompt review’ category. As a general rule, “Revisions” do not trigger temporary suspension of patient registration.

The date of approval and effective date on J-SUPPORT shall be indicated on the cover page of the protocol.

Even before the approval of the medical institution after the effective date, the study will be carried out according to the revised content, which was approved, in principle. If the details of the revision cannot be effective until the approval of the medical institution due to circumstances at that particular institution, the Study Secretariat and the J-SUPPORT Administrative Office should be consulted. It is not necessary to send a duplicate of the approval documents of each medical institution to the Data Center. However, when approval is obtained at each medical institution, the original approval document shall be kept by the facility coordinator, because it will be checked at the time of audit.

3) Memorandum

This is a supplementary explanation of the protocol distributed from the Study Representative/the Study Secretariat to all concerned parties. Its purpose is to, reduce variations in the interpretation of the context of the protocol, rather than to provide advice on how to change the contents of the protocol. It is especially written in order to explain how to flag potentially dangerous issues that may arise during the study. The format does not matter. In addition, although it corresponds to the change of the protocol contents, if it is deemed that information should be promptly shared among investigators engaged in the study in order to reduce the risk to registered patients, memorandums based on the premise of revision application shall be issued.

Approval by the J-SUPPORT area director is required before distribution. Furthermore, the memorandum should be reported to the Independent Data Monitoring Committee both before and after it has been distributed. Indication of a single memorandum on the cover page of the protocol is unnecessary. However, if memorandums are issued several times, they should all be moved to the revised work section.

Explanation to registered patients at protocol revision/amendment

The attending physician shall make an appropriate explanation (regarding protocol treatment and follow-up, etc.) to the registered patient in the case of amendment or revision. This is because there is a statement in the explanatory document that changes in the contents of the study should be promptly relayed to the patient. Alternatively, in the case of revision, the attending physician will make an appropriate explanation (regarding protocol treatment and follow-up etc.) to the registered patients.

Correction of eCRF

In cases where defects such as missing data items necessary for eCRF and inappropriate category classification are found after the start of the study, the eCRF should be modified on agreement between the Data Center and the Study Secretariat to the extent that it is judged not to exceed the range of collected data prescribed in section 8, “Evaluation items, clinical examinations, and evaluation schedule"; it should also not increase the medical and economic burden of the registered patients. Correction of an eCRF that does not require revision of the body of the protocol will not be classed as a revision of protocol. Reports on the correction of an eCRF, or applications for revision, will be sent to the director of the medical institution according to that particular institute’s rules.

13.7 Management of conflict of interest (COI)

This section regards COI management by those involved in J-SUPPORT research

The COI of investigators and those supporting the study shall be managed according to the provisions of the medical institution to which they belong.

The conflicts of interest to be disclosed related to this study are as follows:

Study Representative
No conflicts of interest need be disclosed.

Study Secretariat
No conflicts of interest need be disclosed.

13.8 Compensation

Regarding damage to health caused by participating in this clinical trial: we provide adequate medical treatment under health insurance according to the disease conditions in the same manner as usual medical care. When treatment is required, the burden of the medical expenses will be borne by the patient. In addition, we do not provide economic compensation such as money and various allowances.

13.9 Intellectual property

Results, data, and intellectual property rights obtained by this clinical trial are attributed to three parties: the Study Representative, the Study Secretariat and the National Cancer Center. The specific handling and allocation will be

discussed and decided by the three parties. Whether to assign the intellectual property to the Study Representative or the Study Secretariat, or to the individuals or affiliated medical institutions will be decided based on that particular institute's rules

13.10 Information disclosure on this study

The outline, current status, and main results of this study will be published on the websites of J-SUPPORT (www.j-support.org) and UMIN-CTR (www.umin.ac.jp/ctr/). The UMIN study ID of this study is UMIN000024676.

14 Monitoring and auditing

14.1 Periodic monitoring

This study implements regular monitoring twice a year, in principle, according to the "JCTN Central Monitoring Guidelines" (http://jctn.jp/guideline.html). Such monitoring is designed to confirm whether the study is conducted safely and in accordance with the protocol, and whether the data are accurately collected.

This monitoring is centralized and conducted mainly by the Data Center, Research Executive Office and principal researcher based on the eCRF entry data that is fed back to the Data Center. Facility visiting monitoring to check the data with the source materials and others will not be implemented.

The Data Center will submit a periodic monitor report to the Study Secretariat, Study Representative, and Independent Data Monitoring Committee. The report is examined according to the J-SUPPORT regulations concerning monitoring.

The purpose of the periodic monitoring is to improve the scientific ethics of the study by feeding back problems, but is not intended to detect problems of the study and institutions. Therefore, the Study Representative will review the regular monitoring report distributed before or on the day at each group meeting, and the Study Secretariat and institutional principal investigators will share information about the problems pointed out in the report, and will then work with investigators in participating institutions to resolve such problems.

Monitoring items

① Registration status: Number of registrations - cumulation/by period, by institution
② Eligibility: Ineligible cases / potentially ineligible patients: Institution
③ Baseline background factors
④ Discrimination during the protocol treatment / termination of the protocol treatment, reason of discontinuation / termination: Institution
⑤ Protocol deviation: Institution
⑥ Serious adverse events: Institution
⑦ Adverse effects / adverse events
⑧ Overall survival, progression-free survival (or recurrence-free survival): All registered patients
⑨ Others, issues related to the progress and safety of the study

Eligibility (eligibility / ineligibility)

For all registered patients, eligibility is classified as one of the following, according to the definitions below. Upon monitoring, the Data Center lists the patients that may be classed as unqualified under the "Evaluation of eligibility" section in the monitoring report, and eventually, either 1), 2), 9), or 99) is decided before the implementation of the main analysis and after review by the Study Secretariat. Only category 1 patients are eligible, where as category 2, 9, and 99 patients will be classed as ineligible.

1) Eligible
According to the method and criteria prescribed in the protocol, the information generated before the registration meets all patient selection criteria.
2) Ineligible after registration
One of the patient selection criteria is not satisfied because information was generated after the registration. Alternatively, information may have been passed on before registration, but one of the patient selection criteria used was not specified in the current study protocol.
9) Ineligible at the time of registration
Information generated prior to the registration according to the method (performed in all cases) prescribed by

the protocol and the criteria do not satisfy any of the patient selection criteria. This category also includes cases where it was found after the registration that the information provided before the registration was incorrect.

99) Violating registration
In cases where a registration has been deliberately falsified (falsely) in the knowledge that it does not meet the patient selection criteria. Violating the terms of registration is treated as a serious problem and is equivalent to a false report.

Protocol violation / deviation

When treatments such as drug administration, radiation treatment, qnd surgical excision, as well as clinical examination, and evaluation of toxicity and efficacy are not carried out according to the protocol, they are deemed protocol deviations. Deviations beyond a range that is decided on a study-by-study basis by the Data Center and the Study Representative / Study Secretariat (in advance or after the start of the study) are listed in the monitoring report as "Possible deviations", and will be classified as one of the following after review by the Study Secretariat and the study group.

1) Violation

A deviation from the provision of the protocol that is clinically inappropriate, caused by the physician / facility in charge, and corresponds to any of the following items shall be regarded as a violation.

① Substantially affect the evaluation of the study endpoint
② Intentional or systematical deviation
③ The severity of danger or departure is remarkable

In principle, the contents of individual "Violation" shall be described when publishing the paper.

2) Deviation

Deviations that do not fall under the violation of 1) or the accepted range of 3)

If there are many specific deviations, and these should be described when publishing the paper.

When examining monitoring reports, deviations are classified as one of the following.

1) Deviation・・・・・・・・・Deviations that are undesirable and should be reduced
2) Deviation (Inevitable)・・・Deviations that do not warrant active reduction (Example: postponement due to year-end and new year holidays, equipment ,etc.)
3) Deviation (Clinically reasonable) ・・・Deviations that are judged to have had a positive effect in the opinion of the attending physician / institution (i.e., this new line of action should be pursued in the future, should the same situation occur again)

※ Deviations do not mean that there is always a problem with the doctor in charge of the institution. Even in research, patient safety is the first priority in clinical trials; therefore, if it is judged dangerous to comply with the provisions of the protocol according to the condition of an individual patient, a deviation may be the preferred option, depending on the medical judgment of the attending physician. If a deviation is judged clinically reasonable in order to maintain a patient's safety, it is recorded as ③ "Deviation (Clinically reasonable)" as above. When there are few clinically reasonable deviations, there is no particular problem to be addressed. However, if there are multiple occurrences, it is highly likely that the provision of the protocol is inappropriate, land a revision of the protocol should be considered. On the other hand, deviations made with an intention other than safety (such as an increase in anticancer drugs in expectation of enhancing effectiveness, a reduction in the treatment period other than the protocol prescription) are not a "clinically reasonable deviation"

3）Acceptable deviation

Deviation from the protocol within the allowable range established for each study by the Study Representative / Study Secretariat and the Data Center; these may occur before or after the start of the study.

Deviations within a preset allowable range are not included in the monitoring report.

Withdrawal of consent

Withdrawal of consent means revocation of consent to participate in the study, and is distinguished from denial of the continuation of the protocol treatment (① below). When the withdrawal of consent is expressed, clarify whether it falls under ② or ③ below, and promptly contact the Data Center.

In the case of ② withdrawal of consent, the Data Center will stop requesting the subsequent follow-up according to protocol. In the case of ③, data on the patient is deleted from the database when it is confirmed that withdrawal of all consent is confirmed.

① Patient rejection: Continuation of subsequent protocol treatment is refused (follow-up is continued).

② Withdrawal of consent: Withdrawing the consent to participate in the study, and disabling all of the subsequent treatments and follow-ups according to the protocol. Research use of the data before the consent withdrawal is allowed.

③ Withdraw of all consent: Withdrawing the consent to participate in the study, and disabling research use of all data from the time of participation of the study, including information at the time of the registration

14.2 Facility visit audit

J-SUPPORT does not conduct facility visit audits. Each institution shall accept periodic internal audits.

15. Additional notes

15.1 Off-label use of drugs

In Japan, OLZ has not been approved for treatment of gastrointestinal symptoms (nausea and vomiting) that accompany administration of antineoplastic agents. With this in mind, a request from the Japanese Society for Palliative Medicine, Japanese Society for Pharmaceutical Palliative Care and Sciences, Japanese Association for Study of Pain, Japan Society of Pain Clinicians, and Japanese Society of Gastroenterology has been submitted to the "Evaluation Committee on Unapproved or Off-labeled Drugs with High Medical Needs" of the Ministry of Health, Labour and Welfare. In the planning stage of a previous Phase II trial on OLZ at 5 and 10 mg, companies could not donate OLZ, mainly because the OLZ patent is due to expire. Therefore, in the Phase II study, OLZ was purchased from research expenses and distributed to participating institutions. This was based on the answer to written question No. 380 on June 22, 2007, which was related to the issue of treatments partially covered by insurance, and the problem of giving and receiving unapproved drugs. The question was submitted by House of Representatives member Kazuko Ko-ori. Accordingly in this study, the test drug was purchased from research expenses (no economic burden on patients). The drugs listed in the drug price list were widely used in routine clinical settings that are described in the overseas guidelines; such usage was thus considered as falling within the doctor's discretion. The current study is carried out with the same considerations as this previous example.

16. Research organization / funding source

16.1 Main funding source for this study

Japan Agency for Medical Research and Development, "Practical Research for Innovative Cancer Control"

Research and development representative: Masakazu Abe (Shizuoka Cancer Center), "Research and development of a new antiemetic therapy for preventing chemotherapy-induced nausea and vomiting"

16.2 J-SUPPORT

J-SUPPORT (Japan Supportive, Palliative and Psychosocial Oncology Group) is a multicenter clinical research group that supports approved research according to the basic bylaw of J-SUPPORT and is one of several research groups that are funded by public and private research funds.

J-SUPPORT consists of a research group, which becomes a study-implementing entity; various committees, which are the audit and management organization; and an administrative office, which is the central support organization. J-SUPPORT has no corporate status and is a private organization.

The administration office is set up at the Supportive Care Development Center, the National Cancer Center Hospital.

16.3 Representative of J-SUPPORT

Yosuke Uchitomi
Innovation Center for Supportive, Palliative and Psychosocial Care, National Cancer Center Hospital
Adress: Tsukiji 5-1-1, Chuo-ku, Tokyo, 104-0045, Japan

Phone: +81-3-3542-2511 Ext. 2330
E-mail: yuchitom@ncc.go.jp

16.4 J-SUPPORT Administrative Office

Yutaka Matsuoka
Innovation Center for Supportive, Palliative and Psychosocial Care, National Cancer Center Hospital
Adress: Tsukiji 5-1-1, Chuo-ku, Tokyo, 104-0045, Japan
Phone: +81-3-3542-2511 Ext. 2330
Fax: +81-3-3543-5603
E-mail: ymatsuo@ncc.go.jp

16.5 Study Representative

Masakazu Abe
Department of Gynecologic Oncology, Shizuoka Cancer Center
Adress: 1007 Shimonagakubo, Nagaizumi-cho, Sunto-gun, Shizuoka Prefecture 411-8777, Japan
Phone: +81-55-989-5222 Ext. 6202
Fax: +81-55-989-5551
E-mail: ma.abe@scchr.jp

16.6 Study Secretariat

Hironobu Hashimoto
Department of Pharmacy, National Cancer Center Hospital
Adress: Tsukiji 5-1-1, Chuo-ku, Tokyo, 104-0045, Japan
Phone: +81-3-3542-2511 Ext. 3531
Fax: +81-3-3248-0730
E-mail: hhashimo@ncc.go.jp

16.7 Statistical analysis personnel

Takuhiro Yamaguchi
Biostatistics, Tohoku University Graduate School of Medicine,
Clinical Research Data Center, Tohoku University Hospital, Professor and Director
Adress: 1-1, Seiryo-machi, Aoba-ku, Sendai, Miyagi 980-8574, JAPAN
Phone: +81-22-717-7659
Fax: +81-22-717-7580
E-mail: yamaguchi@med.tohoku.ac.jp

16.8. Participating Institutions

Institution	Principal investigator	Institutional　coordinator
Hokkaido Cancer Center	Toru Harabayashi	Shinya Tamaki
Sapporo Medical University Hospital	Koichi Takada	Katsuyuki Nakamura
Hakodate Municipal Hospital	Jun Kimura	Yukio　Sakata
Hakodate Central General Hospital	Yusuke Sasaki	Matsuoka Keigo
Niigata Cancer Center Hospital	Hiroshi Tanaka	Satoru Miura
Gunma Prefectural Cancer Center	Koichi Minato	Yukiyoshi Fujita
Saitama Cancer Center	Hideaki Mizutani	Tomoaki Nakayama
National Cancer Center Hospital	Yuichiro Ohe	Takako Yanai
National Cancer Center Hospital East	Takashi Kojima	Hisanaga Nomura
Cancer Institute Hospital	Toshihiro Hama	Kenichi Suzuki
Tokyo Medical University Hospital	Tetsuya Okano	Ayako Torii
Showa University Hospital	Yutaro Kubota	Hisanori Shimizu
Shizuoka Cancer Center	Masakazu Abe	Hiroshi Ishikawa
Hamamatsu University Hospital	Naoki Inui	Emiko Otobe
Kouseiren Takaoka Hospital	Kazuhiko Shibata	Misae Takase
Gifu University Hospital	Yasushi Ohno	Hirotoshi Iihara
Osaka City General Hospital	Koji Takeda	Masahiko Nakao
Osaka University Hospital	Makoto Yamazaki	Yasuaki Sanada
Wakayama Medical University Hospital	Hiroki Ueda	Tomoyasu Nishimura
Kansai Rosai Hospital	Kimihiko Ito	Fukuko Shimokawa
Hyogo Prefectural Amagasaki General Medical Center	Katsuya Hirano	Natsuko Nishikubo
Kobe Minimally Invasive Cancer Center	Hideaki Okada	Atsushi Wada
Tottori University Hospital	Tasuku Harada	Miki Shimada
Hiroshima City Hiroshima Citizens Hospital	Yasuo Iwamoto	Yasuhiko Sakata
Kochi Health Science Center	Yuji Negi	Norifumi Miyamoto
Shikoku Cancer Center	Naoyuki Nogami	Yuuki Kogure
Fukuoka University Hospital	Masaki Fujita	Sumiyoshi Hatano
Kurume University Hospital	Shin Nishio	Hirokazu Matsumoto
Oita University Hospital	Tomoko Hirakawa	Ryosuke Tatuta
Kyusyu University Hospital	Keisuke Kodama	Akiko Kanaya

16.9 Independent Data Monitoring Committee

In this clinical trial, the Independent Data Monitoring Committee is established to obtain objective opinions from third parties when any trouble arises. Continuation or discontinuation of this study will be decided by this committee. New patient registration is suspended whenever the committee meets to discuss the decision to continue or discontinue.

The committee consists of one executive committee of J-SUPPORT; Kazuo Tamura, Fukuoka University School of Medicine; Toshiaki Saeki, Saitama Medical University International Medical Center; Eriko Satomi, National Cancer Center Hospital.

16.10 Auditing committee

Audit within an institution shall follow the rules established by each participating institution. For the entire study, J-SUPPORT will establish an auditing committee during or after the study to investigate whether any problems were encountered in the course of the study.

16.11. Data Center

Data Management Office, Clinical Research Support Division, National Cancer Center Hospital
Director of Data Center: Haruhiko Fukuda
Head of Data Management Office: Harumi Kaba
Adress: Tsukiji 5-1-1, Chuo-ku, Tokyo, 104-0045, Japan
Phone: +81-3-3547-5201
E-mail: NCCH_CDM@mi.res.ncc.go.jp

16.12. Protocol preparation

Department of Pharmacy, National Cancer Center Hospital — Hironobu Hashimoto
Takako Yanai
Department of Gastrointestinal Medical Oncology, , National Cancer Center Hospital — Satoru Iwasa
Department of Gynecologic Oncology, Shizuoka Cancer Center — Masakazu Abe
Department of Biostatistics, Xxxxxx University Graduate School of Medicine — Takuhiro Yamaguchi

Protocol preparation support
Department of Pharmacy, National Cancer Center Hospital — Hiroyuki Terakado
Department of Thoracic Oncology, National Cancer Center Hospital — Yuichiro Ohe
Noboru Yamamoto
Department of Gynecologic Radiation Oncology, National Cancer Center Hospital East — Sadamoto Zenda
Clinical Research Support Division, National Cancer Center Hospital, Data Management Office — Harumi Kaba
Responsible for data management — Haruhiko Fukuda

17. Publication of research results

The research results are published according to the agreement below. The person nominated by an institutional principal investigator is not necessarily the person belonging to the principal investigator's own institution.

17.1 Main paper

Publications for submission to a journal will have more than 10 authors and be written in the English language.

Selection of authors (authorship)

The authorship will be given to facilities with more than 30 registrations, and with precedence given to the facility with the largest number of registrations. The corresponding author and last author shall be persons appointed through discussions between the principal researcher, the Research Executive Office, and the J-SUPPORT representative regardless of the rank order.

(Example: When the registration percentage of the top 4 institutions exceeds 5% and the number of registrations up to 7th is 30 cases or more.)

First author: Person appointed by the principal investigator of the institution with 1st number of registrations.
Second author: Person appointed by the principal investigator of the institution with 2nd number of registrations.
Third author: Person appointed by the principal investigator of the institution with 3rd number of registrations.
4th author: Person appointed by the principal investigator of the institution with 4th number of registrations.
5th author: Person appointed by the statistical analysis personnel or J-SUPPORT representative.
6th author: Person appointed by the principal investigator of the institution with 5th number of registrations.
7th author: Person appointed by the principal investigator of the institution with 6th number of registrations.
8th author: Person appointed by the principal investigator of the institution with 7th number of registrations.
9th author: Person appointed by the principal investigator of the institution with 1st number of registrations.
10th author: Person appointed by the principal investigator of the institution with 2nd number of registrations.
11th author: Person appointed by the principal investigator of the institution with 3rd number of registrations.
12th author: Person appointed by the principal investigator of the institution with 4th number of registrations.
13th author: Person appointed by the statistical analysis personnel or J-SUPPORT representative.
14th author: Person appointed by the principal investigator of the institution with 5th number of registrations.
15th author: Person appointed by the principal investigator of the institution with 6th number of registrations.
16th author: Person appointed by the principle investigator of the institution with 7th number of registrations.

Last author: Shall be a person appointed through discussions between the Study Representative, the Study Secretariat, and the J - SUPPORT representative.

Corresponding author: Shall be a person appointed through discussions between the Study Representative, the Study Secretariat, and the J - SUPPORT representative.

If the registration number of the corresponding author's institution is outside the range of the authorship acquisition right, he/she will be the 3rd author, and the order of authors appointed by the statistical analysis personnel or J-SUPPORT representative and the institution with 3rd number of registrations shall be decreased by one. For the 17th author and downward, the Study Representative and the Study Secretariat will decide who will be appointed, whether they are included, and when they are included. Institutions that cannot acquire the authorship in the order of registration regardless of the registered number of 30 or more, any institution with a registered number of less than 30, and those appointed by the J-SUPPORT representative shall be included in the Acknowledgment.

If there is a problem with the above arrangement, discussions between the Study Representative, the Study Secretariat, and the J - SUPPORT representative will be undertaken to identify which journals should be targeted for submission.

An acknowledgment to the data manager in charge of the Data Center shall be described in the Acknowledgment.

Deadline of paper submission

The main paper shall be submitted within one year after the first conference presentation. If the paper cannot be submitted after the deadline, the representative of J-SUPPORT will initiate transfer of the first author's right to the second author, and downward thereafter.

17.2. Supplementary paper

The right to author supplementary papers will begin with the institution with the largest number of registrations. The right of statistical analysis personnel will be deemed to be equal to 5% of the total registered number or higher than the institution with 5th number of registrations.

However, any first authors of main papers are not permitted to be first authors in supplementary papers.

17.3. First presenter of academic presentation etc.

The first persons to present overseas and domestic academic presentations will be decided by the Study Representative and the Study Secretariat among the core members involved in the protocol preparation.

The first presenter of presentation at the first overseas conference

Those who acquired the right of the last author in the main paper shall nominate him/her.

The first presenter of presentation at the first domestic conference

Those who acquired the rights of the corresponding author in the main paper shall nominate him/her.

Presentation at various academic associations

The right to present at various academic meetings, excluding first overseas presentation and the first domestic conference, is decided by the Study Representative and the Study Secretariat.

18. References

1. Hesketh PJ, Kris MG, Grunberg Sm, Beck T, Hainsworth JD, Harker G, Apro MS, Grandara D, Lindley CM, Proposal for Classifying the Acute Emetogenicity of Cancer Chemotherapy, J Clin Oncol, 15:103-109(1997)
2. Richardson JL, Kris MG, Levine A, The influence of symptoms of disease and side effects of treatment on compliance with cancer therapy. J Clin Oncol, 6:1746-1752(1988)
3. Japan Society of Clinical Oncology, Guidelines for the proper use of antiemetics, Kanehara, Tokyo. 68(2015)
4. Hesketh JP.Chemotherapy-Induced Nausea and Vomiting,N Engl J Med,358:2482-94(2008)
5. Tonato M, Rolia F, Del Favero A. Methodology of antiemetic trials: a review. Ann Oncol, 2: 107-14(1991)
6. Rolia F, Tonato M, Basurto C, Bella M, Passalacqua R, Marsia D, DiCostanzo F, Donati D, Ballatori E, Tognoni G, Franzosi MG, Del Favero A. Antiemetic Activity of High Dose of Metclopramide combined With Methylprednisolone Versus Metclopramide Alone in Cisplatin-Treated Cancer Patients: A Randomized Double-Blind Trial of Italian Oncology Group for Clinical Research. J Clin Oncol 5: 141-149(1987)
7. Sullivan JR, Lyden MJ, Bell R. Decreased cisplatin-induced nausea and vomiting with chronic alcohol ingestion. N Engl J Med 309: 796(1983)
8. Hesketh JP, Apro M, Street JC, Carides AD. Evaluation of risk factors predictive of nausea and vomiting with current standard-of-care antiemetic treatment: analysis of two phase III trials of aprepitant in patients receiving cisplatin-based chemotherapy. Suport Care Cancer 18: 1171-1177(2010)
9. Suzuki K, Yamanaka T, Hashimoto H, Shimada Y, Arata K, Matsui R, Goto K, Takiguchi T, Ohyanagi F, Kogure Y, Nogami N, Nakao M, Takeda K, Azuma K, Nagase S, Hayashi T, Fujiwara K, Shimada T, Seki N, Yamamoto N. Randomized, double-blind, phase III trial of palonosetron versus granisetron in the triplet regimen for preventing chemotherapy-induced nausea and vomiting after highly emetogenic chemotherapy:
TRIPLE study. Ann Oncol 27:1601-1606 (2016)
10. Aapro M, Fabi A, Medici M, Steger G, Bachmann C, Roncoroni S & Rolia F. Duble-blind, randomized, controlled study of the efficacy and tolerability of palonosetron plus dexamethasone for 1 day with or without dexamethasone on days 2 and 3 in the prevention of nausea and vomiting induced by moderately emetogenic chemotherapy. Ann Oncol 21: 1083-1088(2010)
11. Roscoe AJ, Charles EH, Morrow RG, Mohile GS, Dakhil RS, Wade LJ, Kuebler PJ. Prevention of Delayed Nausea: A University of Rochester cancer Center Community Clinical Oncology Program Study of Patients Receiving Chemotherapy. J Clin Oncol 30: 3389-3395(2012)
12. Gralla RJ, Itri LM, Pisko SE, Squillante AE, Kelsen DP, Braun DW, Bordin LA, Braun TJ, Young CW. Antiemetic efficacy of high-dose metoclopramide: randomized trials with placebo and prochlorperazine in patients with chemotherapy-induced nausea and vomiting. New Engl J Med, 305:905-909(1981).
13. Fozard JR, Mobarok ALI AT, Blockade of neuronal tryptamine receptor by metocloplamide. Eur J Pharmacol, 49(1),109-112(1978)
14. Ioannidis JP, Hesketh PJ, Lau J, Contribution of dexamethasone to control chemotherapy-induced nausea and vomiting: a meta-analysis of randomizes evidence. J Clin Oncol, 18, 3409-22(2000)
15. Parikh PM, Charak BS, Banavali SD, Koppikar SB, Giri N, Nadkarni P, Saikia TK, Gopal R, Advani SH. A prospective, randomized double-blind trial comparing metoclopramide alone with metoclopramide plus dexamethasone in preventing emesis induced by high-dose cisplatin. Cancer 62(10), 2263-6(1988)
16. Shinkai T, Saijo N, Eguchi K. Antiemetic efficacy of high-dose intravenous metoclopramide and dexamethasone in patients receiving cisplatin-based chemotherapy: A randomized controlled trial. Jpn J Clin Oncol 16, 279-287(1986)
17. Strum SB, McDermed JE, Liponi DF. High-Dose Intravenous Metclopramide Versus combination High-Dose Metoclopramide and Intravenous Dexamethazone in preventing Cispletin-Induced Nausea and Emesis: A Single-Blind Crossover Comparison of Antiemetic Efficacy. J Clin Oncol 3(2), 245-251(1985)
18. Hainsworth J, Harvey W, Pendergrass K, Kasimis B, Oblon D, Monaghan G, Gandara D, Hesketh P, Khojasteh A, Harker G, et al. A single-blind comparison of intravenous ondansetron, a selective serotonin antagonist, with intravenous metoclopramide in the prevention of nausea and vomiting associated with high-dose cisplatin chemotherapy. J Clin Oncol. 9(5), 721-8(1991)
19. The Italian Group for Antiemetic Research. Ondansetron versus metoclopramide, both combined with dexamethasone, in the prevention of cisplatin-induced delayed emesis. J Clin Oncol. 15(1), 124-30(1997)
20. Hesketh PJ, Grunberg SM, Gralla RJ, Warr DG, Roila F, de Wit R, Chawla SP, Carides AD, Ianus J, Elmer ME, Evans JK, Beck K, Reines S, Horgan KJ; Aprepitant Protocol 052 Study Group, The oral neurokinin-1 antagonist

aprepitant for the prevention of chemotherapy-induced nausea and vomiting: a multinational, randomized, double-blind, placebo-controlled trial in patients receiving high-dose cisplati—the Aprepitant Protocol 052 Study Group. J Clin Oncol 21(22), 4112-9(2003)

21. Poli-Bigelli S, Rodrigues-Pereira J, Carides AD, Julie Ma G, Eldridge K, Hipple A, Evans JK, Horgan KJ, Lawson F; Aprepitant Protocol 054 Study Group, Addition of the neurokinin 1 receptor antagonist aprepitant to standard antiemetic therapy improves control of chemotherapy-induced nausea and vomiting. Results from a randomized, double-blind, placebo-controlled trial in Latin America. Cancer, 97(12), 3090-8(2003)
22. Chawla SP, Grunberg SM, Gralla RJ, Hesketh PJ, Rittenberg C, Elmer ME, Schmidt C, Taylor A, Carides AD, Evans JK,Horgan KJ; Establishing the Dose of the Oral NK1 Antagonist Aprepitant for the Prevention of Chemotherapy Induced Nausea and Vomiting. Cancer, 97(9), 2290-300(2003)
23. Saito M, Aogi K, Sekine I, Yoshizawa H, Yanagita Y, Sakai H, Inoue K, Kitagawa C, Ogura T, Mitsuhashi S. Palonosetron plus dexamethasone versus granisetron plus dexamethasone for prevention of nausea and vomiting during chemotherapy: a double-blinde, double-dummy, randomized, comparative phase III trial. Lancet Oncol, 10, 115-24(2009)
24. Allison DB, Casey DE. Antipsychotic-induced weight gain: a review of the literature. J Clin Psychiatry. 62, 22-31(2001)
25. Hale AS. Olanzapine. Br J Hosp Med 58, 443-5(1997)
26. Bymaster, F. P. Neuropsychopharmacology, 14, 87(1996)
27. Shotte, A. Psychopharmacology, 124, 57(1996)
28. Bymaster, F. P. Eur. J. Pharmacol, 390, 245(2000)
29. Navari RM, Gray SE, Kerr AC. Olanzapine versus aprepitant for the prevention of chemotherapy-induced nausea and vomiting: a randomized phase III trial. J Support Oncol. 9(5), 188-95(2011)
30. Navari RM, Nagy CK, Gray SE. The use of olanzapine versus metoclopramide for the treatment of breakthrough chemotherapy-induced nausea and vomiting in patients receiving highly emetogenic chemotherapy. Support Care Cancer. 21(6), 1655-63(2013)
31. http://www.nccn.org/professionals/physician_gls/pdf/antiemesis.pdf
32. Navari R, Qin R, Ruddy K, Liu Heshan, Powell FS, Bajaj M, Dietrich LL,Biggs D, Lafky MJ and Loprinzi LC. Olanzapine for the prevention of chemotherapy-induced nausea and vomiting. New Engl J Med 375, 134-142(2016)
33. Abe M, Hirashima Y, Kasamatsu Y, Kado N, Komeda S, Kuji S, Tanaka A, Takahashi N, Takekuma M, Hihara H,Ichikawa Y, Itonaga Y, Hirakawa T, Nasu K, Miyagi K, Murakami J, Ito K. Efficacy and safety of olanzapine combined with aprepitant, palonsetron, and dexamethasone for preventing nausea and vomiting induced by cisplatin-based chemotherapy in gynecological cancer: KCOG-G1301 phase II trial. Support Care Cancer 24: 675-682(2016)
34. Mizukami N, Yamauchi M, Koike K, Watanabe A, Ichihara K, Masumori N, Yamakage M. Olanzapine for the prevention of chemotherapy-induced nausea and vomiting in patients receiving highly or moderately emetogenic chemotherapy: a randomized, double-blind, placebo-contraolled study. J Pain Symptom Manage. 47(3)542-40(2014)
35. Hashimoto H, Yanai T, Nagashima K, Okita N, Horinouchi H, Takiguchi T, Ohyanagi F, Nakao M, Takeda K, Nakayama T, Sakai H, Arai T, Minato K, Kogure Y, Harada D, Suzuki K, Boku N, Shimada Y, Iwasa S, Yamamoto N. A double-blind randomized phaseII study of 10 versus 5 mg olanzapine for emesis induced by highly emetogenic chemotherapy with cisplatin. J Clin Oncol 34, 2016(suppl; abstr 10111)
36. Aapro M, Rugo H, Rossi G, Rizzi G, Borroni ME, Bondarenko I, Sarosiek T, Oprean C, Cardona-Huerta S, Lorusso V,Karthaus M, Schwartzberg L, Grunberg S. A randomized phase III study evaluating the efficacy and safety of NEPA, a fix-dose combination of netupitant and palonosetron, for prevention of chemotherapy-induced nausea and vomiting following moderately emetogenic chemotherapy. Ann Oncol. 25(7):1328-33(214)
37. Barton DL, Thanarajasingam G, Sloan JA, Diekmann B, Fuloria J, Kottschade LA, Lyss AP, Jaslowski AJ, Mazurczak MA, Blair SC, Terstriep S, Loprinzi CL.Phase III double-blind, placebo-controlled study of gabapentin for the prevention of delayed chemotherapy-induced nausea and vomiting in patients receiving highly emetogenic chemotherapy, NCCTG N08C3(Alliance).Cancer, 120(22): 3575-83(2014)
38. Rolia F, Tonato M, basurto C, Picciafuoco M, Bracarda S, Donati D, Malacarne P, Monici L, Costanzo FD, Patoia L, Ballatori G, Favero AD. Protection from Nausea and Vomiting in Cisplatin-Treated Ptients: High-Dose Metoclopramide Combined With Dexamethasone and Diphenhydramine: A Study of the Italian Oncology Group for Clinical research. J Clin Oncol. 7(11): 1693-1700(1989)
39. Sekine I, Segawa Y, Kubota K, Saeki T. Risk factors of chemotherapy-induced nausea and vomiting: Index for

personalized antiemetic prophylaxis. Cancer Sci 104(6): 711-7(2013)

40. Rolia F, Tonato M, Basurto C. Antiemetic activity of two different high dose of metoclopramide in cisplatin –treated cancer patients: a randomized double-blind trial of the Italian Oncology Group for Clinical Research. Cancer Treat rep 69: 1353-7(1985)

J-SUPPORT AND FORTH AGENT OLANZAPINE RESIST CISPLATIN EMETOGENESITY (J-FORCE) STATISTICAL ANALYSIS PLAN

Study Number: J-SUPPORT1604

Version: 1.0

Date: 20-12-2018

Principal Investigator:

Masakazu Abe, M.D.
Chief physician
Department of Gynecologic Oncology,
Shizuoka Cancer Center
1007 Shimonagakubo, Nagaizumi-cho,
Sunto-gun, Shizuoka Prefecture 411-8777
Japan
Phone: +81-55-989-5222 Ext. 6202
Fax: +81-55-989-5551
ma.abe@scchr.jp

Hironobu Hashimoto,
Assistant Director

Department of Pharmacy,
National Cancer Center Hospital
Tsukiji 5-1-1, Chuo-ku, Tokyo, 104-0045,
Phone: +81-3-3542-2511 Ext. 3531
Fax: +81-3-3248-0730
hhashimo@ncc.go.jp

Trial Statistician:

Takuhiro Yamaguchi, Ph.D.
Professor and Director

Biostatistics, Tohoku University Graduate School of Medicine
Clinical Research Data Center, Tohoku University Hospital
1-1, Seiryo-machi, Aoba-ku, Sendai, Miyagi 980-8574, JAPAN
Phone: +81-22-717-7659
Fax: +81-22-717-7580
Email: yamaguchi@med.tohoku.ac.jp

STATISTICAL ANALYSIS PLAN

Signature of Agreement

I have read this statistical analysis plan and agree to conduct the analysis as outlined herein:

______________________________ ______________

Masakazu Abe, M.D. Date
Shizuoka Cancer Center Hospital

______________________________ ______________

Hironobu Hashimoto, Date
National Cancer Center Hospital

______________________________ ______________

Takuhiro Yamaguchi, Ph.D. Date
Tohoku University Graduate School of Medicine

CONTENTS

1. SCOPE ... 7
2. BACKGROUND of J-FORCE Study ... 8
3. GENERAL CONSIDERATIONS ... 11
3.1 Software ... 11
3.2 Significance Level and Reliability Coefficient ... 11
3.3 Numerical Display of Results ... 11
4. DATA HANDLING AND ANALYSIS SETS ... 12
4.1 Data Handling ... 12
4.2 Analysis Sets ... 12
5. DESCPRITIVE ANALYSES ... 13
5.1 Patient Flow and Reliability of Trial ... 13
5.2 Baseline Comparability of Treatment Groups ... 13
6. EFFICACY ANALYSES ... 15
6.1 Primary Efficacy Analyses ... 15
6.2 Secondary Efficacy Analyses ... 15
6.2.1 CR Rate in the Acute and the Overall Phase ... 15
6.2.2 CR Rate Every 24 Hours in the Overall Phase ... 16
6.2.3 CR Rate from Day1 to 5 ... 18
6.2.4 CC Rate from Day1 to 5 ... 18
6.2.5 TC Rate from Day1 to 5 ... 18
6.2.6 Subgroup Analysis for CR Rate in the Delayed Phase ... 19
6.2.7 Subgroup Analysis for CC Rate in the Overall Phase ... 20
6.2.8 Subgroup Analysis for TC Rate in the Overall Phase ... 20
6.2.9 Patient Reported Nausea from Day1 to 5 ... 21
6.2.10 Patient Reported Vomiting from Day1 to 5 ... 21
6.2.11 Patient Reported Rescue from Day1 to 5 ... 22
6.2.12 Treatment Failure ... 24
6.2.13 Subgroup Analysis for Treatment Failure ... 25
6.2.14 Patient-Reported Symptom in Daily Life from Day1 to 5 ... 26
6.2.15 Patient Satisfaction ... 28
6.2.16 Numbers of Episodes of Vomiting and Nausea in the Overall Phase ... 29
6.2.17 Risk Factors for Delayed Vomiting and Nausea ... 30
7. SAFETY ANALYSES ... 33
7.1 Adverse Events ... 33

7.1.1 Distribution of Adverse Events between Groups 33
7.1.2 Subgroup Analysis for Distribution of Adverse Events between Groups 33
7.1.3 Relationship between Somnolence and Nausea Grading 34
7.1.4 Sleepiness in the overall phase 34
8. REVISION HISTORY 37

ABBREVIATIONS AND DEFINITIONS

APR	Aprepitant
CDDP	Cisplatin
CTCAE	Common Terminology Criteria for Adverse Events
DEX	Dexamethasone
ECOG	Eastern Cooperative Oncology Group
GEE	Generalized Estimating Equation
OLZ	Olanzapine
PALO	Palonosetron
y.o.	years old
5-HT3-RA	5-hydroxytryptamine 3 receptor antagonists

1. SCOPE

This document gives a detailed statistical analysis plan for the J-FORCE trial, and should be read in conjunction with the current trial protocol. Portions of this document are replicated from the study protocol, and supplemented with additional detail as appropriate.

2. BACKGROUND of J-FORCE Study

Objective

The objective of this study is to confirm the efficacy of olanzapine (5 mg) combined with aprepitant, palonosetron, and dexamethasone for prevention of chemotherapy induced nausea and vomiting in patients receiving cisplatin-based highly emetogenic chemotherapy.

Study setting

This study is a multicenter, randomized, double-blind, placebo-controlled Phase III study.

Resources

This study is supported by the Japan Supportive, Palliative and Psychosocial Oncology Group (J-SUPPORT) and funded by the Japan Agency for Medical Research and Development (AMED).

Endpoints

The primary endpoint is the CR (complete response = no vomiting and no use of rescue medications) rate in the delayed phase after chemotherapy (24–120 h). The secondary endpoints are the CR rate in the acute phase (0–24 h); the overall phase (0–120 h); the CC (complete control = no vomiting, no use of rescue medications, and no or mild nausea) rate and the TC (total control = no vomiting, no use of rescue medications, and no nausea) rate in the acute phase, delayed phase, and overall; and the time to treatment failure (defined as the time from cisplatin administration to an episode of vomiting or the use of rescue medication). We evaluated endpoints based on the patients' self-written daily records. Patients kept a daily record of emetic episodes, use of rescue medication and the presence of nausea and its severity graded on a four category scale. An emetic episode was defined as one or more occurrences of vomiting or retching. Safety is assessed according to CTCAE v4.0. Nausea, anorexia, and sleepiness as efficacy endpoints are classified into study specific four categories (none, mild, moderate or severe).

Eligibility criteria

Inclusion criteria

Patients are required to fulfill all of the following criteria for inclusion in this study:

Solid malignant tumor.

No prior cisplatin-based highly emetogenic chemotherapy.

Scheduled for treatment with cisplatin at ≥50 mg/m^2.

ECOG performance status of 0 to 2.
Aged 20–75 years.
Adequate organ function.
Written informed consent.
Able to understand and describe patient-reported outcomes.

Exclusion criteria
Patients are excluded if they meet any of the following criteria:
A history of hypersensitivity or allergy to any of the study drugs or similar compounds.
Current treatment for nausea or vomiting.
Symptomatic brain metastases.
Pregnant or breastfeeding women and women of childbearing potential, as well as men wishing to father children.
Patients scheduled for abdominal or pelvic radiotherapy.
Patients requiring treatment for ascites.
Gastrointestinal obstruction.
Severe complications (pulmonary fibrosis, heart failure, myocardial infarction, unstable angina, cerebrovascular disorder, psychiatric disease, renal dysfunction, liver dysfunction, intestinal paresis, ileus, uncontrolled diabetes mellitus, active peptic ulcer, etc.).
Patients on anticonvulsant therapy.
Diabetes mellitus with use of antidiabetic agents and HbA1c (NGSP) ≥6.5% or HbA1c (JDS) ≥6.1%.
A history of using any of the following drugs within 48 h before enrollment: opioids, aprepitant, 5-HT3-RA, dexamethasone, dopamine receptor antagonists, antihistamines, benzodiazepines or phenothiazine antipsychotic agents.
Habitual smoking.

<u>Randomization</u>
After confirming fulfillment of the eligibility criteria, the patient is registered by an independent central registration system. Patients are randomized 1:1 to either olanzapine (5 mg) or placebo by minimization method adjusting gender (male vs. female), the planned dose of cisplatin (≥70 mg/m^2 vs. <70 mg/m^2), and the age (≥55 vs. <55 years).

<u>Treatment</u>

The study drug is Swedish orange capsules for double-blind clinical trials filled with 1% olanzapine granules or lactose. Olanzapine capsules contain 0.25 g of 1% Zyprexa® granule per capsule, while placebo capsules contain 0.25 g of lactose per capsule. Patients receiving highly emetogenic chemotherapy are given prophylactic treatment with olanzapine (5 mg) or placebo plus standard triple antiemetic therapy (a combination of aprepitant, palonosetron and dexamethasone). Patients will take two capsules of olanzapine (2.5 mg) orally after dinner on Days 1–4. There are no specific recommendations regarding antiemetic therapy after the completion of protocol treatment, so prophylactic use of olanzapine is permissible.

Follow-up

Physical examination, safety evaluations and laboratory tests are performed before initiation of treatment. Laboratory tests are performed once between Days 5 and 10. After the end of the study period on Day 6, patient-reported outcomes are collected.

Study design and statistical analysis

This study is a randomized double-blind, placebo-controlled Phase III study that is designed to evaluate the efficacy of add-on therapy with olanzapine at 5 mg/day. If the CR rate is significantly higher than that in the placebo group, olanzapine (5 mg) combined with aprepitant, 5HT3-RA and dexamethasone will have the potential to become a standard antiemetic regimen for patients receiving highly emetogenic chemotherapy. A delayed phase CR of 65% is considered to be unacceptable with standard treatment (aprepitant, palonosetron and dexamethasone). The required sample size was calculated to be 345 patients per arm for the study to have a power of 80%, assuming that the CR rate will be 75% in the olanzapine group and 65% in the placebo group, and also assuming a one-sided significance level of 0.025 and 5% ineligible cases. Point estimates and confidence intervals for the CR rate will be calculated and will be compared between groups by using the Mantel-Haenszel test with adjustment for allocation factors.

3. GENERAL CONSIDERATIONS

3.1 Software

Statistical Analysis System (SAS) Ver.9.4. will be used for all statistical analysis.

3.2 Significance Level and Reliability Coefficient

Unless otherwise specified, estimates of treatment effects will be presented with 95% confidence intervals. P-values will be 1-tailed for the primary efficacy analysis (see 6.1) and 2-tailed for other analyses. No correction for multiple tests will be made.

3.3 Numerical Display of Results

As a general rule, the analysis result is indicated by the number of digits shown below. The data is rounded to the nearest whole number then displayed.

Min, Max	Number of significant digits of data
Mean, Quantile	Number of significant digits of data plus one
Standard Deviation (SD)	Number of significant digits of data plus two
Percent (%)	One decimal place
p-value	Three digits after the decimal point or <0.001 (if less than 0.001)

4. DATA HANDLING AND ANALYSIS SETS

4.1 Data Handling

Regarding the handling of individual cases and data, follow the results of examination at the data review meeting.

4.2 Analysis Sets

For efficacy analyses, all randomized subjects who satisfied eligibility criteria, received dose of cisplatin >=50 mg/m^2 and at least one study treatment will be included. For safety analyses, all subjects who received any study treatment will be used.

5. DESCPRITIVE ANALYSES

5.1 Patient Flow and Reliability of Trial

1） Purpose: To describe the progress in each group, that is, enrolment, intervention allocation, follow-up and data analysis.

2） Analysis set: All randomized subjects.

3） Endpoint: Reason for off-treatment and exclusion from analysis.

4） Analysis method: A detailed CONSORT diagram describing subject flow with exclusions and total numbers randomized to each treatment. Frequency of withdrawal / dropout and those reasons are aggregated for each treatment group. Also frequency of inclusion / exclusion from analysis and the reason for exclusion will be summarized.

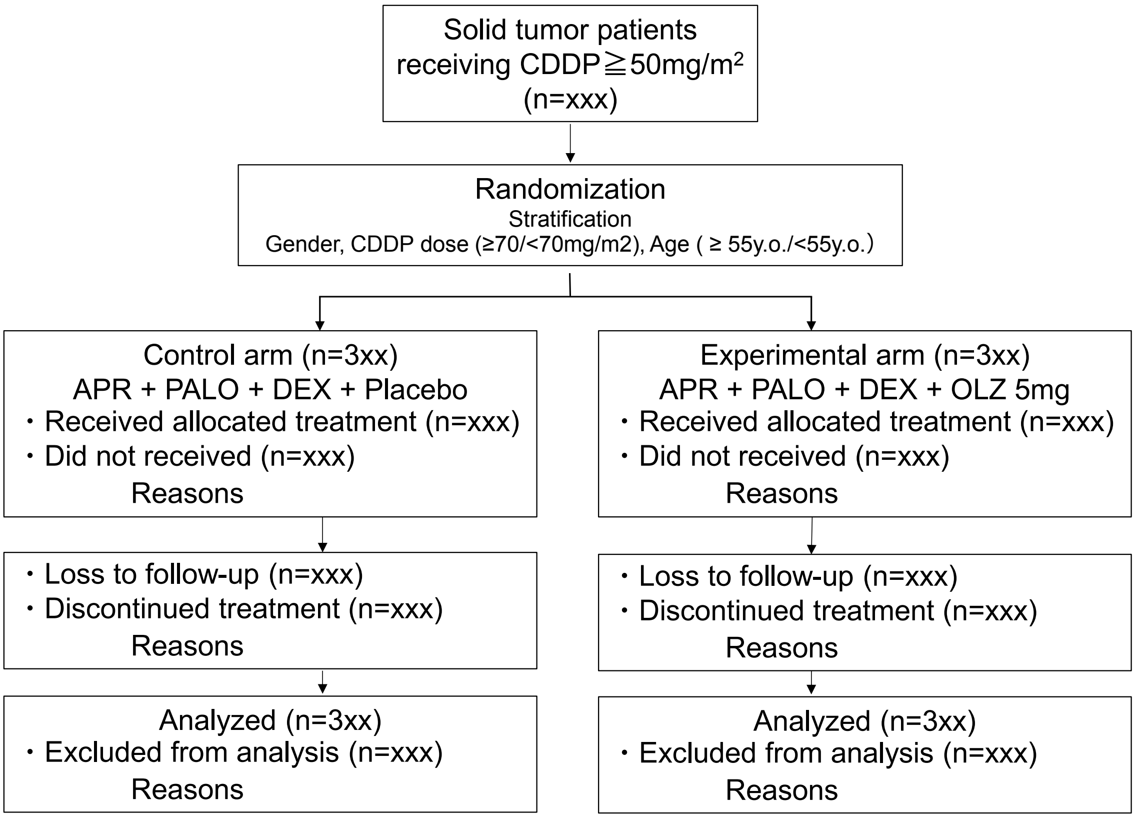

Figure. CONSORT Diagram

5.2 Baseline Comparability of Treatment Groups

1） Purpose: To describe demographic variables and patient characteristics (data before protocol treatment start) to grasp subject background and examine comparability among groups.

2） Analysis set: All randomized subjects.

3） Endpoint: Age, Sex, ECOG Performance Status (PS), Cancer Type, Dose of Cisplatin

4） Analysis method: Tables of summary statistics will be produced by treatment group for a number of baseline variables; age (continuous), sex, ECOG PS (ordinal), cancer type (nominal), Dose of Cisplatin (binary, ≥<70mg/m^2). Continuous variables will be summarized using the following statistics; n (non-missing sample size), mean, standard deviation, median, minimum, and maximum. The number of missing observations will also be reported. The frequency and percentages (based on the non-missing sample size) of observed levels will be reported for all categorical measures. The number of missing observations will also be reported.

Table. Baseline Characteristics

Characteristic	Olanzapine (n=xxx)	Placebo (n=xxx)	p value
**Age**			
median	xx	xx	
range	xx-xx	xx-xx	
≥ 55y.o.	xx (xx)	xx (xx)	.xxx
< 55y.o.	xx (xx)	xx (xx)	
**Sex**			
Male	xx (xx)	xx (xx)	
Female	xx (xx)	xx (xx)	
**ECOG performance status**			.xxx
0	xx (xx)	xx (xx)	
1	xx (xx)	xx (xx)	
2	xx (xx)	xx (xx)	
**Cancer type**			.xxx
Head and neck	xx (xx)	xx (xx)	
Lung	xx (xx)	xx (xx)	
Esophageal	xx (xx)	xx (xx)	
Gynecological	xx (xx)	xx (xx)	
Urological	xx (xx)	xx (xx)	
Other	xx (xx)	xx (xx)	
**Dose of cisplatin**			.xxx
≥70mg/m^2	xx (xx)	xx (xx)	
<70mg/m^2	xx (xx)	xx (xx)	
**Conccurrent radiotherapy**			.xxx
Yes	xx (xx)	xx (xx)	
No	xx (xx)	xx (xx)	

6. EFFICACY ANALYSES

6.1 Primary Efficacy Analyses

1) Purpose: To confirm the superiority of olanzapine at 5mg combined with standard triple antiemetic therapy using aprepitant, palonosetron and dexamethasone in patients receiving highly emetogenic chemotherapy. The primary endpoint is the CR rate in the delayed phase after chemotherapy (24–120 h).
2) Analysis set: All randomized subjects who satisfied eligibility criteria, received dose of cisplatin >=50 mg/m^2 and at least one study treatment (Efficacy Analysis Set).
3) Endpoint: CR rate in the delayed phase after chemotherapy (24-120h).
4) Analysis Methods : Point estimates and confidence intervals for the CR rate in the delayed phase will be calculated in each group and will be compared between groups using the Mantel-Haenszel test with adjustment for allocation factors (gender (male vs. female), the planned dose of cisplatin (≥70 mg/m^2 vs. <70 mg/m^2), and the age (≥55 vs. <55 years). When the number of subjects in each strata is small, the handling of allocation adjustment factors is determined in this analysis plan. Mantel-Haenszel estimate of the common risk difference and stratified Newcombe confidence limits will be calculated.

6.2 Secondary Efficacy Analyses

For the secondary efficacy analyses, adjustment with the allocation factors will not be performed in principle. Also multiplicity adjustment will not be considered.

6.2.1 CR Rate in the Acute and the Overall Phase

1) Purpose: To estimate and compare the CR rate in the acute and the overall phase between groups.
2) Analysis set: Efficacy Analysis Set
3) Endpoint: CR rate in the acute phase (0-24h) and the overall phase (0-120h) after chemotherapy.
4) Analysis method: Point estimates and confidence intervals for the CR rate will be calculated in each group and will be compared between groups using the Mantel Haenszel test as is used in 6.1 4).

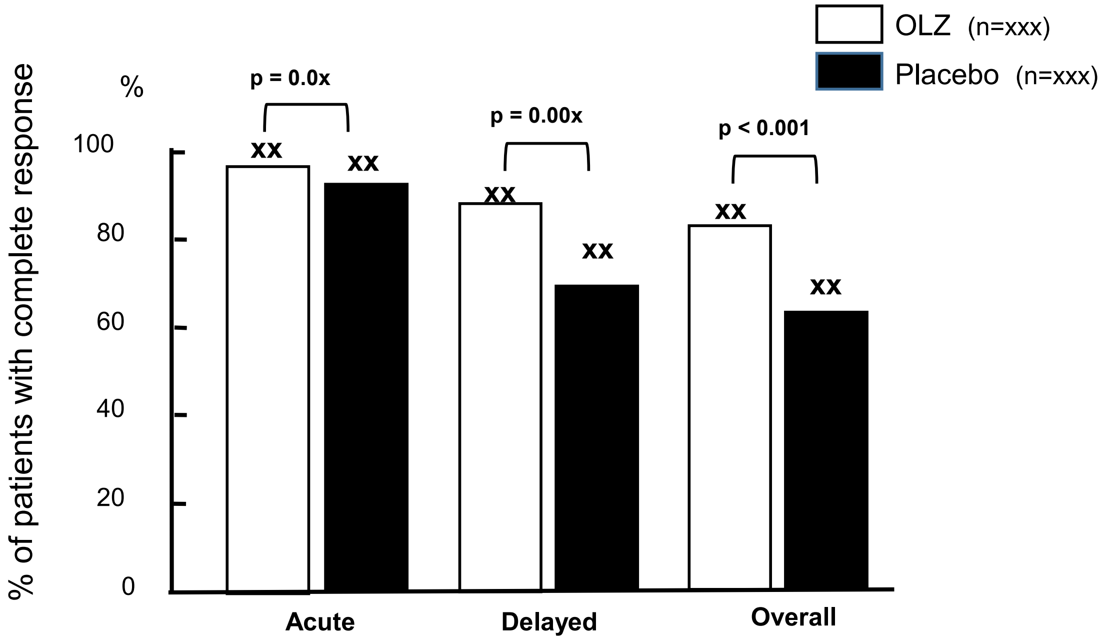

Figure. Complete Response Rate

Table. CR, CC, and TC Rate

Outcome	Olanzapine (n=xxx)		Placebo (n=xxx)		Difference		p value
	N (%)	95% CI	N (%)	95% CI	Estimate	95% CI	
**CR**							
**Acute**	xx (xx%)	xx.x-xx.x	xx (xx%)	xx.x-xx.x	xx%	xx.x-xx.x	.00x
**Delayed**	xx (xx%)	xx.x-xx.x	xx (xx%)	xx.x-xx.x	xx%	xx.x-xx.x	.00x
**Overall**	xx (xx%)	xx.x-xx.x	xx (xx%)	xx.x-xx.x	xx%	xx.x-xx.x	.00x
**CC**							
**Acute**	xx (xx%)	xx.x-xx.x	xx (xx%)	xx.x-xx.x	xx%	xx.x-xx.x	.00x
**Delayed**	xx (xx%)	xx.x-xx.x	xx (xx%)	xx.x-xx.x	xx%	xx.x-xx.x	.00x
**Overall**	xx (xx%)	xx.x-xx.x	xx (xx%)	xx.x-xx.x	xx%	xx.x-xx.x	.00x
**TC**							
**Acute**	xx (xx%)	xx.x-xx.x	xx (xx%)	xx.x-xx.x	xx%	xx.x-xx.x	.00x
**Delayed**	xx (xx%)	xx.x-xx.x	xx (xx%)	xx.x-xx.x	xx%	xx.x-xx.x	.00x
**Overall**	xx (xx%)	xx.x-xx.x	xx (xx%)	xx.x-xx.x	xx%	xx.x-xx.x	.00x

6.2.2 CR Rate Every 24 Hours in the Overall Phase

1） Purpose: To estimate and compare the CR rate every 24 hours in the overall phase

between groups.

2） Analysis set: Efficacy Analysis Set

3） Endpoint: CR rate in 0-24h (day1), 24-48h (day2), 48-72 (day3), 72-96 (day4) and 96-120 (day5) hours after chemotherapy.

4） Analysis method: The proportion of complete response will be estimated with risk difference model using GEE with unstructured/exchangeable working correlation structures to examine treatment by time interaction. Point estimates and confidence intervals for the CR rate will be calculated in each group and will be compared between groups for each time.

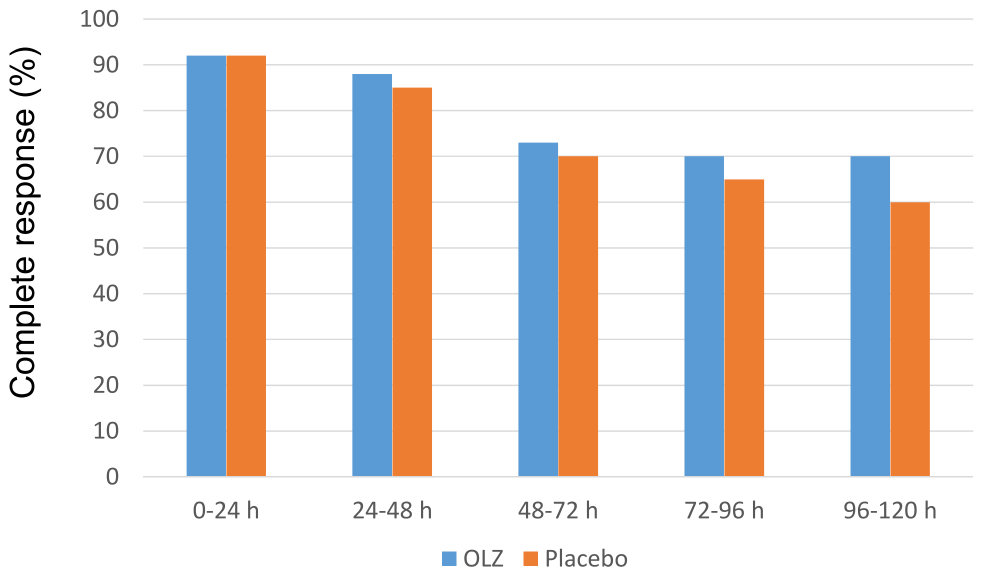

Figure. Time Course of Complete Response (by 24-h Periods)

Table. CR Rate Every 24 Hours in the Overall Phase

Outcome	Olanzapine (n=xxx)		Placebo (n=xxx)		Difference		p value
	N (%)	95% CI	N (%)	95% CI	Estimate	95% CI	
**CR**							
**Acute (0-24h)**	xx (xx%)	xx.x-xx.x	xx (xx%)	xx.x-xx.x	xx%	xx.x-xx.x	.00x
**Delayed**	xx (xx%)	xx.x-xx.x	xx (xx%)	xx.x-xx.x	xx%	xx.x-xx.x	.00x
**- 48h**	xx (xx%)	xx.x-xx.x	xx (xx%)	xx.x-xx.x	xx%	xx.x-xx.x	.00x
**- 72h**	xx (xx%)	xx.x-xx.x	xx (xx%)	xx.x-xx.x	xx%	xx.x-xx.x	.00x
**- 96h**	xx (xx%)	xx.x-xx.x	xx (xx%)	xx.x-xx.x	xx%	xx.x-xx.x	.00x
**-120h**	xx (xx%)	xx.x-xx.x	xx (xx%)	xx.x-xx.x	xx%	xx.x-xx.x	.00x

6.2.3 CR Rate from Day1 to 5

1） Purpose: To estimate and compare the CR rate from day1 to 5 between groups.

2） Analysis set: Efficacy Analysis Set

3） Endpoint: CR rate in day1, 2, 3, 4 and 5 after chemotherapy.

4） Analysis method: The same as Section 6.2.2. 4).

6.2.4 CC Rate from Day1 to 5

1） Purpose: To estimate and compare the CC rate from day1 to 5 between groups.

2） Analysis set: Efficacy Analysis Set

3） Endpoint: CC rate in day1, 2, 3, 4 and 5 after chemotherapy.

4） Analysis method: The same as Section 6.2.2. 4).

6.2.5 TC Rate from Day1 to 5

1） Purpose: To estimate and compare the TC rate from day1 to 5 between groups.

2） Analysis set: Efficacy Analysis Set

3） Endpoint: TC rate in day1, 2, 3, 4 and 5 after chemotherapy.

4） Analysis method: The same as Section 6.2.2. 4).

Table. CR, CC, and TC Rate From Day 1 to 5

	Olanzapine	Placebo	p value
**CR**			.xxx
Day 1	xx (xx%)	xx (xx%)	.xxx
Day 2	xx (xx%)	xx (xx%)	.xxx
Day 3	xx (xx%)	xx (xx%)	.xxx
Day 4	xx (xx%)	xx (xx%)	.xxx
Day 5	xx (xx%)	xx (xx%)	.xxx
**CC**			.xxx
Day 1	xx (xx%)	xx (xx%)	.xxx
Day 2	xx (xx%)	xx (xx%)	.xxx
Day 3	xx (xx%)	xx (xx%)	.xxx
Day 4	xx (xx%)	xx (xx%)	.xxx
Day 5	xx (xx%)	xx (xx%)	.xxx
**TC**			.xxx
Day 1	xx (xx%)	xx (xx%)	.xxx
Day 2	xx (xx%)	xx (xx%)	.xxx
Day 3	xx (xx%)	xx (xx%)	.xxx
Day 4	xx (xx%)	xx (xx%)	.xxx
Day 5	xx (xx%)	xx (xx%)	.xxx

6.2.6 Subgroup Analysis for CR Rate in the Delayed Phase

1） Purpose: To estimate and compare the CR rate in the delayed phase between treatment groups according to subgroups.

2） Analysis set: Efficacy Analysis Set

3） Endpoint: CR rate in the delayed phase after chemotherapy.

4） Analysis method: Point estimates and confidence intervals for the CR rate difference between treatment groups will be calculated according to subgroups (gender (male vs. female), age (<55 vs. >=55 years), ECOG PS (0 vs. 1-2), primary site (esophageal, gastric, lung, head and neck, gynecological, others), CDDP dose (<70 vs. >=70mg/m^2)).

6.2.7 Subgroup Analysis for CC Rate in the Overall Phase

1) Purpose: To estimate and compare the CC rate in the overall phase between treatment groups according to subgroups.
2) Analysis set: Efficacy Analysis Set
3) Endpoint: CC rate in the overall phase after chemotherapy.
4) Analysis method: Point estimates and confidence intervals for the CC rate difference between treatment groups will be calculated according to subgroups (gender (male vs. female), age (<55 vs. >=55 years), ECOG PS (0 vs. 1-2), primary site (esophageal, gastric, lung, head and neck, gynecological, others), CDDP dose (<70 vs. >=70mg/m^2), drinking habit (yes vs. no), motion sickness (yes vs. no), pregnancy sickness (yes vs. no),).

6.2.8 Subgroup Analysis for TC Rate in the Overall Phase

5) Purpose: To estimate and compare the TC rate in the overall phase between treatment groups according to subgroups.
6) Analysis set: Efficacy Analysis Set
7) Endpoint: TC rate in the overall phase after chemotherapy.
8) Analysis method: Point estimates and confidence intervals for the TC rate difference between treatment groups will be calculated according to subgroups (gender (male vs. female), age (<55 vs. >=55 years), ECOG PS (0 vs. 1-2), primary site (esophageal, gastric, lung, head and neck, gynecological, others), CDDP dose (<70 vs. >=70mg/m^2), drinking habit (yes vs. no), motion sickness (yes vs. no), pregnancy sickness (yes vs. no),).

Table. CR, CC, and TC Rate according to …

Outcome	Olanzapine (n=xxx)		Placebo (n=xxx)		Difference		p value
	N (%)	95% CI	N (%)	95% CI	Estimate	95% CI	
**CR**							
**Acute**	xx (xx%)	xx.x-xx.x	xx (xx%)	xx.x-xx.x	xx%	xx.x-xx.x	.00x
**Delayed**	xx (xx%)	xx.x-xx.x	xx (xx%)	xx.x-xx.x	xx%	xx.x-xx.x	.00x
**Overall**	xx (xx%)	xx.x-xx.x	xx (xx%)	xx.x-xx.x	xx%	xx.x-xx.x	.00x
**CC**							
**Acute**	xx (xx%)	xx.x-xx.x	xx (xx%)	xx.x-xx.x	xx%	xx.x-xx.x	.00x
**Delayed**	xx (xx%)	xx.x-xx.x	xx (xx%)	xx.x-xx.x	xx%	xx.x-xx.x	.00x
**Overall**	xx (xx%)	xx.x-xx.x	xx (xx%)	xx.x-xx.x	xx%	xx.x-xx.x	.00x
**TC**							
**Acute**	xx (xx%)	xx.x-xx.x	xx (xx%)	xx.x-xx.x	xx%	xx.x-xx.x	.00x
**Delayed**	xx (xx%)	xx.x-xx.x	xx (xx%)	xx.x-xx.x	xx%	xx.x-xx.x	.00x
**Overall**	xx (xx%)	xx.x-xx.x	xx (xx%)	xx.x-xx.x	xx%	xx.x-xx.x	.00x

6.2.9 Patient Reported Nausea from Day1 to 5

1) Purpose: To estimate and compare the distribution of patient reported nausea from day1 to 5 between groups.
2) Analysis set: Efficacy Analysis Set
3) Endpoint: Patient reported nausea in day1, 2, 3, 4 and 5 after chemotherapy.
4) Analysis method: Distribution of patient reported nausea in the day 1, 2, 3, 4 and 5 will be estimated in each group and will be compared between groups using the Mantel test. In addition, the proportion of subjects with response “None” will be estimated with risk difference model using GEE with unstructured/exchangeable working correlation structures to examine treatment by time interaction. Point estimates and confidence intervals for the proportion will be calculated in each group and will be compared between groups for each time. The proportion of subjects with response “None” or “Mild” will be estimated as well.

6.2.10 Patient Reported Vomiting from Day1 to 5

1) Purpose: To estimate and compare the distribution of patient reported vomiting from day1 to 5 between groups.
2) Analysis set: Efficacy Analysis Set
3) Endpoint: Patient reported vomiting in day1, 2, 3, 4 and 5 after chemotherapy.
4) Analysis method: The same as Section 6.2.8 4)

6.2.11 Patient Reported Rescue from Day1 to 5

1） Purpose: To estimate and compare the distribution of patient reported rescue from day1 to 5 between groups.

2） Analysis set: Efficacy Analysis Set

3） Endpoint: Patient reported rescue in day1, 2, 3, 4 and 5 after chemotherapy.

4） Analysis method: The same as Section 6.2.8 4)

Table. Incidences of Patient-Reported Nausea, Vomiting and Rescue From Day 1 to 5

	Olanzapine				Placebo				p value
**Nausea**	None	Mild	Moderate	Severe	None	Mild	Moderate	Severe	
Day 1	xx (xx%)	xx (xx%)	xx (xx%)	xx (xx%)	xx (xx%)	xx (xx%)	xx (xx%)	xx (xx%)	.xxx
Day 2	xx (xx%)	xx (xx%)	xx (xx%)	xx (xx%)	xx (xx%)	xx (xx%)	xx (xx%)	xx (xx%)	.xxx
Day 3	xx (xx%)	xx (xx%)	xx (xx%)	xx (xx%)	xx (xx%)	xx (xx%)	xx (xx%)	xx (xx%)	.xxx
Day 4	xx (xx%)	xx (xx%)	xx (xx%)	xx (xx%)	xx (xx%)	xx (xx%)	xx (xx%)	xx (xx%)	.xxx
Day 5	xx (xx%)	xx (xx%)	xx (xx%)	xx (xx%)	xx (xx%)	xx (xx%)	xx (xx%)	xx (xx%)	.xxx
**Vomiting**	None	1	2	≥ 3	None	1	2	≥ 3	
Day 1	xx (xx%)	xx (xx%)	xx (xx%)	xx (xx%)	xx (xx%)	xx (xx%)	xx (xx%)	xx (xx%)	.xxx
Day 2	xx (xx%)	xx (xx%)	xx (xx%)	xx (xx%)	xx (xx%)	xx (xx%)	xx (xx%)	xx (xx%)	.xxx
Day 3	xx (xx%)	xx (xx%)	xx (xx%)	xx (xx%)	xx (xx%)	xx (xx%)	xx (xx%)	xx (xx%)	.xxx
Day 4	xx (xx%)	xx (xx%)	xx (xx%)	xx (xx%)	xx (xx%)	xx (xx%)	xx (xx%)	xx (xx%)	.xxx
Day 5	xx (xx%)	xx (xx%)	xx (xx%)	xx (xx%)	xx (xx%)	xx (xx%)	xx (xx%)	xx (xx%)	.xxx
**Rescue**	None	1	2	≥ 3	None	1	2	≥ 3	
Day 1	xx (xx%)	xx (xx%)	xx (xx%)	xx (xx%)	xx (xx%)	xx (xx%)	xx (xx%)	xx (xx%)	.xxx
Day 2	xx (xx%)	xx (xx%)	xx (xx%)	xx (xx%)	xx (xx%)	xx (xx%)	xx (xx%)	xx (xx%)	.xxx
Day 3	xx (xx%)	xx (xx%)	xx (xx%)	xx (xx%)	xx (xx%)	xx (xx%)	xx (xx%)	xx (xx%)	.xxx
Day 4	xx (xx%)	xx (xx%)	xx (xx%)	xx (xx%)	xx (xx%)	xx (xx%)	xx (xx%)	xx (xx%)	.xxx
Day 5	xx (xx%)	xx (xx%)	xx (xx%)	xx (xx%)	xx (xx%)	xx (xx%)	xx (xx%)	xx (xx%)	.xxx

Table. Distribution of NO Patient-Reported Nausea, Vomiting and Rescue From Day 1 to 5

	Olanzapine	Placebo	p value
**Nausea**			.xxx
Day 1	xx (xx%)	xx (xx%)	.xxx
Day 2	xx (xx%)	xx (xx%)	.xxx
Day 3	xx (xx%)	xx (xx%)	.xxx
Day 4	xx (xx%)	xx (xx%)	.xxx
Day 5	xx (xx%)	xx (xx%)	.xxx
**Vomiting**			.xxx
Day 1	xx (xx%)	xx (xx%)	.xxx
Day 2	xx (xx%)	xx (xx%)	.xxx
Day 3	xx (xx%)	xx (xx%)	.xxx
Day 4	xx (xx%)	xx (xx%)	.xxx
Day 5	xx (xx%)	xx (xx%)	.xxx
**Rescue**			.xxx
Day 1	xx (xx%)	xx (xx%)	.xxx
Day 2	xx (xx%)	xx (xx%)	.xxx
Day 3	xx (xx%)	xx (xx%)	.xxx
Day 4	xx (xx%)	xx (xx%)	.xxx
Day 5	xx (xx%)	xx (xx%)	.xxx

Table. Distribution of “None” or “Mild” Patient-Reported Nausea, Vomiting and Rescue From Day 1 to 5

	Olanzapine	Placebo	p value
**Nausea**			.xxx
Day 1	xx (xx%)	xx (xx%)	.xxx
Day 2	xx (xx%)	xx (xx%)	.xxx
Day 3	xx (xx%)	xx (xx%)	.xxx
Day 4	xx (xx%)	xx (xx%)	.xxx
Day 5	xx (xx%)	xx (xx%)	.xxx
**Vomiting**			.xxx
Day 1	xx (xx%)	xx (xx%)	.xxx
Day 2	xx (xx%)	xx (xx%)	.xxx
Day 3	xx (xx%)	xx (xx%)	.xxx
Day 4	xx (xx%)	xx (xx%)	.xxx
Day 5	xx (xx%)	xx (xx%)	.xxx
**Rescue**			.xxx
Day 1	xx (xx%)	xx (xx%)	.xxx
Day 2	xx (xx%)	xx (xx%)	.xxx
Day 3	xx (xx%)	xx (xx%)	.xxx
Day 4	xx (xx%)	xx (xx%)	.xxx
Day 5	xx (xx%)	xx (xx%)	.xxx

6.2.12 Treatment Failure

1) Purpose: To estimate and compare the distribution of time to treatment failure
2) Analysis set: Efficacy Analysis Set
3) Endpoint: Time to treatment failure from Cisplatin administration.
4) Analysis method: Distribution of time to treatment failure will be estimated in each group using Kaplan-Meier method and will be compared between groups using log-rank test.

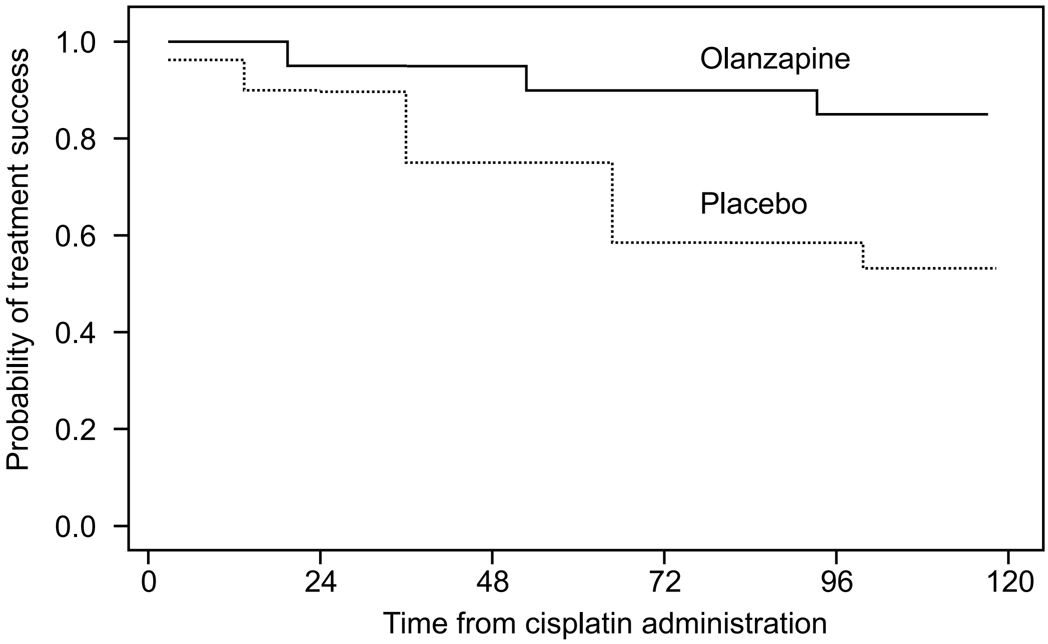

No. at Risk

Olanzapine	XXX	XXX	XXX	XXX	XXX	XXX
Placebo	XXX	XXX	XXX	XXX	XXX	XXX

Figure. Kaplan–Meier Plot of the Time to Treatment Failure

6.2.13 Subgroup Analysis for Treatment Failure

1） Purpose: To estimate and compare the distribution of time to treatment failure

2） Analysis set: Efficacy Analysis Set

3） Endpoint: Time to treatment failure from Cisplatin administration.

4） Analysis method: Distribution of time to treatment failure will be estimated in each combination of treatment groups with several risk factors; gender (male vs. female), age (<55 vs. >=55 years), drinking habit (yes vs. no), motion sickness (yes vs. no), pregnancy sickness (yes vs. no), CDDP dose (<70 vs. >=70mg/m^2), occurrence of vomiting in acute phase (yes vs. no), number of having these six risk factors, occurrence of nausea in acute phase (yes vs. no), using Kaplan-Meier method and will be compared between those groups using log-rank test.

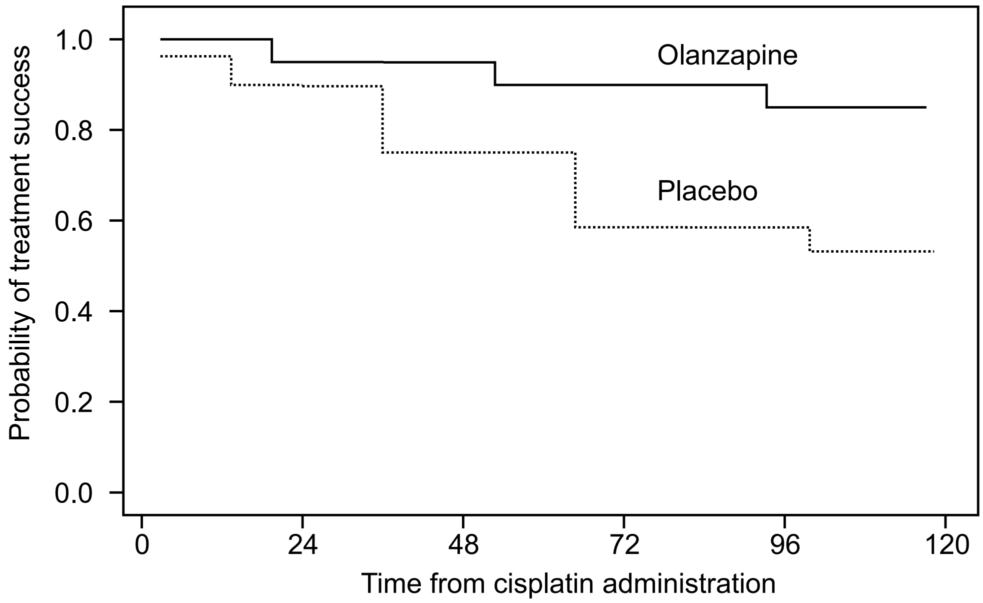

No. at Risk						
Olanzapine	XXX	XXX	XXX	XXX	XXX	XXX
Placebo	XXX	XXX	XXX	XXX	XXX	XXX

Figure. Kaplan–Meier Plot of the Time to Treatment Failure according to …

6.2.14 Patient-Reported Symptom in Daily Life from Day1 to 5

1） Purpose: To estimate and compare the distribution of patient reported symptom in daily life from day1 to 5 between groups.

2） Analysis set: Efficacy Analysis Set

3） Endpoint: Patient-reported symptom (appetite loss, sleepiness in the daytime, loss of concentration due to sleepiness, insomnia) in day1, 2, 3, 4 and 5 after chemotherapy.

4） Analysis method: Distribution of patient-reported symptom in the day 1, 2, 3, 4 and 5 will be estimated in each group and will be compared between groups using the Mantel test. In addition, the proportion of subjects with response “Not at all” will be estimated with risk difference model using GEE with an unstructured/exchangeable working correlation structures to examine treatment by time interaction. Point estimates and confidence intervals for the proportion will be calculated in each group and will be compared between groups for each time.

Table. Patient-Reported Symptom in Daily Life

	Olanzapine				Placebo				p value
	Not at all	A little bit	Quite a bit	Very much	Not at all	A little bit	Quite a bit	Very much	
**Appetite loss**									
Day 1	xx (xx%)	xx (xx%)	xx (xx%)	xx (xx%)	xx (xx%)	xx (xx%)	xx (xx%)	xx (xx%)	.xxx
Day 2	xx (xx%)	xx (xx%)	xx (xx%)	xx (xx%)	xx (xx%)	xx (xx%)	xx (xx%)	xx (xx%)	.xxx
Day 3	xx (xx%)	xx (xx%)	xx (xx%)	xx (xx%)	xx (xx%)	xx (xx%)	xx (xx%)	xx (xx%)	.xxx
Day 4	xx (xx%)	xx (xx%)	xx (xx%)	xx (xx%)	xx (xx%)	xx (xx%)	xx (xx%)	xx (xx%)	.xxx
Day 5	xx (xx%)	xx (xx%)	xx (xx%)	xx (xx%)	xx (xx%)	xx (xx%)	xx (xx%)	xx (xx%)	.xxx
**Sleepiness in the daytime**									
Day 1	xx (xx%)	xx (xx%)	xx (xx%)	xx (xx%)	xx (xx%)	xx (xx%)	xx (xx%)	xx (xx%)	.xxx
Day 2	xx (xx%)	xx (xx%)	xx (xx%)	xx (xx%)	xx (xx%)	xx (xx%)	xx (xx%)	xx (xx%)	.xxx
Day 3	xx (xx%)	xx (xx%)	xx (xx%)	xx (xx%)	xx (xx%)	xx (xx%)	xx (xx%)	xx (xx%)	.xxx
Day 4	xx (xx%)	xx (xx%)	xx (xx%)	xx (xx%)	xx (xx%)	xx (xx%)	xx (xx%)	xx (xx%)	.xxx
Day 5	xx (xx%)	xx (xx%)	xx (xx%)	xx (xx%)	xx (xx%)	xx (xx%)	xx (xx%)	xx (xx%)	.xxx
**Loss of concentration due to sleepiness**									
Day 1	xx (xx%)	xx (xx%)	xx (xx%)	xx (xx%)	xx (xx%)	xx (xx%)	xx (xx%)	xx (xx%)	.xxx
Day 2	xx (xx%)	xx (xx%)	xx (xx%)	xx (xx%)	xx (xx%)	xx (xx%)	xx (xx%)	xx (xx%)	.xxx
Day 3	xx (xx%)	xx (xx%)	xx (xx%)	xx (xx%)	xx (xx%)	xx (xx%)	xx (xx%)	xx (xx%)	.xxx
Day 4	xx (xx%)	xx (xx%)	xx (xx%)	xx (xx%)	xx (xx%)	xx (xx%)	xx (xx%)	xx (xx%)	.xxx
Day 5	xx (xx%)	xx (xx%)	xx (xx%)	xx (xx%)	xx (xx%)	xx (xx%)	xx (xx%)	xx (xx%)	.xxx
**Insomnia**									
Day 1	xx (xx%)	xx (xx%)	xx (xx%)	xx (xx%)	xx (xx%)	xx (xx%)	xx (xx%)	xx (xx%)	.xxx
Day 2	xx (xx%)	xx (xx%)	xx (xx%)	xx (xx%)	xx (xx%)	xx (xx%)	xx (xx%)	xx (xx%)	.xxx
Day 3	xx (xx%)	xx (xx%)	xx (xx%)	xx (xx%)	xx (xx%)	xx (xx%)	xx (xx%)	xx (xx%)	.xxx
Day 4	xx (xx%)	xx (xx%)	xx (xx%)	xx (xx%)	xx (xx%)	xx (xx%)	xx (xx%)	xx (xx%)	.xxx
Day 5	xx (xx%)	xx (xx%)	xx (xx%)	xx (xx%)	xx (xx%)	xx (xx%)	xx (xx%)	xx (xx%)	.xxx

Table. Distribution of NO Patient-Reported Symptom in Daily Life

	Olanzapine	Placebo	p value
**Appetite loss**			.xxx
Day 1	xx (xx%)	xx (xx%)	.xxx
Day 2	xx (xx%)	xx (xx%)	.xxx
Day 3	xx (xx%)	xx (xx%)	.xxx
Day 4	xx (xx%)	xx (xx%)	.xxx
Day 5	xx (xx%)	xx (xx%)	.xxx
**Sleepiness in the daytime**			.xxx
Day 1	xx (xx%)	xx (xx%)	.xxx
Day 2	xx (xx%)	xx (xx%)	.xxx
Day 3	xx (xx%)	xx (xx%)	.xxx
Day 4	xx (xx%)	xx (xx%)	.xxx
Day 5	xx (xx%)	xx (xx%)	.xxx
**Loss of concentration due to sleepiness**			.xxx
Day 1	xx (xx%)	xx (xx%)	.xxx
Day 2	xx (xx%)	xx (xx%)	.xxx
Day 3	xx (xx%)	xx (xx%)	.xxx
Day 4	xx (xx%)	xx (xx%)	.xxx
Day 5	xx (xx%)	xx (xx%)	.xxx
**Insomnia**			.xxx
Day 1	xx (xx%)	xx (xx%)	.xxx
Day 2	xx (xx%)	xx (xx%)	.xxx
Day 3	xx (xx%)	xx (xx%)	.xxx
Day 4	xx (xx%)	xx (xx%)	.xxx
Day 5	xx (xx%)	xx (xx%)	.xxx

6.2.15 Patient Satisfaction

1） Purpose: To estimate and compare the distribution of patient satisfaction between groups.

2） Analysis set: Efficacy Analysis Set

3） Endpoint: Patient satisfaction

4） Analysis method: Distribution of patient satisfaction will be estimated in each group and will be compared between groups using the Wilcoxon test. In addition, the proportion of subjects with response “Very Satisfied” or ”Satisfied” will be estimated. Point estimates and confidence intervals for the proportion will be calculated in each group and will be compared between groups for each time using Chi-Squared test. The proportion of subjects with “Very Satisfied” or ”Satisfied” or ”Somewhat” will also be estimated.

Table. Patient Satisfaction

	Olanzapine	Placebo	p value
Very satisfied	xx (xx%)	xx (xx%)	xxx
Satisfied	xx (xx%)	xx (xx%)	
Somewhat	xx (xx%)	xx (xx%)	
Rather satisfied	xx (xx%)	xx (xx%)	
Rather dissatisfied	xx (xx%)	xx (xx%)	
Dissatisfied	xx (xx%)	xx (xx%)	
Very dissatisfied	xx (xx%)	xx (xx%)	
“Very Satisfied” or ”Satisfied”	xx (xx%)	xx (xx%)	xxx
“Very Satisfied” or ”Satisfied” or ”Somewhat”	xx (xx%)	xx (xx%)	xxx

6.2.16 Numbers of Episodes of Vomiting and Nausea in the Overall Phase

1) Purpose: To explore numbers of vomiting and nausea episodes between treatment groups
2) Analysis set: Efficacy Analysis Set
3) Endpoint: Numbers of vomiting and nausea episodes in day1, 2, 3, 4 and 5 after chemotherapy.
4) Analysis method: Distribution of numbers of episodes of vomiting and nausea in the overall phase will be estimated and compared between treatment groups using Wilcoxon test. Numbers of vomiting and nausea episodes in day1, 2, 3, 4 and 5 will also be estimated. General linear model will be used to examine treatment by time interaction.

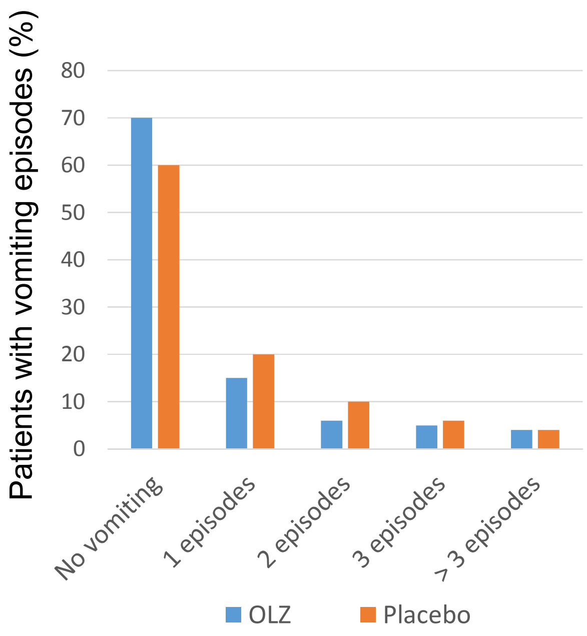

Figure. Patients Experiencing Vomiting Episodes in Overall Phase

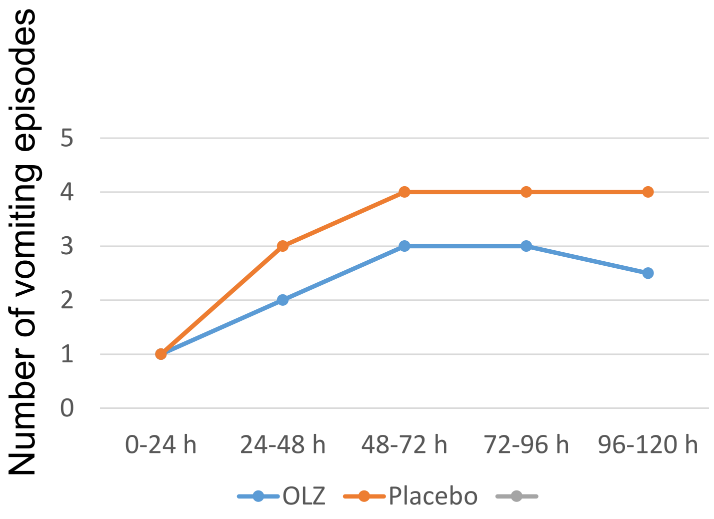

Figure. Pattern of Vomiting Incidence from Day 1 to Day5

6.2.17 Risk Factors for Delayed Vomiting and Nausea

1） Purpose: To explore risk factors for delayed vomiting and nausea

2） Analysis set: Efficacy Analysis Set

3） Endpoint: Vomiting and nausea

4） Analysis method: Risk difference model will be used to examine the relationship between the occurrence of delayed vomiting (nausea) and several risk factors; treatment group (olanzapine vs. placebo), gender (male vs. female), age (<55 vs. >=55 years), drinking habit (yes vs. no), motion sickness (yes vs. no), pregnancy sickness (yes vs. no), CDDP dose (<70 vs. >=70mg/m^2), occurrence of vomiting in acute phase (yes vs. no), occurrence of nausea in acute phase (yes vs. no).

Table. Risk Factors for Delayed Vomiting

Risk Factor	Univariate			Multivariate		
	Risk Difference	95% CI	p value	Risk Difference	95% CI	p value
Treatment group (Olanzapine vs. Placebo)						
Gender (male vs. female)						
Age (< vs. >=55 years)						
Drinking habit (yes vs. no)						
Motion sickness (yes vs. no)						
Pregnancy sickness (yes vs. no)						
CDDP dose (<70 vs. >=70mg/m^2)						
Occurrence of vomiting in acute phase (yes vs. no)						
Occurrence of nausea in acute phase (yes vs. no)						

Table. Risk Factors for Delayed Nausea

Risk Factor	Univariate			Multivariate		
	Risk Difference	95% CI	p value	Risk Difference	95% CI	p value
Treatment group (Olanzapine vs. Placebo)						
Gender (male vs. female)						
Age (< vs. >=55 years)						
Drinking habit (yes vs. no)						
Moton sickness (yes vs. no)						
Pregnancy sickness (yes vs. no)						
CDDP dose (<70 vs. >=70mg/m^2)						
Occurrence of vomiting in acute phase (yes vs. no)						
Occurrence of nausea in acute phase (yes vs. no)						

7. SAFETY ANALYSES

7.1 Adverse Events

7.1.1 Distribution of Adverse Events between Groups

1） Purpose: To estimate and compare the distribution of the grade of adverse events between groups.

2） Analysis set: Safety analysis set

3） Endpoint: CTCAE grade of adverse events (somnolence, insomnia, dry mouth, constipation, hiccups, dizziness)

4） Analysis method: Distribution of the grade of adverse events will be estimated in each group and will be compared between groups using the Mantel test.

Table. Treatment-Related Adverse Events

	Olanzapine			Placebo			p value
	Grade 1	Grade 2	Grade 3	Grade 1	Grade 2	Grade 3	
Somnolence	xx (xx%)	xx (xx%)	xx (xx%)	xx (xx%)	xx (xx%)	xx (xx%)	.xxx
Insomnia	xx (xx%)	xx (xx%)	xx (xx%)	xx (xx%)	xx (xx%)	xx (xx%)	.xxx
Dry mouth	xx (xx%)	xx (xx%)	xx (xx%)	xx (xx%)	xx (xx%)	xx (xx%)	.xxx
Constipation	xx (xx%)	xx (xx%)	xx (xx%)	xx (xx%)	xx (xx%)	xx (xx%)	.xxx
Hiccups	xx (xx%)	xx (xx%)	xx (xx%)	xx (xx%)	xx (xx%)	xx (xx%)	.xxx
Dizziness	xx (xx%)	xx (xx%)	xx (xx%)	xx (xx%)	xx (xx%)	xx (xx%)	.xxx

7.1.2 Subgroup Analysis for Distribution of Adverse Events between Groups

1） Purpose: To estimate and compare the distribution of the grade of adverse events between groups.

2） Analysis set: Safety analysis set

3） Endpoint: CTCAE grade of adverse events (somnolence, insomnia, dry mouth, constipation, hiccups, dizziness)

4） Analysis method: Distribution of the grade of adverse events will be estimated in each combination of treatment groups with several risk factors; gender (male vs. female), age (<65 vs. >=65 years), drinking habit (yes vs. no), motion sickness (yes vs. no), BSA (<1.5 vs. >=1.5m^2) and will be compared between groups using the Wilcoxon test.

Table. Treatment-Related Adverse Events according to …

	Olanzapine			Placebo			p value
	Grade 1	Grade 2	Grade 3	Grade 1	Grade 2	Grade 3	
Somnolence	xx (xx%)	xx (xx%)	xx (xx%)	xx (xx%)	xx (xx%)	xx (xx%)	.xxx
Insomnia	xx (xx%)	xx (xx%)	xx (xx%)	xx (xx%)	xx (xx%)	xx (xx%)	.xxx
Dry mouth	xx (xx%)	xx (xx%)	xx (xx%)	xx (xx%)	xx (xx%)	xx (xx%)	.xxx
Constipation	xx (xx%)	xx (xx%)	xx (xx%)	xx (xx%)	xx (xx%)	xx (xx%)	.xxx
Hiccups	xx (xx%)	xx (xx%)	xx (xx%)	xx (xx%)	xx (xx%)	xx (xx%)	.xxx
Dizziness	xx (xx%)	xx (xx%)	xx (xx%)	xx (xx%)	xx (xx%)	xx (xx%)	.xxx

7.1.3 Relationship between Somnolence and Nausea Grading

1） Purpose: To examined the relation between somnolence and nausea grading

2） Analysis set: Safety analysis set

3） Endpoint: CTCAE grade of somnolence and nausea

4） Analysis method: Relationship between somnolence and nausea grading will be described. Spearman's correlation coefficient will be estimated and Cochran-Mantel-Haenszel test will be used to test the correlation.

Table. Relationship between Somnolence Grade and Nausea

Somnolence	Grade 1	Grade 2	Grade 3	Grade 4	Correlation Coefficient p value
Nausea					
Grade 1	xx (xx%)	xx (xx%)	xx (xx%)	xx (xx%)	
Grade 2	xx (xx%)	xx (xx%)	xx (xx%)	xx (xx%)	
Grade 3	xx (xx%)	xx (xx%)	xx (xx%)	xx (xx%)	
Grade 4	xx (xx%)	xx (xx%)	xx (xx%)	xx (xx%)	

7.1.4 Sleepiness in the overall phase

1） Purpose: To explore sleepiness between treatment groups

2） Analysis set: Efficacy Analysis Set

3） Endpoint: Sleepiness in day1, 2, 3, 4 and 5 after chemotherapy.

4） Analysis method: Distributions of sleepiness items in day1, 2, 3, 4 and 5 will be estimated and compared between treatment groups using Mantel test. In addition, the proportion of subjects with response "None" will be estimated with risk difference model using GEE

with an unstructured/exchangeable working correlation structures to examine treatment by time interaction. Point estimates and confidence intervals for the proportion will be calculated in each group and will be compared between groups for each time.

Table. Incidences of Sleepiness From Day 1 to 5

	Olanzapine				Placebo				p value
	None	Mild	Moderate	Severe	None	Mild	Moderate	Severe	
Day 1	xx (xx%)	xx (xx%)	xx (xx%)	xx (xx%)	xx (xx%)	xx (xx%)	xx (xx%)	xx (xx%)	.xxx
Day 2	xx (xx%)	xx (xx%)	xx (xx%)	xx (xx%)	xx (xx%)	xx (xx%)	xx (xx%)	xx (xx%)	.xxx
Day 3	xx (xx%)	xx (xx%)	xx (xx%)	xx (xx%)	xx (xx%)	xx (xx%)	xx (xx%)	xx (xx%)	.xxx
Day 4	xx (xx%)	xx (xx%)	xx (xx%)	xx (xx%)	xx (xx%)	xx (xx%)	xx (xx%)	xx (xx%)	.xxx
Day 5	xx (xx%)	xx (xx%)	xx (xx%)	xx (xx%)	xx (xx%)	xx (xx%)	xx (xx%)	xx (xx%)	.xxx

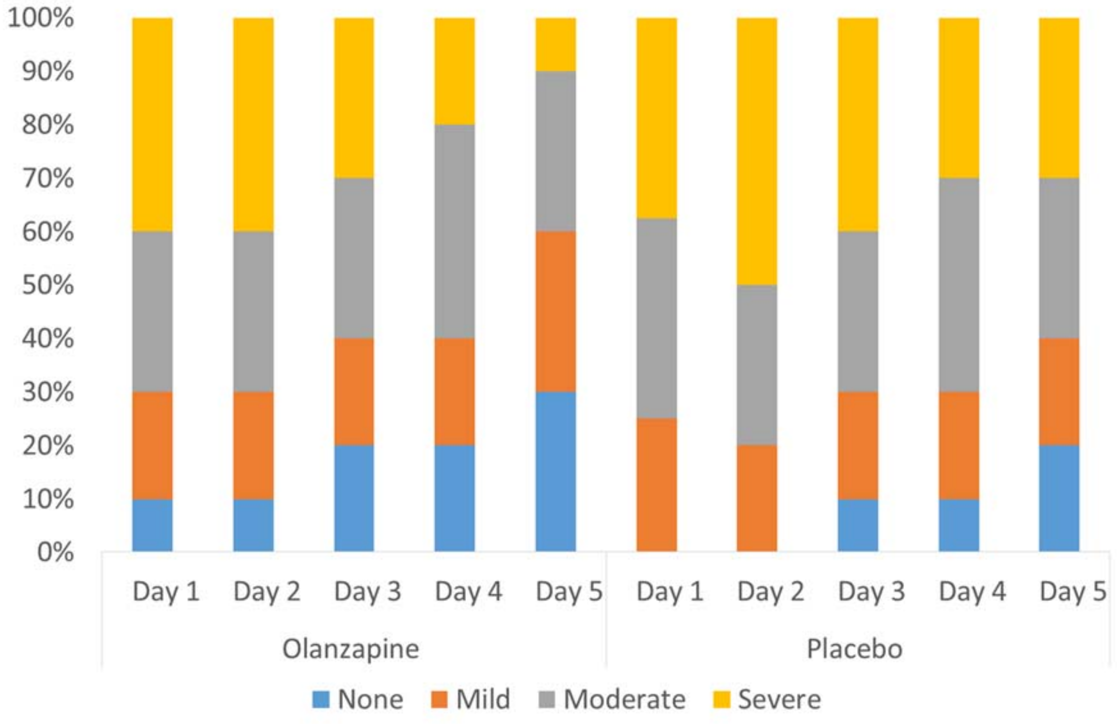

Figure. Incidences of Sleepiness From Day 1 to 5

Table. Distribution of NO Sleepiness From Day 1 to 5

	Olanzapine	Placebo	p value
**Sleepiness**			.xxx
Day 1	xx (xx%)	xx (xx%)	.xxx
Day 2	xx (xx%)	xx (xx%)	.xxx
Day 3	xx (xx%)	xx (xx%)	.xxx
Day 4	xx (xx%)	xx (xx%)	.xxx
Day 5	xx (xx%)	xx (xx%)	.xxx

8. REVISION HISTORY

Version	Date	Changes
1.0	20-12-2018	
