# Supplementary material for: Efficacy of Olanzapine in Addition to Standard Triplet Antiemetic Therapy for Cisplatin-Based Chemotherapy: A Secondary Analysis of the J-FORCE Randomized Clinical Trial
Source: JAMA Netw Open. 2023 May 2;6(5):e2310894. doi: 10.1001/jamanetworkopen.2023.10894 (PMC10155068; doi:10.1001/jamanetworkopen.2023.10894)
Supplement: Supplement 2. — eFigure 1. Kaplan-Meier Plot Showing the Time to Treatment Failure According to Allocation Adjustment Factors eFigure 2. Kaplan-Meier Curve Showing the Time to Treatment Failure According to Patients’ Risk Factors eFigure 3. CONSORT Diagram eTable 1. The Proportion of Patients Achieving a Complete Response, Complete Control, and Total Control According to Allocation Adjustment Factors eTable 2. The Proportion of Patients Achieving a Complete Response, Complete Control, and Total Control According to Patients’ Risk Factors [file jamanetwopen-e2310894-s002.pdf]

Supplemental Online Content

Abe M, Yamaguchi T, Fujita Y, et al. Efficacy of olanzapine in addition to standard triplet antiemetic therapy for cisplatin-based chemotherapy: a secondary analysis of the J-FORCE randomized clinical trial. *JAMA Netw Open*. 2023;6(5):e2310894. doi:10.1001/jamanetworkopen.2023.10894

eFigure 1. Kaplan-Meier Plot Showing the Time to Treatment Failure According to Allocation Adjustment Factors

eFigure 2. Kaplan-Meier Curve Showing the Time to Treatment Failure According to Patients' Risk Factors

eFigure 3. CONSORT Diagram

eTable 1. The Proportion of Patients Achieving a Complete Response, Complete Control, and Total Control According to Allocation Adjustment Factors

eTable 2. The Proportion of Patients Achieving a Complete Response, Complete Control, and Total Control According to Patients' Risk Factors

This supplemental material has been provided by the authors to give readers additional information about their work.

© 2023 Abe M et al. *JAMA Network Open.*

eFigure 1

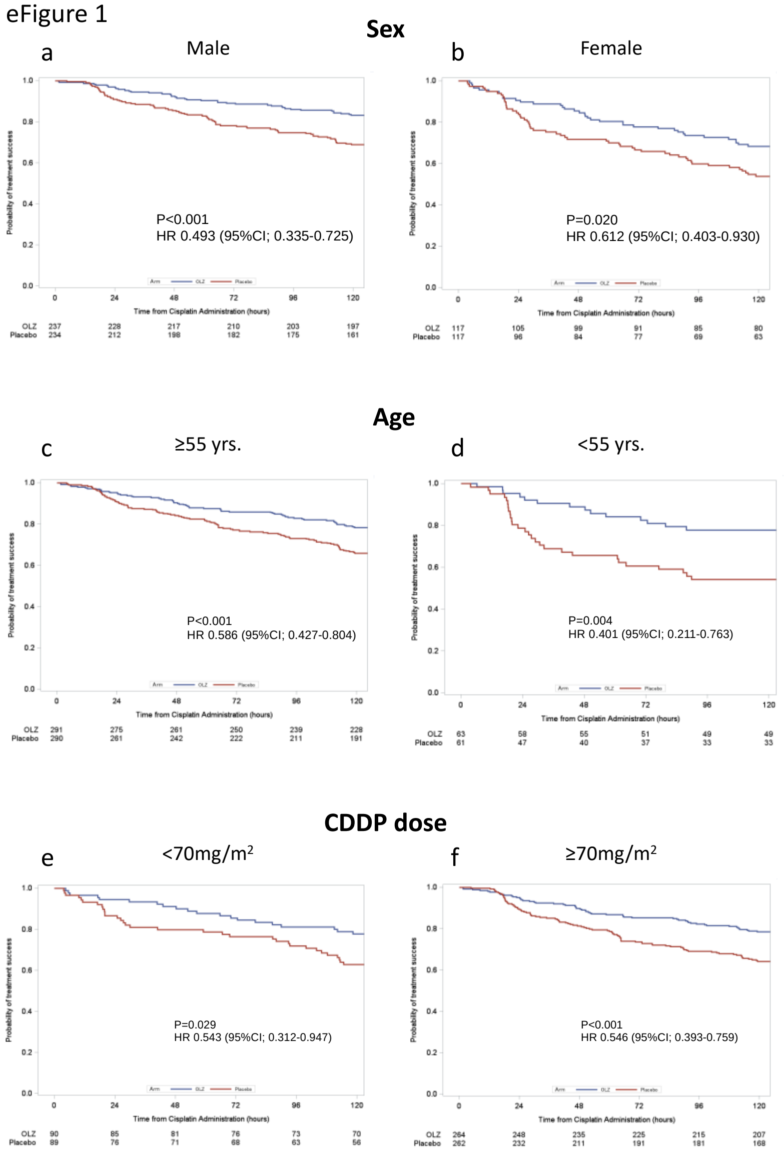

eFigure 1. Kaplan-Meier plot showing the time to treatment failure according to allocation adjustment factors. a. Male, b. Female, c. Age ≥55 years, d. Age <55 years, e. CDDP <70 mg/m^2, f. CDDP ≥70 mg/m^2. CI, confidence interval; HR, hazard ratio; OLZ, olanzapine

© 2023 Abe M et al. *JAMA Network Open.*

eFigure 2

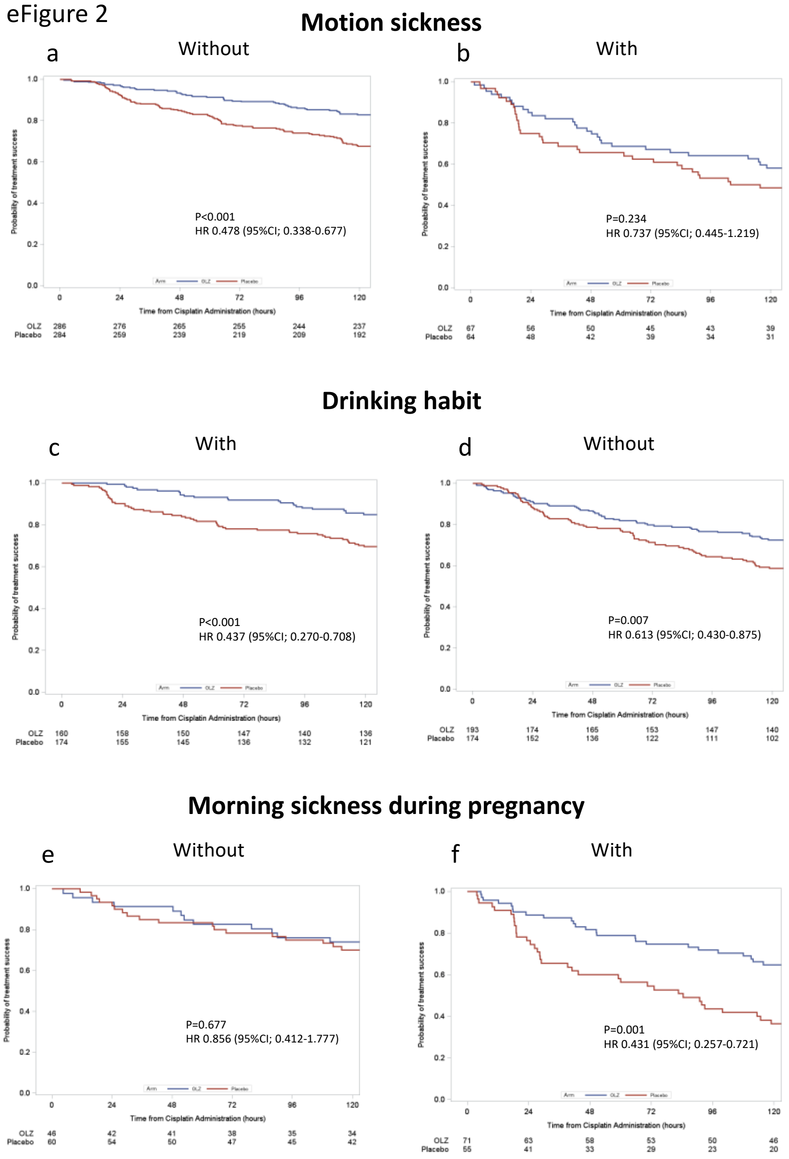

eFigure 2. Kaplan-Meier curve showing the time to treatment failure according to patients' risk factors. a. Without a history of motion sickness, b. With a history of motion sickness, c. With a drinking habit, d. Without a drinking habit, e. Without a history of morning sickness during pregnancy (Female), f. With a history of morning sickness during pregnancy (Female). CI, confidence interval; HR, hazard ratio; OLZ, olanzapine

© 2023 Abe M et al. *JAMA Network Open.*

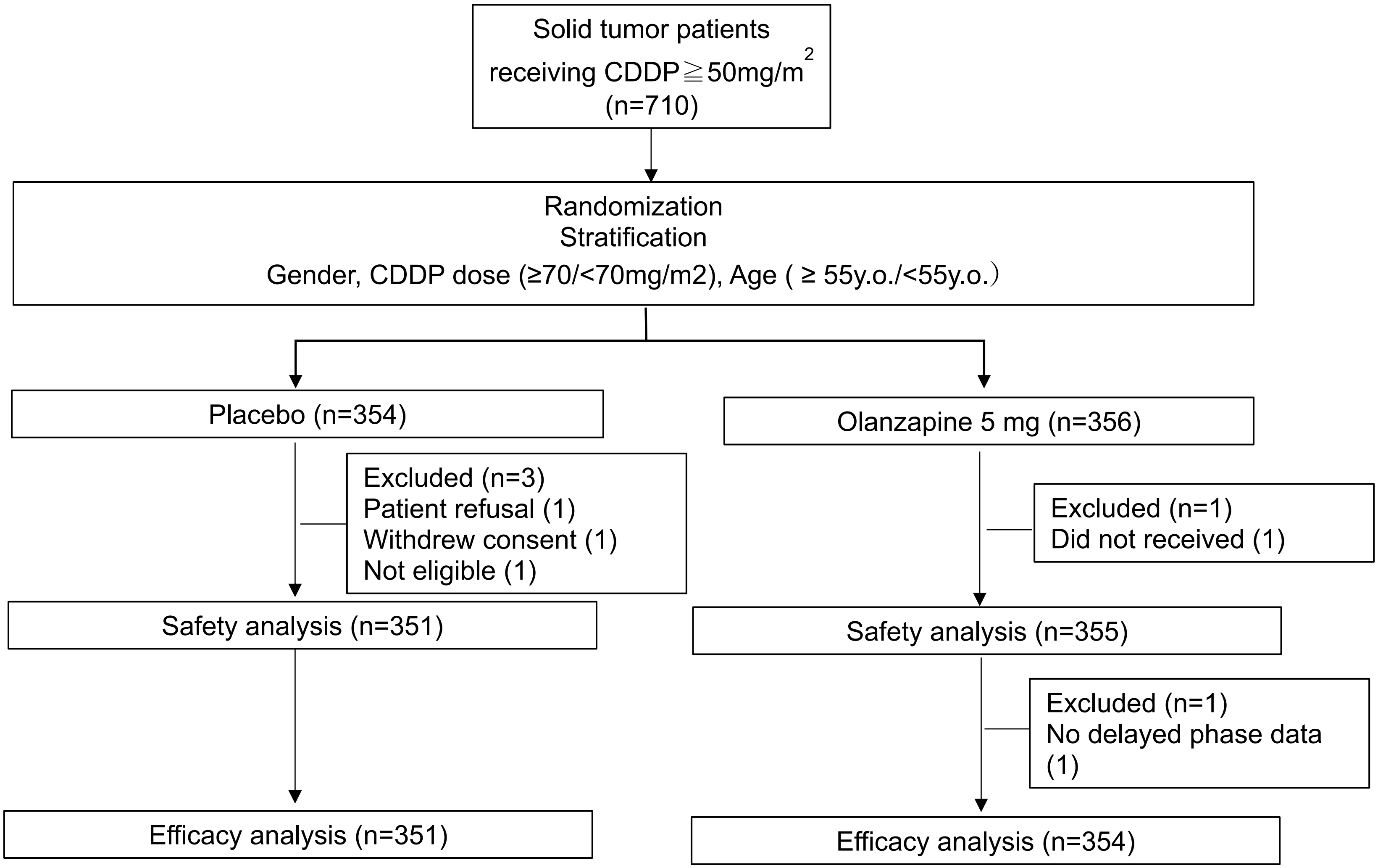

eFigure 3. CONSORT Diagram

© 2023 Abe M et al. *JAMA Network Open.*

eTable 1. The Proportion of Patients Achieving a Complete Response, Complete Control, and Total Control According to Allocation Adjustment Factors

				Olanzapine		Placebo		Risk difference		P value
				n (%)	95% CI	n (%)	95% CI	Estimate	95% CI	
Sex	CR	Acute	Male	230 (97.1)	94.9-99.2	213 (91.0)	87.4-94.7	6.0%	1.8-10.6	0.006
			Female	106 (90.6)	85.3-95.9	98 (83.8)	77.1-90.4	6.8%	-1.9, 15.6	0.119
			Risk difference	6.4%	0.7-12.2	7.3%	-0.4, 14.9	p = 0.867		
		Delayed	Male	197 (83.1)	78.4-87.9	165 (70.5)	64.7-76.4	12.6%	5.0-20.1	0.001
			Female	83 (70.9)	62.7-79.2	66 (56.4)	47.4-65.4	14.5%	2.2-26.3	0.021
			Risk difference	12.2%	2.7-21.7	14.1%	3.4-24.8	p = 0.793		
	CC	Acute	Male	229 (96.6)	94.3-98.9	213 (91.0)	87.4-94.7	5.6%	1.2-10.3	0.012
			Female	104 (88.9)	83.2-94.6	96 (82.1)	75.1-89.0	6.8%	-2.3, 16.0	0.139
			Risk difference	7.7%	1.6-13.9	9.0%	1.1-16.8	p = 0.808		
		Delayed	Male	193 (81.4)	76.5-86.4	159 (67.9)	62.0-73.9	13.5%	5.7-21.1	<0.001
			Female	83 (70.9)	62.7-79.2	64 (54.7)	45.7-63.7	16.2%	3.9-28.0	0.010
			Risk difference	10.5%	0.9-20.1	13.3%	2.4-24.1	p = 0.709		
	TC	Acute	Male	216 (91.1)	87.5-94.8	195 (83.3)	78.6-88.1	7.8%	1.8-13.9	0.011
			Female	88 (75.2)	67.4-83.0	88 (75.2)	67.4-83.0	0.0%	-11.0, 11.0	1.000
			Risk difference	15.9%	7.3-24.6	8.1%	-1.0, 17.3	p = 0.224		
		Delayed	Male	157 (66.2)	60.2-72.3	127 (54.3)	47.9-60.7	12.0%	3.1-20.6	0.008
			Female	56 (47.9)	38.8-56.9	49 (41.9)	32.9-50.8	6.0%	-6.7, 18.4	0.359
			Risk difference	18.4%	7.5-29.3	12.4%	1.4-23.4	p = 0.448		
Age	CR	Acute	≥55 yrs.	277 (95.2)	92.7-97.7	263 (90.7)	87.4-94.0	4.5%	0.3, 8.9	0.034
			<55 yrs.	59 (93.7)	87.6-99.7	48 (78.7)	68.4-89.0	15.0%	2.7-27.4	0.016
			Risk difference	1.5%	-5.0, 8.0	12.0%	1.2-22.8	p = 0.104		
		Delayed	≥55 yrs.	229 (78.7)	74.0-83.4	196 (67.6)	62.2-73.0	11.1%	3.9-18.2	0.003
			<55 yrs.	51 (81.0)	71.3-90.7	35 (57.4)	45.0-69.8	23.6%	7.3-38.3	0.005
			Risk difference	-2.3%	-13.0, 8.5	10.2%	-3.3, 23.7	p = 0.158		

© 2023 Abe M et al. *JAMA Network Open.*

	CC	Acute	**≥55 yrs.**	275 (94.5)	91.9-97.1	262 (90.3)	87.0-93.7	4.2%	-0.2, 8.6	0.059
			**<55 yrs.**	58 (92.1)	85.4-98.7	47 (77.1)	66.5-87.6	15.0%	2.2-27.8	0.021
			**Risk difference**	2.4%	-4.7, 9.6	13.3%	2.2, 24.4	p = 0.107		
		Delayed	**≥55 yrs.**	225 (77.3)	72.5-82.1	191 (65.9)	60.4-71.3	11.5%	4.1-18.6	0.002
			**<55 yrs.**	51 (81.0)	71.3-90.7	32 (52.5)	39.9-65.0	28.5%	12.0-43.1	<0.001
			**Risk difference**	-3.6%	-14.5, 7.2	13.4%	-0.3, 27.1	p = 0.056		
	TC	Acute	**≥55 yrs.**	254 (87.3)	83.5-91.1	241 (83.1)	78.8-87.4	4.2%	-1.6, 10.0	0.156
			**<55 yrs.**	50 (79.4)	69.4-89.4	42 (68.9)	57.2-80.5	10.5%	-4.9, 25.4	0.183
			**Risk difference**	7.9%	-2.8, 18.6	14.3%	1.9, 26.7	p = 0.449		
		Delayed	**≥55 yrs.**	182 (62.5)	57.0-68.1	151 (52.1)	46.3-57.8	10.5%	2.4-18.3	0.011
			**<55 yrs.**	31 (49.2)	36.9-61.6	25 (41.0)	28.6-53.3	8.2%	-9.1, 24.8	0.360
			**Risk difference**	13.3%	-0.2, 26.9	11.1%	-2.5, 24.7	p = 0.818		
CDDP dose	CR	Acute	**<70mg/m2**	85 (94.4)	89.7-99.2	77 (86.5)	79.4-93.6	7.9%	-0.9, 17.1	0.071
			**≥70mg/m2**	251 (95.1)	92.5-97.7	234 (89.3)	85.6-93.1	5.8%	1.2, 10.5	0.014
			**Risk difference**	-0.6%	-6.0, 4.8	-2.8%	-10.8, 5.2	p = 0.661		
		Delayed	**<70mg/m2**	72 (80.0)	71.7-88.3	60 (67.4)	57.7-77.2	12.6%	-0.3, 25.0	0.056
			**≥70mg/m2**	208 (78.8)	73.9-83.7	171(65.3)	59.5-71.0	13.5%	5.9, 21.0	<0.001
			**Risk difference**	1.2%	-8.4, 10.8	2.1%	-9.2, 13.5	p = 0.902		
	CC	Acute	**<70mg/m2**	85 (94.4)	89.7-99.2	76 (85.4)	78.1-92.7	9.1%	0.1, 18.4	0.045
			**≥70mg/m2**	248 (93.9)	91.1-96.8	233 (88.9)	85.1-92.7	5.0%	0.2, 10.0	0.040
			**Risk difference**	0.5%	-5.0, 6.0	-3.5%	-11.8, 4.7	p = 0.426		
		Delayed	**<70mg/m2**	72 (80.0)	71.7-88.3	59 (66.3)	56.5-76.1	13.7%	0.7, 26.2	0.039
			**≥70mg/m2**	204 (77.3)	72.2-82.3	164 (62.6)	56.7-68.5	14.7%	6.9, 22.3	<0.001
			**Risk difference**	2.7%	-7.0, 12.4	3.7%	-7.7, 15.1	p = 0.899		
	TC	Acute	**<70mg/m2**	72 (80.0)	71.7-88.3	70 (78.7)	70.1-87.2	1.4%	-10.5, 13.2	0.824
			**≥70mg/m2**	232 (87.9)	83.9-91.8	213 (81.3)	76.6-86.0	6.6%	0.4, 12.8	0.037
			**Risk difference**	-7.9%	-17.0, 1.3	-2.6%	-12.4, 7.1	p = 0.443		
		Delayed	**<70mg/m2**	52 (57.8)	47.6-68.0	45 (50.6)	40.2-61.0	7.2%	-7.3, 21.3	0.334

© 2023 Abe M et al. *JAMA Network Open.*

**≥70mg/m2**	161 (61.0)	55.1-66.9	131(50.0)	44.0-56.1	11.0%	2.5, 19.3	0.011
**Risk difference**	-3.2%	-15.0, 8.6	0.6%	-11.5, 12.6	p = 0.661		

The number of patients in olanzapine and placebo groups, respectively, that were: males: 237, 234; females: 117, 117; ≥55 years: 291, 290;<55 years: 63, 61; CDDP <70 mg/m^2: 90, 89; CDDP ≥70 mg/m^2: 264, 262. CR: complete response, CC: complete control, TC: total control, CDDP: cisplatin, CI: confidence interval.

© 2023 Abe M et al. *JAMA Network Open.*

eTable 2. The Proportion of Patients Achieving a Complete Response, Complete Control, and Total Control According to Patients' Risk Factors

				Olanzapine		Placebo		Difference		P value
				n (%)	95% CI	n (%)	95% CI	Estimate	95% CI	
Motion sickness	CR	Acute	Yes	57 (85.1)	76.5-93.6	48 (75.0)	64.4-85.6	10.1%	-3.7, 23.6	0.150
			No	278 (97.2)	95.3-99.1	262 (92.3)	89.1-95.4	5.0%	1.3, 8.9	0.008
			Difference	12.1%	3.4, 20.9	17.3%	6.2, 28.3	p = 0.476		
		Delayed	Yes	41 (61.2)	49.5-72.9	33 (51.6)	39.3-63.8	9.6%	-7.2, 25.7	0.268
			No	239 (83.6)	79.3-87.9	198 (69.7)	64.4-75.1	13.9%	6.9-20.6	<0.001
			Difference	22.4%	9.9, 34.8	18.2%	4.8, 31.5	p = 0.651		
	CC	Acute	Yes	57 (85.1)	76.5-93.6	47 (73.4)	62.6-84.3	11.6%	-2.3, 25.3	0.101
			No	275 (96.2)	93.9-98.4	261 (91.9)	88.7-95.1	4.3%	0.3, 8.4	0.032
			Difference	11.1%	2.3, 19.9	18.5%	7.2, 29.7	p = 0.312		
		Delayed	Yes	41 (61.2)	49.5-72.9	31 (48.4)	36.2-60.7	12.8%	-4.2, 28.7	0.144
			No	235 (82.2)	77.7-86.6	191 (67.6)	62.2-73.1	14.6%	7.5-21.5	<0.001
			Difference	21.0%	8.5, 33.5	19.2%	5.8, 32.6	p = 0.847		
	TC	Acute	Yes	47 (70.2)	59.2-81.1	40 (62.5)	50.6-74.4	7.7%	-8.4, 23.2	0.356
			No	256 (89.5)	86.0-93.1	242 (85.2)	81.1-89.3	4.3%	-1.2, 9.8	0.123
			Difference	19.4%	7.8, 30.9	22.7%	10.2, 35.3	p = 0.700		
		Delayed	Yes	25 (37.3)	25.7-48.9	19 (29.7)	18.5-40.9	7.6%	-8.4, 23.1	0.357
			No	188 (65.7)	60.2-71.2	157 (55.3)	49.5-61.1	10.5%	2.4-18.3	0.011
			Difference	28.4%	15.6, 41.2	25.6%	13.0, 38.2	p = 0.758		
Drinking habit	CR	Acute	Yes	159 (99.4)	96.6-99.98*	157 (90.2)	85.8-94.6	9.2%	4.6-14.5	<0.001
			No	176 (91.2)	87.2-95.2	153 (87.9)	83.1-92.8	3.3%	-3.1, 9.8	0.307
			Difference	8.2%	4.0, 12.4	2.3%	-4.3, 8.8	p = 0.138		

© 2023 Abe M et al. *JAMA Network Open.*

		Delayed	**Yes**	137 (85.6)	80.2-91.1	123 (70.7)	63.9-77.5	14.9%	6.1-23.4	0.001
			**No**	143 (74.1)	67.9-80.3	108 (62.1)	54.9-69.3	12.0%	2.5-21.3	0.014
			**Difference**	11.5%	3.3, 19.8	8.6%	-1.3, 18.5	p = 0.657		
	**CC**	**Acute**	**Yes**	159 (99.4)	96.6-99.98*	156 (89.7)	85.1-94.2	9.7%	5.1-15.2	<0.001
			**No**	173 (89.6)	85.3-93.9	152 (87.4)	82.4-92.3	2.3%	-4.3, 9.1	0.494
			**Difference**	9.7%	5.3, 14.2	2.3%	-4.4, 9.0	p = 0.070		
		**Delayed**	**Yes**	135 (84.4)	78.8-90.0	119 (68.4)	61.5-75.3	16.0%	6.9-24.7	<0.001
			**No**	141 (73.1)	66.8-79.3	104 (59.8)	52.5-67.1	13.3%	3.6-22.7	0.007
			**Difference**	11.3%	2.9, 19.7	8.6%	-1.4, 18.7	p = 0.687		
	**TC**	**Acute**	**Yes**	150 (93.8)	90.0-97.5	145 (83.3)	77.8-88.9	10.4%	3.6-17.3	0.003
			**No**	153 (79.3)	73.6-85.0	137 (78.7)	72.7-84.8	0.5%	-7.8, 8.9	0.899
			**Difference**	14.5%	7.6, 21.3	4.6%	-3.6, 12.8	p = 0.070		
		**Delayed**	**Yes**	107 (66.9)	59.6-74.2	99 (56.9)	49.5-64.3	10.0%	-0.5, 20.1	0.061
			**No**	106 (54.9)	47.9-61.9	77 (44.3)	36.9-51.6	10.7%	0.4-20.6	0.042
			**Difference**	12.0%	1.8, 22.1	12.6%	2.2, 23.1	p = 0.926		
**Morning sickness during pregnancy (Female)**	**CR**	**Acute**	**Yes**	63 (88.7)	81.4-96.1	42 (76.4)	65.1-87.6	12.4%	-0.9, 26.2	0.066
			**No**	43 (93.5)	82.1-98.6*	55 (91.7)	84.7-98.7	1.8%	-10.1, 12.5	0.728
			**Difference**	4.8%	-5.5, 15.0	15.3%	2.1, 28.5	p = 0.216		
		**Delayed**	**Yes**	49 (69.0)	58.3-79.8	23 (41.8)	28.8-54.9	27.2%	9.7-42.6	0.002
			**No**	34 (73.9)	61.2-86.6	42 (70.0)	58.4-81.6	3.9%	-13.5, 20.2	0.659
			**Difference**	4.9%	-11.7, 21.5	28.2%	10.7, 45.6	p = 0.058		
	**CC**	**Acute**	**Yes**	62 (87.3)	79.6-95.1	41 (74.6)	63.0-86.1	12.3%	-9.0, 26.9	0.067
			**No**	42 (91.3)	83.2-99.5	54 (90.0)	82.4-97.6	1.3%	-11.5, 12.7	0.821
			**Difference**	4.0%	-7.3, 15.2	15.5%	1.7, 29.2	p = 0.206		
		**Delayed**	**Yes**	49 (69.0)	58.3-79.8	23 (41.8)	28.8-54.9	27.2%	9.7, 42.6	0.002
			**No**	34 (73.9)	61.2-86.6	40 (66.7)	54.7-78.6	7.3%	-10.5, 23.7	0.423
			**Difference**	4.9%	-11.7, 21.5	24.9%	7.2, 42.5	p = 0.107		

© 2023 Abe M et al. *JAMA Network Open.*

TC	Acute	**Yes**	50 (70.4)	59.8-81.0	42 (72.7)	61.0-84.5	-2.3%	-17.5, 13.7	0.777
		**No**	38 (82.6)	71.7-93.6	47 (78.3)	67.9-88.8	4.3%	-11.6, 18.8	0.586
		**Difference**	12.2%	-3.1, 27.4	5.6%	-10.1, 21.3	p = 0.556		
	Delayed	**Yes**	32 (45.1)	33.5-56.6	18 (32.7)	20.3-45.1	12.3%	-4.8, 28.2	0.162
		**No**	24 (52.2)	37.7-66.6	30 (50.0)	37.4-62.7	2.2%	-16.5, 20.6	0.825
		**Difference**	7.1%	-11.4, 25.6	17.3%	-0.4, 35.0	p = 0.437		

The number of patients in the olanzapine and placebo groups, respectively that: experienced motion sickness: 67, 64; did not experience motion sickness: 286, 284; had a drinking habit: 160, 174; did not have a drinking habit: 193, 174; experienced pregnancy-related illness: 71, 55; did not experience pregnancy-related illness: 46, 60.

CR: complete response, CC: complete control, TC: total control.

© 2023 Abe M et al. *JAMA Network Open.*
