# Supplementary material for: Efficacy of Olanzapine in Addition to Standard Triplet Antiemetic Therapy for Cisplatin-Based Chemotherapy: A Secondary Analysis of the J-FORCE Randomized Clinical Trial
Source: JAMA Netw Open. 2023 May 2;6(5):e2310894. doi: 10.1001/jamanetworkopen.2023.10894 (PMC10155068; doi:10.1001/jamanetworkopen.2023.10894)
Supplement: Supplement 3. — Data Sharing Statement [file jamanetwopen-e2310894-s003.pdf]

Data Sharing Statement

Abe. Efficacy of Olanzapine in Addition to Standard Triplet Antiemetic Therapy for Cisplatin-Based Chemotherapy. *JAMA Netw Open*. Published May 02, 2023.
doi:10.1001/jamanetworkopen.2023.10894

Data
Data available: Yes
Data types: Deidentified participant data
How to access data: hhashimo@ncc.go.jp
When available: With publication

Supporting Documents
Document types: Statistical/analytic code
How to access documents: Hironobu Hashimoto hhashimo@ncc.go.jp
When available: With publication

Additional Information
Who can access the data: any investigator requesting the data
Types of analyses: for any purpose or a specified purpose
Mechanisms of data availability: with investigator support
